# Do You Match What I Match? A Systematic Review of Over 20 Years of the Emotional Face Matching Task Reveals Vast Inconsistencies in Design and Implementation

**DOI:** 10.1002/hbm.70636

**Published:** 2026-09-21

**Authors:** Hannah S. Savage, Linda Schlüter, Roberta Russo, Imogen Leaning, Christian F. Beckmann, Andre F. Marquand

**Affiliations:** ^1^ Donders Institute of Brain, Cognition and Behaviour Radboud University Nijmegen the Netherlands; ^2^ Department of Medical Neuroscience Radboud University Medical Centre Nijmegen the Netherlands; ^3^ Institute of Cognitive Neuroscience University College London London UK; ^4^ Centre for Functional MRI of the Brain (FMRIB), Nuffield Department of Clinical Neurosciences, Wellcome Centre for Integrative Neuroimaging University of Oxford Oxford UK

## Abstract

We performed a systematic review of over 20 years of literature that implemented a variant of the emotional face matching task (EFMT). We aimed to quantify the heterogeneity in tasks used across studies, and to summarise the regions reported as activated when participants match the target face stimuli, as compared to matching the control stimuli, within and between patients with a mental health diagnosis and unaffected controls. Our results highlight the vast heterogeneity in all aspects of task design and implementation across studies. Our high‐level synthesis of brain regions activated broadly confirms the involvement of the amygdala during emotional face matching, but shows that an increase in amygdala activation is inconsistent both within and across mental health conditions. We further highlight increased activation within the cingulate cortex as a transdiagnostic alteration (vs. controls) during emotional face matching. We introduce solutions to address the challenge of heterogeneity, including a standard version of the EFMT and applicable statistical methods, and provide recommendations for the field to progress our understanding of emotional face processing.

## Graphical abstract

We systematically reviewed over 20 years of literature using the emotional face matching task (EFMT), revealing vast heterogeneity in task design, implementation, and reported neural activation. We introduce solutions to address the challenge of heterogeneity, including a standard version of the EFMT and the use of applicable statistical methods.

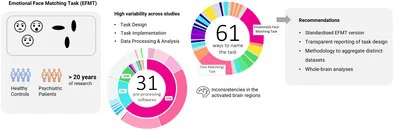

## 1 Introduction

In a seminal manuscript published in 2000, Hariri et al. (2000) used an fMRI task to probe emotional face processing—often since referred to as the ‘Hariri task’ and hereafter named the emotional face matching task (EFMT)—which gained particular popularity when they introduced an adapted version of the EFMT in a widely cited manuscript in 2002 (Hariri et al. 2002). While undergoing fMRI, participants were required to match one of two images (black and white photographs of facial configurations consistent with the common view of prototypic facial expressions of fear or anger) that were simultaneously presented at the bottom of the screen, with a third target image displayed at the top of the screen. Participants also matched emotionally neutral pictures, and performed a sensorimotor control during which they matched black‐filled geometric shapes. Comparing participants' neural activation while matching faces to that while matching shapes, Hariri et al. identified a main effect of the task within the bilateral amygdala.

The original task and its evolutions have since been implemented in many studies due to its robust activation of so‐called ‘emotional circuitry’ and its simple design facilitating its use in clinical populations (Quintana et al. 2011; Wang et al. 2004; Piggot et al. 2004; Fakra et al. 2008; Peluso et al. 2009; Cardoner et al. 2011; Fonzo et al. 2015; Jamieson et al. 2021; Via et al. 2014; Tessitore et al. 2005; Ferri et al. 2014; Bangen et al. 2014; Smith 2012; Hulvershorn et al. 2013; Labus et al. 2013; Prather et al. 2013). Generally speaking, studies report that matching faces, as compared to matching shapes, engages the amygdala, fusiform face area, anterior insula cortex, the pregenual and dorsal anterior cingulate cortex, the dorsomedial and dorsolateral prefrontal cortex, and visual input areas (Morris et al. 1996; Haxby et al. 2002, 2000; Fusar‐Poli et al. 2009). Activity within the amygdala is thought to be so robust that studies, including large‐scale neuroimaging initiatives such as the Human Connectome Project, have used a variant of the EFMT as a functional localiser (Barch et al. 2013; Leitão et al. 2022; Foland‐Ross et al. 2010). Arguably, the EFMT remains the most widely utilised task in functional neuroimaging, and it continues to play a pivotal role in many large‐scale prospective studies.

Studies proceeding Hariri et al.'s (2002) publication have each altered small components of the task design to better answer their specific research questions. Generally, however, the implications of such modifications on task‐evoked activation have not been assessed empirically. Prior studies specifically designed to address these differences do report that modifications, such as changing the target and control stimuli, rephrasing the task instructions, or changing the matching rule result in differences in activation (Geissberger et al. 2020; Habel et al. 2007; Lange et al. 2003), and it is possible these changes may have gone so far as to alter the psychological process engaged. For example, instructing participants to engage with affective stimulus components (e.g., match emotional expressions) is likely to target explicit emotion processing, whereas directing attention to non‐affective attributes (i.e., matching gender or identity) is thought to probe implicit emotion processes (Scheuerecker et al. 2007). In addition to the aforementioned methodological variability, there is considerable variation with regards to the study population, with some studies selectively recruiting participants in order to investigate how task‐related processes vary by developmental stage or mental‐health condition, for example. By assuming that the findings of independent studies are directly comparable and derivative, we have overlooked the opportunity to comprehensively understand the influence of these study design choices and uncover nuances in the underlying psychobiological processes, as well as to understand differences in functional activation between (and within) individuals.

Coinciding with the broader adaptation of the task following the more widely cited in Hariri et al. (2000), we aimed to perform a systematic review of over 20 years of literature (2002–2026) that has implemented the EFMT, or one of its variants since. We aimed to quantify the heterogeneity in implemented tasks, and to summarise the regions reported as activated when matching the target face stimuli, as compared to matching the control stimuli, in both control participants and participants with mental health conditions. We aimed to relate changes in task design and demographic factors to task activation, in the hope that through a greater understanding of their influence we will uncover a more comprehensive understanding of emotional face processing in health and ill‐health. With the knowledge we gained from performing this review, we conclude with recommendations for the field including considerations for future studies and methods to address or embrace heterogeneity to progress our understanding of emotional face processing.

## 2 Method

This review was conducted in line with the preregistered protocol (https://doi.org/10.17605/OSF.IO/RUZ7D). See Figure S1 for a PRISMA flow diagram and Table S1 for an overview of the studies included (*n* = 179). Briefly, full, original research articles published in peer‐reviewed journals were selected in which human participants completed an fMRI version of the EFMT (as defined below). Research participants were either (a) healthy controls (as defined by the study) or (b) participants who had, at the time of participating, a formal diagnosis and/or who were experiencing high levels of disorder‐related symptoms (as defined by the study using clinically relevant cut‐off scores of validated questionnaires) of one or more mood disorders (Major Depressive Disorder [MDD]; Bipolar Disorder [BP]; Premenstrual Dysphoric Disorder [PMDD]), anxiety and anxiety‐related disorders (Social Anxiety Disorder [SAD], Generalised Anxiety Disorder [GAD], Separation Anxiety Disorder [Sep AD], Specific Phobia [SP], Panic Disorder with/without agoraphobia [PD], Obsessive‐Compulsive Disorder [OCD], Posttraumatic Stress Disorder [PTSD]), neurodevelopmental disorders (Autism Spectrum Disorder [ASD], Attention Deficit Hyperactivity Disorder [ADHD]), schizophrenia spectrum disorders (schizophrenia, schizotypal and delusional disorder), as well as Body Dysmorphic Disorder (BDD), Gender Dysphoria (GD), Borderline Personality Disorder (BPD), and Substance Use Disorder (SUD). We defined the task of interest as one in which participants were required to match one of two simultaneously presented images of facial expressions with a third target image (FACES condition) and perform an appropriate sensorimotor control (CONTROL condition) while undergoing fMRI.

### 2.1 Study Identification

#### 2.1.1 Search Terms

The search terms included: (1) a first clause relating to the functional imaging task used [‘emotional face matching’, ‘emotional face processing’, ‘facial emotion processing’, ‘face matching’, ‘emotion matching’], (2) a second clause relating to the use of fMRI [‘Magnetic resonance’, ‘Functional magnetic resonance imaging’, ‘fMRI’], and (3) a third clause/filter (depending on the database) which restricted the search to the date range of interest [2002–2026]. Where possible, a (4) fourth clause/filter (depending on the database) was applied to restrict the search to original research articles. See Supporting Information 2.1.1 for the combined string, for each database. We also screened papers that cited the Hariri et al. (2002) manuscript (as indexed by Scopus) in an attempt to capture studies that referred to the task as ‘an adaptation of Hariri et al. (2002)’, and we performed citation chaining during data extraction, in cases where studies referred to previous implementations of the task in references that were not already included.

#### 2.1.2 Screening

Abstract and title screening was completed within the Rayyan.ai online platform, by four authors (Savage, Leaning, Schlüter, Russo). Included papers proceeded to the full text screening. In cases where reviewers disagreed, the study was discussed until a consensus was reached.

### 2.2 Data Extraction

As detailed in the pre‐registered protocol, we (Savage, Schlüter, Russo) extracted descriptors of the records,^1^ sample characteristics and demographic details per group of interest, MRI acquisition parameters, task parameter details, regions reported as activated in the contrasts of interest [(1) controls: faces > shapes, (2) patients: faces > shapes, (3) patients > controls: faces > shapes, (4) patients < controls: faces > shapes], if regions were specified as regions of interest (i.e., for small volume correction [SVC]/region of interest [ROI] thresholding), and the statistical threshold that was applied.

#### 2.2.1 Data Synthesis

The names used to refer to the task were extracted and names that were deemed very similar were grouped and standardised to a single name. For a full table of terms that were standardised, see Table S2. Reported task stimuli expressions were harmonised by combining synonyms and selecting the most commonly used label, yielding 9 unique labels: fearful, angry, happy, neutral, surprised, sad, disgust, contempt, or positive. For each contrast of interest, the names of the reported brain regions were extracted. It was noted, where applicable, which regions were specified as regions of interest (i.e., for small volume correction/ROI thresholding), and the statistical threshold that was applied (coded 1–9). For each threshold we quantified how many times manuscripts reported searching for, and/or finding activation within a given region. Where necessary the reported activation was combined into functional regions or clearly defined brain structures that included both left and right hemispheres. See Supporting Information 2.2.1 and Table S3a–f for detailed information on data synthesis of activated brain regions.

### 2.3 Deviations From Pre‐Registration

Due to unexpectedly high heterogeneity across studies, there was insufficient consistency to draw meaningful conclusions regarding how paradigm design choices influence task‐evoked activation in controls or patient groups, which was our first pre‐registered primary outcome. Relatedly, we were insufficiently powered to probe the relation between or influence of factors such as age and sex, or different target facial stimuli on activation, as interpretation would be confounded by the aforementioned heterogeneity in task design; we were thus unable to address the secondary outcome we pre‐registered. Very few papers reported the second [controls > faces] or fourth contrast of interest [controls > patients for faces > control], and we therefore chose not to analyse this data further. Lastly, because reporting practices were highly inconsistent and relevant information could not be extracted systematically, we did not quantitatively assess adherence to fMRI reporting guidelines (Poldrack et al. 2008) using the extraction table adapted by Guo et al. (2014), as originally planned. Instead, we provide a brief qualitative assessment of the extent to which these guidelines were followed, based on our observations during data extraction.

### 2.4 Data and Code Availability

The data extraction table and all code to run the analysis and generate the plots in the manuscript can be found at https://github.com/predictive‐clinical‐neuroscience/EFMT_Review. The standardised version of the EFMT is available at https://github.com/Hannah‐Savage/Standard_EFMT.

## 3 Results

See Supporting Information 3 and Figure S2 for a note on the terminology used in this manuscript.

### 3.1 Study Samples

#### 3.1.1 Sample Size

**FIGURE 1:**
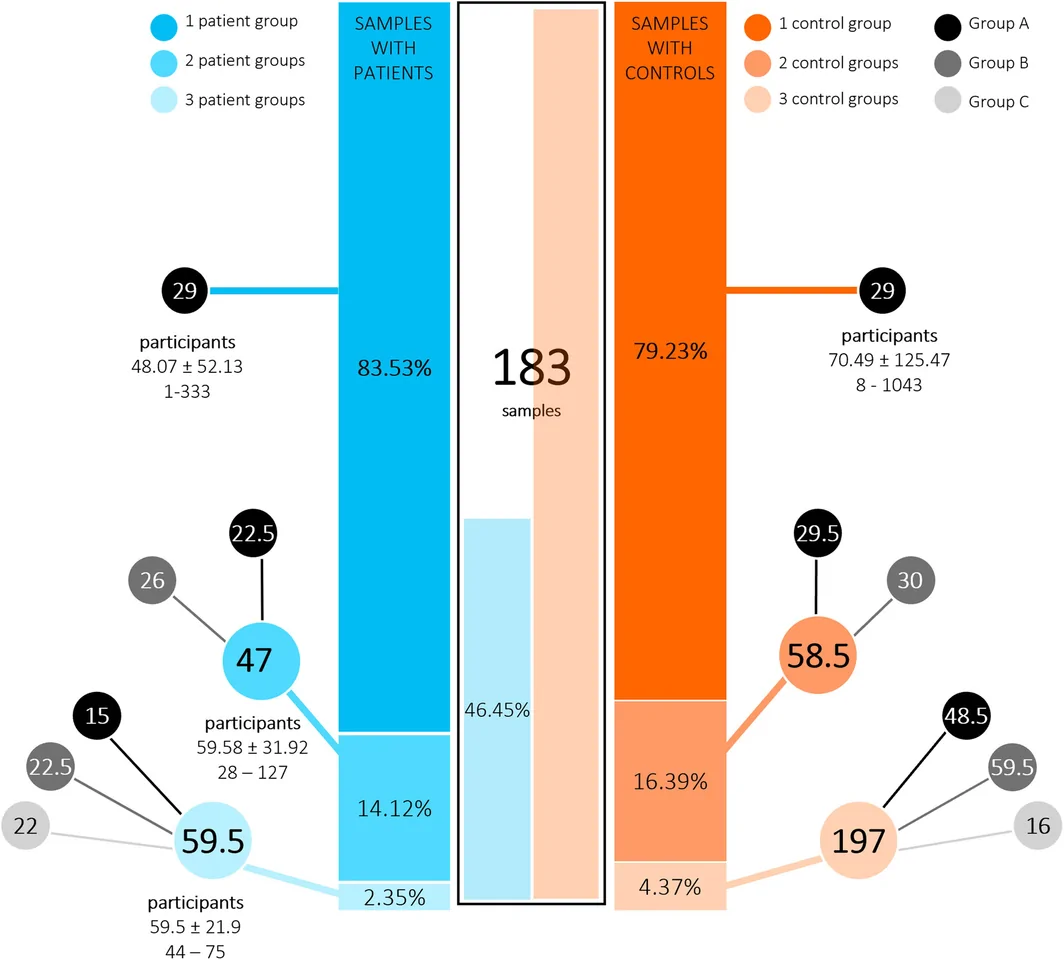
Schematic of number and size of control and patient groups in included articles. The percentage of studies with 1, 2, or 3 groups in 98 control samples (right; orange) and 85 patient samples (left; blue). The median sample size of each sample (for samples with 2 or more groups) and group is illustrated in respective circles (Group A—black, Group B—dark grey, Group C—light grey).

Ninety‐eight (53.55%) samples included only control participants, and 85 (46.45%) compared controls to at least one patient group of interest. Overall, control groups had a median of 30 participants (mean ± SD = 62.44 ± 107.97). One hundred and forty‐five samples (79.23%) had only one control group, 30 samples (16.39%) had two control groups, and 8 samples (4.37%) had three control groups. Of the 87 samples that included patients, 71 (83.53%) had only one patient group, 12 samples (14.12%) had two patient groups, and 2 samples (2.35%) had three patient groups, wherein the median patient group size was 24.5 participants (mean ± SD = 38.99 ± 41.67). The respective sample sizes of each control and patient group can be found in Table 1 and Figure 1.

**TABLE 1:** Summary age, sex, and sample size information for samples included in the review.

			Mean age (±SD) [range]^a^	Mean % females/women participants (±SD%)	Median sample size (mean ± SD) [range]	Diagnoses (% of 85 clinical samples)	
Controls 98 samples	Overall		30.68 (±15.64)	52.31% (±25.02)	30.00 (62.44 ± 107.97) [6–1043]		
	1 control group	Control group 1	28.40 (±10.80)	54.46% (±24.44)	30.00 (68.85 ± 122.78) [8–1043]		
	2 control groups	Control group 1	25.42 (±8.78)	43.56% (±26.87)	29.50 (45.47 ± 51.58) [11–247]		
		Control group 2	28.43 (±11.17)	47.19% (±27.27)	30.00 (51.20 ± 99.47) [11–561]		
	3 control groups	Control group 1	29.53 (±6.53)	65.62% (±25.48)	48.50 (62.0 ± 51.31) [20–176]		
		Control group 2	30.32 (±7.07)	51.23% (±15.55)	59.50 (60.25 ± 28.29) [19–108]		
		Control group 3	29.61 (±6.26)	58.65% (±11.69)	16.00 (33.75 ± 32.71) [6–100]		
Patients 85 samples	Overall		30.50 (±9.58)	53.02% (±26.86)	24.50 (38.99 ± 41.67) [1–333]	Neurodivergence (ADHD, ASD) Anxiety (‐related) disorder Mood disorder (MDD, BP) Schizophrenia Other (BDD, BPD, GD, SUD) Comorbid	9 groups (10.58%) 18 groups (21.17%) 39 groups (45.88%) 10 groups (11.76%) 6 groups (7.06%) 16 groups (18.82%)
	1 patient group	Patient group 1	30.02 (±10.78)	51.98% (±29.11)	29.0 (47.32 ± 51.25) [1–333]		
	2 patient groups	Patient group 1	32.67 (±8.08)	56.86% (±20.98)	22.5 (28.92 ± 15.23) [14–61]		
		Patient group 2	35.02 (±6.58)	54.04% (±21.42)	26.00 (30.67 ± 17.06) [13–66]		
	3 patient groups	Patient group 1	32.47 (±2.07)	73.33% (±9.43)	15.00 (15.0 ± 0.0) [15–15]		
		Patient group 2	30.5 (±4.95)	65.00% (±2.36)	22.50 (22.5 ± 10.61) [15–30]		
		Patient group 3	30.22 (±6.77)	65.71% (±8.08)	22 (22.0 ± 11.31) [14–30]		

^a^ Age data was not reported for 9 control groups.

#### 3.1.2 Sex/Gender

We have reported samples as including female/women and males/men in the following summaries (recognising the incorrect conceptualization of these labels as binary, see Footnote a).

**FIGURE 2:**
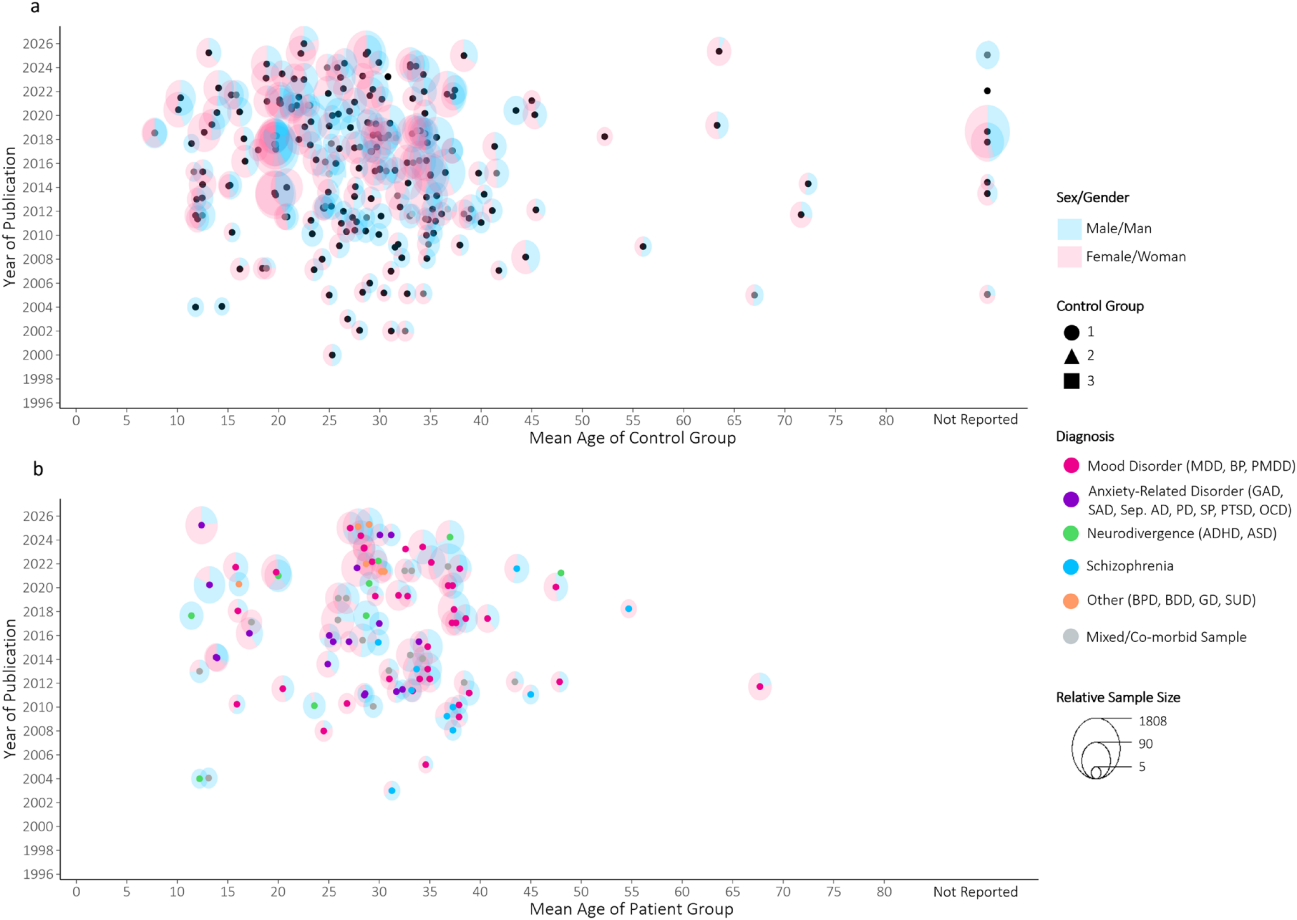
Age and Sex/Gender of control and patient groups in included articles. Each point illustrates the mean age of the participant group and year of publication for manuscripts included in the review. The radius of the pie‐charts is proportional to the sample size of the group (where, radius = log(SampleSize/3)), and the blue and red shading indicates the proportion of males and females within each sample. (a) Control groups: The shape indicates which group is being referenced. Samples with one control group are represented by a circle, and otherwise control group two, or three are represented by a triangle or a square, respectively. The mean age of the sample was not reported for 9 control groups. (b) Patient groups: The color of the inner circle signifies the primary diagnosis of the patient group. More specifically, mood disorders are coded as pink, anxiety‐related disorders as purple, neurodivergent conditions as green, schizophrenia as blue, and ‘other’ (which includes Borderline Personality Disorder, Body Dysmorphic Disorder, Gender Dysphoria and Substance Use Disorder) as orange. Where samples were composed of a heterogenous group of diagnoses, this is coded as light grey.

On average, 52.31% (±25.02%; median: 54.88%) of control groups were female/women participants and this proportion remained relatively consistent when samples were separated by the number of control groups within each sample (percentages of females then ranged from 43.56% to 65.62%; see Table 1 and Figure 2a). Twenty‐four control groups included only males/men (19 studies), and 12 groups included only females/women (13 studies).

Overall, patient groups were similarly relatively equally split between female/women and males/men, with an average of 53.02% (±26.86%; median: 58.56%) females/women per group. This proportion qualitatively skewed towards a greater number of females/women when samples were divided by the number of control groups (percentages of females then ranged from 51.98% to 73.33%; see Table 1). Eight patient groups, 5 of which focused on neurodivergence, included only males/men (8 studies), 8 groups included only females/women (9 studies), and 1 group included only trans boys (hereafter coded in all analyses as men). See Table 1 and Figure 2b for an overview for each patient group.

#### 3.1.3 Age

The median mean age of control groups was 28.66 years (mean ± SD = 30.68 ± 15.64), with mean age ranging from 7.75 to 72.3 years (see Figure 2a). The respective median and mean ± SD of each control group can be found in Table 1. Mean age was not reported for 9 control groups (7 manuscripts), and the standard deviation of the groups' ages was not reported for 24 groups (18 studies).

For patients, the median mean age was 30.73 (mean ± SD = 30.50 ± 9.58), with mean age ranging from 11.40 to 67.70 years. For the distribution of age across the diagnostic categories see Figure 2b.

### 3.2 Task Parameters

#### 3.2.1 Task Names

**FIGURE 3:**
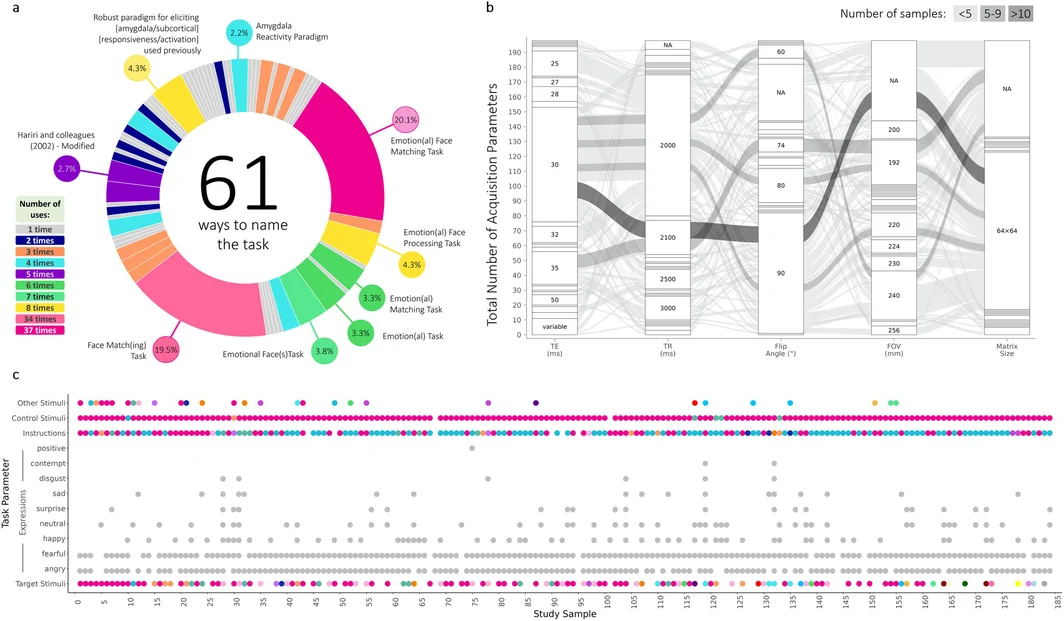
Heterogeneity of task name and parameters in included articles/samples. (a) Naming of the task: In the 183 included manuscripts, there were 61 unique names used to refer to the task. The most frequently used are highlighted with their respective percentage as a proportion of the number of included manuscripts, and a full list can be found in Table S4. (b) Task stimuli and instructions: Each row of dots represents the stimuli used in a task (total *n* = 184). For each task parameter (column) a different colour indicates a different stimuli from the original task design. Note there is no inherent ordering to the colours; rather, they reflect different categorical variables, for example, illustrating that two studies differed in their use of the Ekman series face stimuli or the NimStim‐Set. A legend can be found in the Figure S3. (c) Task structure: Each bar illustrates the relative proportion of tasks (total *n* = 184) that use the respective value of the different task component (faces/controls blocks/trials, trials per block). The width and opacity of each stream between the bars represents the total number of tasks with those parameters.

After standardising very similar names, there were 61 unique ways that articles referred to this task. The ‘Emotion(al) Face Matching Task’ and ‘Face Match(ing) Task’ were the most common names, used in 37 (20.10%) and 34 (18.48%) manuscripts, respectively. Some articles did not refer to the task by name, rather stating that they used ‘a robust paradigm for eliciting [amygdala/subcortical] [responsiveness/activation] used previously’ (*n* = 8, 4.35%), ‘a modified version of a task by Hariri et al.’ followed by a citation of the original manuscript by Hariri and colleagues (*n* = 5, 2.71%). For a full list and relative frequency of task names see Figure 3a and Table S4.

#### 3.2.2 Face Stimuli—Stimuli Set

Studies most frequently presented the Ekman Series stimulus set (*n* = 63, 34.23% samples) (Ekman 1976) or the NimStim‐Set (*n* = 21, 11.41%) (Tottenham et al. 2009) to participants. Other stimuli included: stimuli from Gur et al. (2002) (*n* = 10, 5.43%), and the Karolinska Directed Emotional Faces Set (*n* = 7, 3.80%) (Lundqvist et al. 1998). There were in total 18 unique stimulus sets (including the use of single and combined sets) presented in the studies included in the review. Fifty‐nine samples (32.07%) did not report the stimulus set with which they presented participants in their study (see Table S5a).

#### 3.2.3 Face Stimuli—Expressions

Facial configurations consistent with the common view of prototypic facial expressions of fear, anger, happiness, neutral, surprise, sadness and disgust were used in 170 (92.39%), 158 (85.87%), 58 (31.52%), 43 (23.37%), 21 (11.41%), 17 (9.24%) and 6 samples (3.26%) respectively. Two samples (1.09%) reported using contempt faces and one sample used positive faces. Seven samples (3.80%) did not report the expressions shown by their stimuli. Almost half of the samples combined angry and fearful faces (*n* = 87, 47.28%), with some adding happy (*n* = 29, 15.76%), neutral (*n* = 5, 2.71%), or both happy and neutral faces (*n* = 7, 3.80%) to those presented. In total, there were 25 unique combinations of expressions presented to participants, which included between 1 and 8 prototypic expressions (see Table S5b).

#### 3.2.4 Instructions

Across the samples there were 8 different target instructions provided to participants. In 90 samples (48.91%) participants were instructed to match faces, while in 65 samples (35.32%) they were told to match emotions. Other studies asked participants to match expression (*n* = 8, 4.34%), match identity (*n* = 5, 2.72%), match image (*n* = 4, 2.17%), match gender (*n* = 3, 1.64%), match affect (*n* = 2, 1.09%), or match emotional valence (*n* = 1, 0.54%). Six samples did not report the instructions provided to participants (3.26%). Seventeen samples reported multiple instructions (9.24%; either providing multiple instructions for the single target condition, or where relevant to be applied to the ‘other’ condition). These additional instructions related to labelling (label emotion, *n* = 8, 4.35%; identify emotions, *n* = 1, 0.54%; label expression, *n* = 1, 0.54%), and other matching contingencies: match identity (*n* = 3, 1.63%), match sex (*n* = 2, 1.09%), match gender (*n* = 2, 1.09%), match pictures (*n* = 1, 0.54%). See Tables S6a and S6b for a full overview of the primary and secondary instructions.

#### 3.2.5 Control Stimuli

One hundred and seventy‐six of 184 samples (95.65%) used geometric shapes as the somatomotor control stimuli. Four samples used non‐social control stimuli (houses: *n* = 2, 1.09%; fruit and vegetable, *n* = 1, 0.54%; neutral scenes, *n* = 1, 0.54%), and 2 presented rectangles filled with either pixelated patterns derived from neutral face pictures (*n* = 1, 0.54%) or patterns of circles or squares (*n* = 1, 0.54%). Two samples did not explain the control stimulus used (1.09%; see Table S7).

#### 3.2.6 Other Stimuli

One hundred and fifty‐four samples (83.70%) did not have an additional stimulus category/condition. Of the 30 samples that did, 25 samples (13.59%) used face stimuli and 16 of those asked participants to perform a different task to the target condition (i.e., label emotion, match gender/sex, match identity etc.). Six samples (3.26%) used scenes (IAPS, *n* = 4, 2.17%; unspecified threatening scenes, *n* = 2, 1.09%), and others used images of doors (*n* = 1, 0.54%), IAPs images of threats of natural or artificial origin (*n* = 1, 0.54%) or a fixation cross (*n* = 1, 0.54%; see Table S8). An overview of task stimuli and instructions used can be found in Figure 3b.

### 3.3 Task Structure

See Figure 3c for an alluvial plot illustrating the frequency of the use of each task component.

#### 3.3.1 Number of Blocks

Face trials were most frequently presented in 4 blocks (*n* = 67; 36.41%), and 5 control blocks (*n* = 62; 33.70%); note that the samples in these percentages for each stimulus do not necessarily correspond to one another. The overall design of the task did vary largely across the samples, with face blocks being frequently presented 2, 9, 3 and 6 times (*n* = 30, 16.30%; *n* = 18, 9.78%; *n* = 15, 8.15%; *n* = 13, 7.07%, respectively). Similarly, control blocks were frequently presented 3, 4, 6 or 9 times (*n* = 36, 19.57%; *n* = 20, 10.87%; *n* = 15, 8.15%; *n* = 11, 5.98% respectively). Ten samples (5.43%) did not report the number of face blocks and 11 (5.98%) did not report the number of control blocks (see Tables S9a and S9b for an overview). The most frequently used ratios of faces:control blocks were 4:5 (*n* = 45, 24.46%), 4:4 (*n* = 17, 9.24%) and 2:3 (*n* = 16, 8.70%).

#### 3.3.2 Block Duration

Forty‐three samples had blocks of 30 s duration (23.37%), and 19 presented blocks that lasted 20 s (10.33%). Twenty‐seven samples (14.67%) had different lengths for blocks of faces, compared to control stimuli with the most frequent respective durations being 48 s and 36 s per condition (*n* = 14, 7.61%; see Table S9c).

#### 3.3.3 Number of Trials

Face stimuli were most frequently presented a total of 24, 12 or 36 times (*n* = 62, 33.69% of samples; *n* = 29, 15.76%; *n* = 16, 9.78% respectively), however this value ranged from 6 to 96 (*n* = 1 each, 0.54%) across the review. Control stimuli were most frequently presented a total of 30 times (*n* = 52, 28.26% of samples). Other samples frequently presented the control stimuli 18, 24 or 36 times (*n* = 31, 16.85%; *n* = 18, 9.78%; *n* = 15, 8.15%, respectively), and presentations ranged from 6 to 80 trials (*n* = 1 each, 0.54%). Four (2.17%) and five (2.72%) samples, respectively related to face and control stimuli, allowed participants to progress through the task at their own pace (self‐paced), and as such did not report the specific number of trials for either condition as it varied between individuals. Eighteen samples (9.78%) did not report how many times they presented face stimuli, and 24 (13.04%) did not report the number of control stimuli presented (see Tables S10a and S10b).

#### 3.3.4 Trial Duration

Stimuli (faces and control) were most frequently presented for a duration of 5 s (*n* = 65, 35.32%), or 4 s (*n* = 52, 28.26%). Shorter durations than 4 s were used in 24 studies and 10 samples used durations longer than 5 s. Twenty studies did not report trial duration. See Table S10c for a complete overview.

### 3.4 Scanning Parameters

Of the 184 scans referenced in manuscripts included in this review, 32 different MRI scanners were used. Siemens was the most popular vendor, with their machines used for 47.63% of the MRI scans. Scanners with four different field strengths were used, with 3 T MRI scanners representing the most common (162 times, 88.05%). Among the studies included, some samples conducted multiple scanning sessions with distinct scanning parameters, resulting in a total of 198 acquisition parameter settings used. In total, there were 162 unique combinations of scanning parameters. There were only 10 cases that reported every parameter expected in line with good quality reporting, all of which were unique combinations. See Supporting Information 3.1 for a description of the scanning parameters and processing software used.

### 3.5 Brain Regions Activated

**FIGURE 4:**
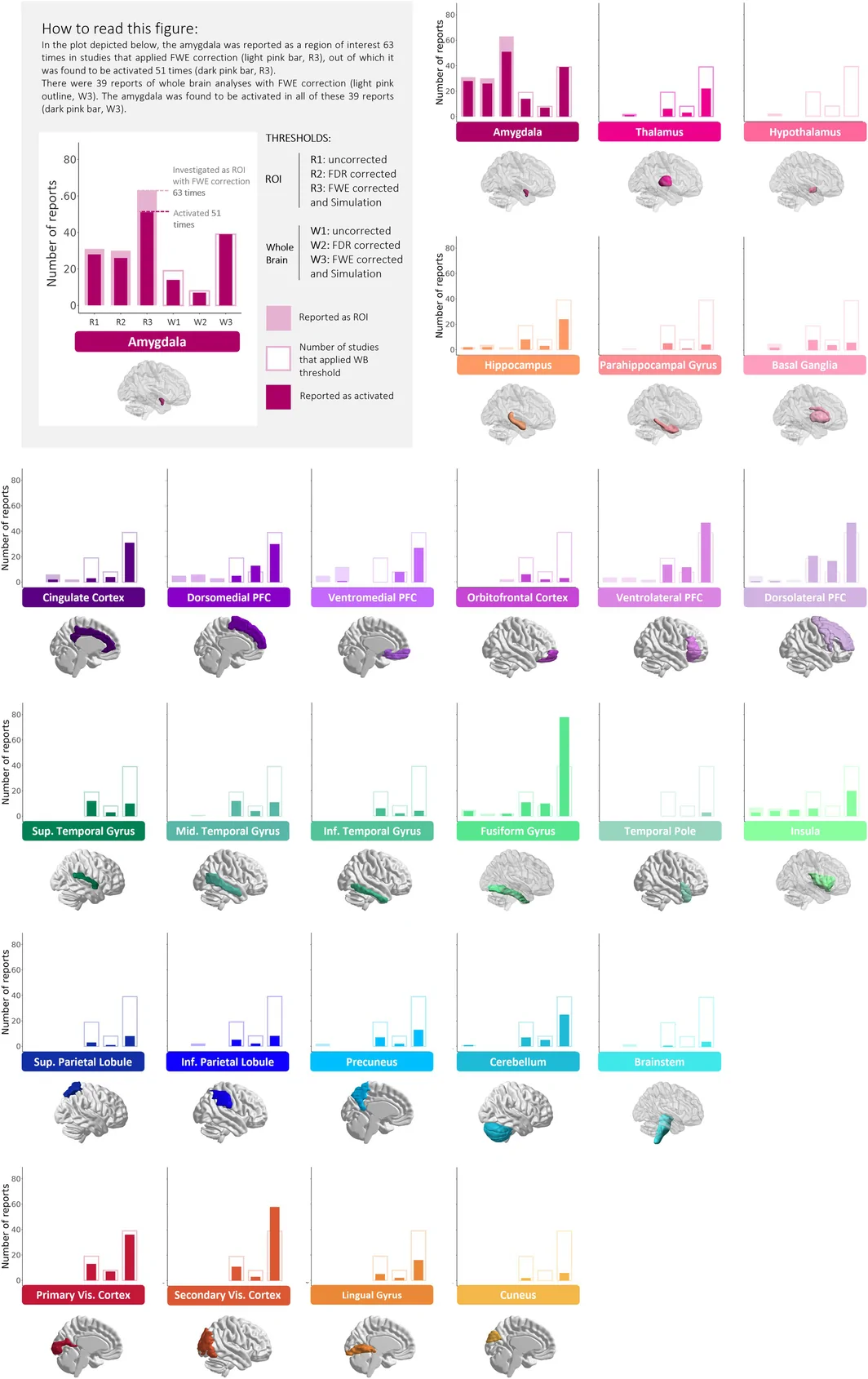
Regional search and activation results for the faces > shapes contrast at various statistical thresholds in control participants. Bar graphs illustrate the number of times a region (named below, and highlighted within a glass brain) was reported to be searched for (lightly shaded) and/or reported as activated (full colour) in the faces greater than shapes contrast, for six statistical thresholds. For whole brain (WB) analyses, empty bars indicate how many studies applied this threshold; where the number of reports of a region being activated is greater than the number of studies, this indicates that reports of subregional activation were combined within the larger region illustrated.

For the sake of brevity and comprehension, we selectively report a synthesis of the results from the 190 contrasts extracted from our included manuscripts. For each of our 28 specified brain regions of interest (ROIs; see Methods for the synthesis of regions) we report how often the region was (i) specified as a ROI, and (ii) whether it was reported to be activated in response to faces as compared to shapes at different statistical thresholds. We remind readers that as the summary counts of ROIs are often composed of multiple sub‐regions, and that as both left and right sub‐regions can contribute independently to the counts, totals can exceed 190. To draw the strongest conclusions, we focus our description on results from (i) ROI analyses with FWE or comparable simulation (AlphaSim) and small volume correction (Figure 4—R3), and (ii) whole brain results that similarly survive stringent error correction (Figure 4—W3). More detailed information on other thresholds (i.e., ROI/whole brain uncorrected and FDR corrected) is presented in Figure 4, and is available in Tables S11 and S12. Brain regions were visualized with BrainNet Viewer (Xia et al. 2013; NeuroImaging Tools and Resources Collaboratory, n.d.).

#### 3.5.1 Regions Activated—Control Samples

##### 3.5.1.1 Subcortical Regions

The amygdala was the most extensively studied brain region, specified as a ROI 162 times, of which 63 applied FWE correction and 51 subsequently reported significant activation (80.95% of contrasts). Of the 39 results from whole brain FWE corrected contrasts, the amygdala was found to be activated 39 times (100%). Other subcortical regions were less frequently specified as ROIs, and in such cases did not show activation when FWE correction was applied. Whole brain FWE corrected results also showed significant activation in the hippocampus (24 contrasts, 61.53%) and thalamus (22 contrasts, 56.41%).

##### 3.5.1.2 Frontal Regions

While frontal regions were commonly selected as ROIs, including the ventromedial prefrontal cortex (vmPFC, *n* = 17), dorsomedial prefrontal cortex (dmpPFC, *n* = 16), ventrolateral prefrontal cortex (vlPFC, *n* = 14), dorsolateral prefrontal cortex (dlPFC, *n* = 9), and the cingulate cortex (*n* = 8), none of these regions were found to be significantly activated when ROI FWE correction was applied. When whole brain activation was reported, frontal regions were more frequently found to be engaged: the vlPFC was reportedly activated 47 times (120.51% of contrasts), the dlPFC 47 times (120.51%), the dmPFC 30 times (76.92%), the vmPFC 27 times (69.23%) and cingulate cortex 31 times (79.49%).

##### 3.5.1.3 Temporal Regions

The insula and the fusiform gyrus were the most commonly investigated ROIs among the temporal regions. Out of the 22 times the insula was specified as an ROI, it was activated 5 out of 5 times when FWE correction was applied (100% of contrasts) and was reported as a peak locus of activation 20 times using whole brain FWE correction (51.28%). The fusiform gyrus, specified as an ROI 16 times, was activated in 2 studies that applied FWE correction (100%). Notably, on a whole brain level, it was significantly activated 78 times (200%). Other temporal regions were rarely explored as ROIs and were never reported to survive FWE correction. On a whole brain level, the superior and middle temporal gyrus showed significant activation 10 and 11 times (25.64% and 28.21%).

##### 3.5.1.4 Parietal Regions

Parietal regions were rarely chosen as ROIs, and if selected, did not show significant activation after FWE correction. The precuneus was the most commonly engaged area among the parietal regions, showing activation in 13 out of 39 contrasts that applied FWE correction to whole brain activation (33.33%), followed by the superior parietal gyrus (*n* = 8, 20.51%) and the inferior parietal gyrus (*n* = 8, 20.51%).

##### 3.5.1.5 Occipital Regions

Regions in the occipital lobe were not generally reported as ROIs, with the exception of the primary and secondary visual cortex which were selected as an ROI 3 and 1 times (no FWE correction applied). Activation in and surrounding the primary and secondary visual cortices was reported 36 and 58 times when whole brain activation was FWE corrected (92.31% and 148.72%). Significant whole brain activation in the lingual gyrus and the cuneus was reported 16 (41.03%) and 6 times (15.39%).

##### 3.5.1.6 Other Regions

While cerebellar regions were found to be activated in 25 contrasts of FWE corrected whole brain activation (64.10%), regions in the brain stem were less frequently reported (*n* = 4, 10.25%). Both regions were rarely selected as an ROI (cerebellum: *n* = 1; brainstem: *n* = 2) and none of these studies applied stringent FWE correction.

#### 3.5.2 Regions Activated—Patient Samples

**FIGURE 5:**
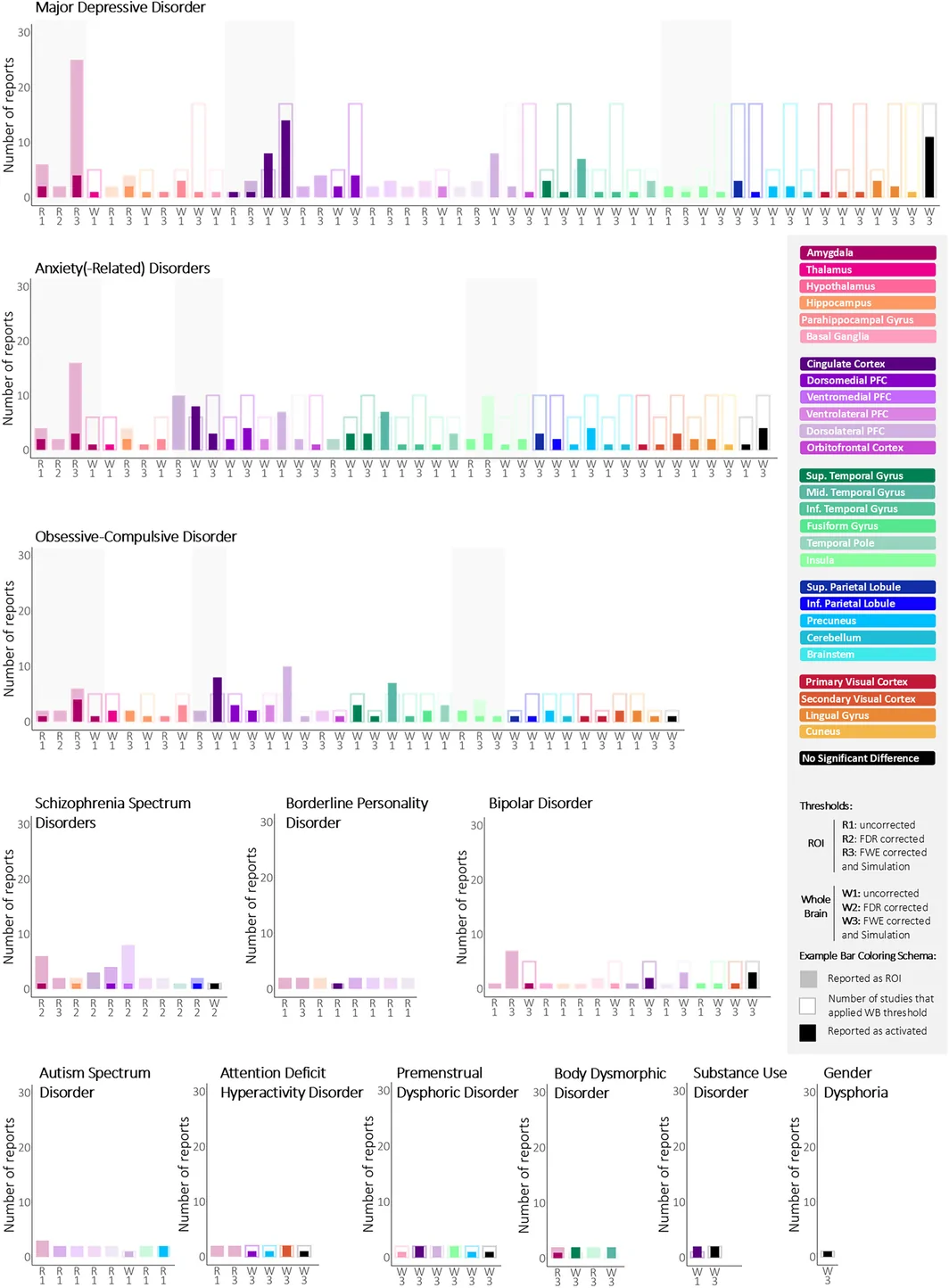
Regional search and increased activation results for the faces > shapes contrast at various statistical thresholds in patients, as compared to control participants. Bar graphs illustrate the number of times a region was reported to be searched for (lightly shaded) and/or reported as showing increased activation in the patient group compared to the control group (full color) for the faces greater than shapes contrast. For whole brain analyses, empty bars indicate how many studies applied this threshold. Results are only displayed for regions that were searched for and/or reported as active at a specific threshold. Where the number of reports of a region being activated is greater than the number of studies, this indicates that reports of subregional activation were combined within the larger region illustrated. Regions shaded in grey indicate regions of interest that are further discussed.

Once again, in the interest of brevity, we will focus on the most commonly reported regions in patients diagnosed with MDD, ARD, and OCD. See Figure 5 for an overview of regional search and increased activation results for the faces > shapes contrast at various statistical thresholds in patients, as compared to control participants, and Supporting Information 3.2.1 for a more detailed description of all activated brain regions per patient group.

The amygdala was the most frequently specified ROI for contrasts comparing activation between patient groups and controls; in total it was specified 112 times (MDD: *n* = 39; ARD: *n* = 26; OCD: *n* = 12).

Despite being frequently specified as an ROI, the amygdala exhibited significantly increased activation only 4 out of 25 times when FWE correction was applied in MDD (16.00%), 3 out of 16 times in ARD (18.75%), and 4 out of 6 times in OCD (66.67%). Interestingly, no contrasts reported increased amygdala activation when whole brain activation maps were FWE corrected in any of the three diagnostic groups. With regard to other subcortical regions, only the hippocampus and parahippocampal gyrus were sporadically selected as ROIs and showed increased activation in 1 to 4 studies across patient groups. The parahippocampal gyrus showed additional activation when investigated on a whole brain level in MDD (*n* = 1, 5.88%).

The cingulate cortex was the most frequently selected frontal ROI among the three diagnostic groups (4 times in MDD, 10 times in ARD, 2 times in OCD). However, neither the cingulate cortex nor other frontal ROIs shows increased activation in any of the patient groups. Notably, frontal regions tended to be more frequently reported as active in whole brain FWE contrasts: compared to healthy controls, the cingulate cortex showed increased activation 14 times in MDD (82.35%) and 3 times (30.00%) in ARD.

Among the temporal regions, the insula was mainly investigated as a ROI (*n* = 4 in MDD, *n* = 14 in ARD, *n* = 6 in OCD). Significantly increased activation, however, was observed only 1 out of 2 times when FWE correction was applied in MDD (50.00%), 3 out of 10 times in ARD (30.00%), and in 1 out of 4 instances in OCD (25.00%). On a whole brain level, the insula was not frequently reported to be active (*n* = 1, 5.88% in MDD; *n* = 2, 20.00% in ARD; *n* = 1, 50.00% in OCD).

Parietal, occipital, cerebellar and brainstem regions were generally not selected as ROIs. Increased activation was, however, observed occasionally in contrasts that reported FWE corrected whole brain activation, including, for example, the precuneus (*n* = 2, 11.76% in MDD; *n* = 4, 40.00% in ARD) or the superior parietal gyrus (*n* = 3, 17.65% in MDD; *n* = 3, 30.00% in ARD; *n* = 1, 50.00% in OCD).

Several studies that thresholded whole brain contrasts using stringent FWE correction did not observe significantly greater activation in the patients, relative to control groups, in any brain regions (*n* = 11, 64.70% in MDD; *n* = 4, 40.00% in ARD; *n* = 1, 50.00% in OCD).

## 4 Discussion

In this pre‐registered study, we performed a systematic review of the literature that has implemented the EFMT or one of its variants, since its broad adaptation in 2002. Our results demonstrate vast heterogeneity in all aspects of task design, naming, and implementation across studies, which limited our ability to draw firm conclusions about task activation across studies. As such, we employed a high‐level summary approach to synthesise brain regions activated during emotional face matching in healthy reference populations and psychiatric patient populations of interest. We broadly confirm the involvement of the amygdala and further identify regions that are infrequently specified as ROIs but often reported when whole brain analyses are performed. Moreover, we show that an increase in amygdala activation, as compared to controls, is inconsistent both within and across mental health conditions, and instead highlight transdiagnostic alterations in cingulate cortex activation during emotional face matching.

Considering only five task design parameters (target stimuli, facial expressions, instructions, control stimuli, other stimuli) there were 130 unique versions of the EFMT in the 183 samples included in this review. Prior studies within the broader field of face processing have shown that changes in stimuli (Müller et al. 2018), instructions (Geissberger et al. 2020; Müller et al. 2018; Cohen Kadosh et al. 2010), expressions (Jamieson et al. 2021), and task length (Lois et al. 2018), have discernible effects on the cognitive process being studied, including differential involvement of subregions of the fusiform, the inferior frontal junction, the middle temporal gyrus, the left fusiform, the right insula and the dorsolateral prefrontal cortex (Geissberger et al. 2020; Müller et al. 2018; Cohen Kadosh et al. 2010). Besides the non‐systematic modification of tasks included in this review, experimenter degrees of freedom regarding acquisition parameters and the preprocessing of fMRI task data introduce an additional source of variance known to alter task‐evoked brain activation identified (Botvinik‐Nezer et al. 2020). As such, there is simply too much heterogeneity to draw strong conclusions about the influence of these changes.

We also note that some studies included in this review may have involved partially overlapping samples. Although it was possible in some cases to identify publications analysing the same dataset (e.g., six manuscripts including potentially overlapping samples from the Duke Neurogenetics Study), it was not feasible to determine sample overlap retrospectively for the majority of studies. Consequently, each sample was treated as independent for the purposes of this review, although we acknowledge that this may have introduced some bias.

### 4.1 Regions Activated

#### 4.1.1 Regions Activated: Controls

Our results highlight a bias of the field to perform ROI analyses that focus on the amygdala and the insula. Somewhat reassuringly given the historical application of this task, the amygdala was frequently, but not always, reported as more active in the FACES condition, both in studies that performed ROI analyses and at more stringent FWE whole brain correction. The same cannot be said for the insula which, though often searched for, was not commonly reported to be significantly active at either the ROI or whole brain level. This may be due to its more prominent role in the experience of emotion, as evidenced by literature that finds stronger insula responses to emotional stimuli when participants are asked to imitate or execute facial expressions, as opposed to simply observing them (Pohl et al. 2013).

Regions that were rarely searched for as ROIs, but which were consistently identified as more active in the faces condition when whole brain activity was considered, included (i) visual regions, (ii) expanses of the prefrontal cortex, and (iii) subcortical regions including the thalamus and hippocampus, which future studies should probe in more depth. There is increasing evidence of early affective processing in primary sensory regions when multivariate analysis methods are used (Bo et al. 2021; Savage et al. 2021), and future studies should aim to more directly investigate these regions' involvement in emotional facial processing. These findings are also in line with previous literature that supports the hypothesised involvement of the ventrolateral prefrontal cortex in general control processes during emotional face processing (Müller et al. 2018). Moreover, despite remaining relatively unexplored in this context, there is converging evidence from different imaging modalities suggesting that thalamic nuclei, particularly the LGN, RTN, and pulvinar, play a crucial role in early emotion processing (Carretié et al. 2021; McFadyen et al. 2017, 2019).

#### 4.1.2 Regions Activated: Patients

Comparable to the results obtained in healthy control samples, studies investigating differences in brain activation in response to emotional faces in clinical patient populations, compared to healthy controls, frequently employed ROI analyses in the amygdala. While increased amygdala activation was only occasionally reported across patients when specified as an ROI, it was even less frequently observed when a stringent whole brain threshold was applied. These results challenge the widely‐held notion of heightened amygdala activation as a marker of increased emotional reactivity in psychopathology.

Similar results were obtained for the insula and frontal regions which, despite being commonly selected as ROIs, rarely exhibited increased activation in patient groups. However, frontal areas tended to exhibit more consistently heightened activation in patients in whole brain analyses. We would like to particularly emphasise the cingulate cortex, which consistently showed increased activation in patients with MDD, ARD, and OCD when compared to control groups. These disorders are frequently comorbid and there is considerable overlap in symptomatology, potentially stemming from shared underlying neurobiological mechanisms (Gillan et al. 2017; Hofmeijer‐Sevink et al. 2013). Different subregions of the cingulate cortex appear to serve distinct functional roles in healthy individuals and show distinct patterns of connectivity among individuals with MDD (Hiser and Koenigs 2018; Jamieson et al. 2024; Yang et al. 2020). The anterior cingulate cortex, for instance, is thought to be involved in the evaluation of emotionally valenced, specifically negative stimuli, as well as in the consolidation and retrieval of emotional memories (Palomero‐Gallagher and Amunts 2022), while the posterior area is further implicated in the cognitive control of motor responses to emotional stimuli (Palomero‐Gallagher and Amunts 2022; Hoffstaedter et al. 2014). While it was not possible to reliably distinguish between different subregions in this review, its increased activation across patient groups and task variations might indicate a general shift in encoding and processing of valenced stimuli, warranting further exploration of the function of the cingulate gyrus and its subregions in the context of this task.

### 4.2 Challenges

#### 4.2.1 Task Variability

The large number of unique modifications to the original EFMT design raises concerns for the validity of new variants to test the construct originally targeted. Each modification could alter the mental process engaged and thus drawing conclusions, especially claiming successful replication, based on previous research that likely used different versions of the task is potentially unfounded. We do not doubt that researchers have made their modifications with informed purpose and we are by no means suggesting that these edits should not have been made. Without thoroughly justifying modifications and quantifying their impact, however, the newly generated information cannot be appropriately integrated into our understanding of the ‘process’ at hand: at what point would we realise we are capturing a new phenomenon entirely?

#### 4.2.2 Reported Activation

With our study and others revealing the potentially huge influence of small design choices (Lois et al. 2018; Savage et al. 2024), it was near impossible and would have been uninformative to try to infer the relation of these design changes to task activation, as was our original aim. Furthermore summarising task activation was a major challenge due to the inconsistent naming conventions applied, with some studies choosing anatomical naming conventions to define activations (e.g., frontal superior medial gyrus) and others using functional conventions (e.g., visual cortex). While both have their merits for specificity and interpretation, without further details such as specific coordinates or group level maps, our ability to draw conclusions across studies is limited.

The frequent use of ROI analyses focused on the amygdala and insula is a bias in the literature that likely limits the depth of our understanding and overlooks the fundamental interconnectedness of brain structures and the complexity of even the most simple functional processes. This bias is exacerbated in other fields of cognitive neuroscience, such as in studies of fear conditioning, where the absence of amygdala activation to threat cues can be an exclusion criterion for participants (Fullana et al. 2018); we cannot exclude the possibility of such a bias in emotional face matching tasks as these participants or studies would likely not reach publication. Furthermore, given that some regions appear to be more consistently identified through whole‐brain analyses than ROI‐based approaches, it is possible that studies adopting a WB approach differed in methodological quality or statistical power. While these factors are difficult to evaluate within this review, their potential influence warrants further investigation.

It is also important to consider that the activation summaries we report do not consider ongoing discussion about the within‐subject test–retest validity of this task (Plichta et al. 2012; Nord et al. 2017; Elliott et al. 2020), potentially introducing additional variance in observed task activation. Using the normative modelling framework (explained below) may prove a suitable alternative to assessing test–retest using individual activation maps, as the derived deviation scores reportedly capture stable individual differences in task evoked activation (Savage et al. 2024). Lastly, due to the large number of reported contrasts across studies, we focused our synthesis on the primary contrasts (i.e., faces vs. shapes and patient vs. control comparisons), as these were most consistently examined across studies. Other contrasts, such as a comparison of selected facial expressions (e.g., angry > neutral) were not synthesised in the present review but are documented in the accompanying data extraction table and could be explored in future studies.

### 4.3 Recommendations

During data extraction, it became apparent that reporting of the experimental design was often incomplete, with many studies omitting information recommended by fMRI reporting guidelines such as those proposed by Poldrack et al. (2008) and Guo et al. (2014). Given the aforementioned limitations to interpretation this lack of information imposes, there should be an impetus to explicitly detail the parameters selected in future studies. We hope that clearer reporting will be applied in the future to allow exact replications or careful manipulations, and aid the interpretation of independent study results.

One solution to constrain this heterogeneity is to define the ‘standard’ EFMT task, as has been attempted in other domains of cognitive neuroscience (Ferguson et al. 2008; Bach et al. 2023). In line with this, alongside this manuscript we publish freely downloadable PsychoPy (Peirce 2007) code for a standardised EFMT (available at https://github.com/Hannah‐Savage/Standard_EFMT), with parameters chosen to match those found to be the most commonly implemented in this review.^2^ Importantly, this standardised version is not intended to imply that these parameters necessarily produce the most robust or reliable effects. Rather, it provides a common reference point that can facilitate transparent reporting of changes made to the standard design and enable systematic evaluation of how specific task design choices influence outcome measures. We therefore encourage researchers to implement our standard version if using the EFMT in their future studies, and/or objectively compare the effect of their modifications to the standardised EFMT we provide. If studies do still choose to implement an alternative or edited version of this EFMT we encourage the authors to use open source software (such as PsychoPy) and to upload all files required to run the task on open science platforms. This will enable other researchers to easily and freely access the specific task used in a study and allow for direct replication efforts. In line with this, we thank the Laureate Institute for Brain Research whose code we edited to generate the standard EFMT that we publish alongside this article.

Another alternative to address the challenge of heterogeneity embraces recent methodological advances such as the normative modeling framework that facilitates the aggregation of datasets that use distinct versions of a task to a common reference model. Provided that datasets are transparently and completely reported, this enables meaningful comparisons to be drawn across studies and affords the opportunity to quantitatively determine the influence of different task parameters and sample characteristics on functional activity across cohorts; this approach was recently applied to combine data from 6 independent sites, each with unique combinations of EMFT design and acquisition parameters (Savage et al. 2024).

We encourage future studies to include the results of a thresholded (FDR or FWE) whole brain analysis to remove any potential bias that may be induced when using ROI analyses alone, and to report a table of coordinates, including specific anatomical labelling, x, y, z coordinates and cluster statistics. Also, we encourage authors to upload group maps to open access data sharing platforms allowing for an objective interpretation and quantification of results across the literature (Nichols et al. 2017). Lastly, in line with this, we believe that future meta‐analyses could build on our findings by providing a more detailed understanding of how differences in task design influence brain activation during emotional face processing.

## 5 Conclusions

Our systematic review demonstrates that the literature on a task believed to be simple is severely complicated by heterogeneity in its implementation and nomenclature, in almost all aspects of design and analysis. Our high level synthesis of EFMT‐evoked activation generally confirms the involvement of the amygdala during this process in control participants but provides insufficient evidence to suggest activation is reliably altered within or across mental health conditions. We provide two actionable solutions which address or embrace this heterogeneity, respectively; first, we introduce a standardised version of the EFMT, and second, we present a method which enables the aggregation of distinct datasets to a common reference space. We echo prior calls for authors to report all necessary methodological information about the population, task and acquisition parameters applied in a study, and to provide fully transparent and comprehensive results where possible. Undoubtedly, these challenges plague other domains of cognitive and affective science, and we hope the lessons learnt from this review will be applied elsewhere. By collaboratively reinitiating our efforts to study emotional face processing in a systematic manner, we can embrace and harness the heterogeneity in our research to better understand this fundamental process.

## Funding

This work was supported by the ERC Consolidator Grant (101001118).

## Conflicts of Interest

The authors declare no conflicts of interest.

## Supporting information


**Figure S1:** PRISMA flow diagram. The flow diagram illustrates the flow of information through the different phases of the systematic review. It maps out the number of records identified, included and excluded, and the reasons for exclusions. Titles and abstracts were screened and full texts were assessed for eligibility. *Manuscript inaccessible, and corresponding author did not respond to request to provide a copy of the published manuscript. **3 of these added manuscripts were excluded during initial identification as the task description seemed irrelevant from the abstract.
**Figure S2:** Terminology example: A single article with two samples/studies (A, B), where study A has 2 groups (A.1 and A.2). Participants in group A.1 are scanned using two different scanners (S1 and S2). Scanner 1 used two different settings for their scanning parameters (AP1 and AP2) over the course of the study. All data in this manuscript is preprocessed in the same way. The manuscript reports significant activations for the face > shapes contrast for group A.1 and A.2 alone, as well as for Sample A > Sample B.
**Figure S3:** Legend for Figure 3b: Task stimuli and instructions: Each row of dots represents the stimuli used in a task (total *n* = 184). For each task parameter (column) a change in colour indicates a different stimuli from the original task design. Note there is no inherent ordering to the colours, rather they reflect different categorical variables. The corresponding colour for each stimulus or instruction is detailed below.
**Figure S4:** Heterogeneity of scanners, acquisition parameters and preprocessing software used in included studies. (a) Scanner type. In the 184 scans reported 32 unique scanners used. The most frequently used MRI models are highlighted with their respective percentage as a proportion of the number of scans (*n* = 184 (+NA). (b) Acquisition parameters. Each bar illustrates the relative proportion of scanning parameters (total *n* = 184) with the respective value of the different settings: Time‐to‐Echo (TE), Repetition Time (TR), Flip angle (°), Field of view (FOV; mm), Matrix size (mm2). The width of each stream between the bars represents the total number of samples with those parameters. (c) Preprocessing software used. In the 183 included manuscripts, there were 31 different pre‐processing softwares used. The most frequently used software versions are highlighted with their respective percentage as a proportion of the number of pre‐processing softwares.

**Table S1:** Included manuscripts.
**Table S2:** Synthesized task names.
**Table S3a:–f** Synthesized brain region labels.
**Table S4:** Frequency of task names.
**Table S5a:** Frequency of face stimuli.
**Table S5b:** Frequency of facial expressions.
**Table S6a:** Frequency of primary task instructions.
**Table S7:** Frequency of control stimuli.
**Table S8:** Frequency of other stimuli.
**Table S9a:** Number of face blocks.
**Table S10a:** Face stimuli—total number of trials.
**Table S11:** Statistical thresholds applied for the faces > shapes contrast in healthy controls.
**Table S12a–l:** Statistical thresholds applied for the patients > controls: faces > shapes contrast.

**Supplementary Information:** hbm70636‐sup‐0003‐Supinfo.docx

## Data Availability

The data extraction table and all code to run the analysis and generate the plots in the manuscript can be found at https://github.com/predictive‐clinical‐neuroscience/EFMT_Review.

## Endnotes

^1^
A note on the extraction of sex and gender: We acknowledge that sex and gender are not synonymous, and recognise that research and reporting should align with recognised guidelines such as the SAGER guidelines (Heidari et al. 2016) in order to adequately determine their independent relation to health. For the purpose of this review, we conferred that the definition and general use of these terms and constructs have evolved since 2002. As such, we did not feel that there was sufficient clarity to determine which of these two attributes was explicitly, or rather implicitly, recorded in past studies and chose to summarise them together.

^2^
We make an exception that this task uses facial stimuli from the NimStim Set of Facial Expressions as, compared to the Ekman Series, this stimulus set is freely available.
