# Supplementary material for: Do You Match What I Match? A Systematic Review of Over 20 Years of the Emotional Face Matching Task Reveals Vast Inconsistencies in Design and Implementation
Source: Hum Brain Mapp. 2026 Sep 21;47(14):e70636. doi: 10.1002/hbm.70636 (PMC13594551; doi:10.1002/hbm.70636)
Supplement: Supplementary file 1 [file HBM-47-e70636-s001.docx]

**Supplementary Tables**

**Supplementary Table 1: Included Manuscripts**

| **Article** | **Authors** | **Year** | **Title** |
| --- | --- | --- | --- |
| 1 | Hariri et al. | 2000 | Modulating emotional responses: effects of a neocortical network on the limbic system |
| 2 | Hariri et al. | 2002 | Serotonin Transporter Genetic Variation and the Response of the Human Amygdala |
| 3 | Hariri et al. | 2002 | The Amygdala Response to Emotional Stimuli: A Comparison of Faces and Scenes |
| 4 | Quintana et al. | 2003 | Right lateral fusiform gyrus dysfunction during facial information processing in schizophrenia |
| 5 | Wang et al. | 2004 | Neural correlates of facial affect processing in children and adolescents with autism spectrum disorder. |
| 6 | Piggot et al. | 2004 | Emotional attribution in high-functioning individuals with autistic spectrum disorder: A functional imaging study |
| 7 | Tessitore et al. | 2005 | Functional changes in the activity of brain regions underlying emotion processing in the elderly |
| 8 | Brown et al. | 2005 | A regulatory variant of the human tryptophan hydroxylase-2 gene biases amygdala reactivity |
| 9 | Wright & Liu | 2006 | Neutral faces activate the amygdala during identity matching |
| 10 | Feusner et al. | 2007 | Visual information processing of faces in body dysmorphic disorder |
| 11 | Scheuerecker et al. | 2007 | Cerebral differences in explicit and implicit emotional processing - An fMRI study |
| 12 | Glahn et al. | 2007 | Reduced Amygdala Activation in Young Adults at High Risk of Alcoholism: Studies from the Oklahoma Family Health Patterns Project |
| 13 | Matthews et al. | 2008 | Decreased functional coupling of the amygdala and supragenual cingulate is related to increased depression in unmedicated individuals with current major depressive disorder |
| 14 | Fakra et al. | 2008 | Neural bases of different cognitive strategies for facial affect processing in schizophrenia |
| 15 | Payer et al. | 2008 | Differences in cortical activity between methamphetamine-dependent and healthy individuals performing a facial affect matching task |
| 16 | Meyer-Lindenberg et al. | 2009 | Genetic variants in AVPR1A linked to autism predict amygdala activation and personality traits in healthy humans. |
| 17 | Delaveau et al. | 2009 | Dopaminergic modulation of amygdala activity during emotion recognition in patients with parkinson disease |
| 18 | Pujol et al. | 2009 | Influence of the fusiform gyrus on amygdala response to emotional faces in the non-clinical range of social anxiety |
| 19 | Peluso et al. | 2009 | Amygdala hyperactivation in untreated depressed individuals |
| 20 | Salgado-Pineda et al. | 2010 | Differential patterns of initial and sustained responses in amygdala and cortical regions to emotional stimuli in schizophrenia patients and healthy participants. |
| 21 | Labuschagne et al. | 2010 | Oxytocin attenuates amygdala reactivity to fear in generalized social anxiety disorder |
| 22 | Kleinhans et al. | 2010 | Association between amygdala response to emotional faces and social anxiety in autism spectrum disorders |
| 23 | Scheuerecker et al. | 2010 | Orbitofrontal volume reductions during emotion recognition in patients with major depression |
| 24 | Yang et al. | 2010 | Adolescents With Major Depression Demonstrate Increased Amygdala Activation |
| 25 | Moses-Kolko et al. | 2010 | Abnormally reduced dorsomedial prefrontal cortical activity and effective connectivity with amygdala in response to negative emotional faces in postpartum depression |
| 26 | Zink et al. | 2010 | Vasopressin modulates medial prefrontal cortex-amygdala circuitry during emotion processing in humans |
| 27 | Windischberger et al. | 2010 | Area-specific modulation of neural activation comparing escitalopram and citalopram revealed by pharmaco-fMRI: A randomized cross-over study |
| 28 | Cardoner et al. | 2011 | Enhanced brain responsiveness during active emotional face processing in obsessive compulsive disorder. |
| 29 | Quintana et al. | 2011 | Brain dysfunctions during facial discrimination in schizophrenia: selective association to affect decoding. |
| 30 | Hahn et al. | 2011 | Reduced resting-state functional connectivity between amygdala and orbitofrontal cortex in social anxiety disorder. |
| 31 | Frodl et al. | 2011 | Different effects of mirtazapine and venlafaxine on brain activation: an open randomized controlled fMRI study. |
| 32 | Padula et al. | 2011 | Alcohol attenuates activation in the bilateral anterior insula during an emotional processing task: a pilot study. |
| 33 | El et al. | 2011 | Amygdala activity correlates with attentional bias in PTSD |
| 34 | van Wingen et al. | 2011 | Neural basis of emotion recognition deficits in first-episode major depression |
| 35 | Benedetti et al. | 2011 | Emotional reactivity in chronic schizophrenia: structural and functional brain correlates and the influence of adverse childhood experiences |
| 36 | Simmons et al. | 2011 | Altered amygdala activation during face processing in Iraqi and Afghanistani war veterans |
| 37 | Zhong et al. | 2011 | Amygdala hyperactivation and prefrontal hypoactivation in subjects with cognitive vulnerability to depression |
| 38 | Hessl et al. | 2011 | Decreased fragile x mental retardation protein expression underlies amygdala dysfunction in carriers of the fragile x premutation |
| 39 | Forbes et al. | 2011 | Neural systems of threat processing in adolescents: Role of pubertal maturation and relation to measures of negative affect |
| 40 | Aizenstein et al. | 2011 | fMRI correlates of white matter hyperintensities in late-life depression |
| 41 | Smith et al. | 2012 | Storm in a coffee cup: Caffeine modifies brain activation to social signals of threat |
| 42 | Vizueta et al. | 2012 | Regional fMRI hypoactivation and altered functional connectivity during emotion processing in nonmedicated depressed patients with bipolar II disorder |
| 43 | Radaelli et al. | 2012 | Neural responses to emotional stimuli in comorbid borderline personality disorder and bipolar depression |
| 44 | Domschke et al. | 2012 | Catechol-O-methyltransferase gene variation: Impact on amygdala response to aversive stimuli |
| 45 | Klumpp et al. | 2012 | Shifting the focus of attention modulates amygdala and anterior cingulate cortex reactivity to emotional faces |
| 46 | Plichta et al. | 2012 | Test-retest reliability of evoked BOLD signals from a cognitive-emotive fMRI test battery |
| 47 | Hulvershorn et al. | 2012 | Neural activation during facial emotion processing in unmedicated bipolar depression, euthymia, and mania |
| 48 | Sauer et al. | 2012 | Effects of a common variant in the CD38 gene on social processing in an oxytocin challenge study: Possible links to autism |
| 49 | Hulvershorn et al. | 2013 | Cortical activation deficits during facial emotion processing in youth at high risk for the development of substance use disorders. |
| 50 | Gaebler et al. | 2013 | Heart rate variability and its neural correlates during emotional face processing in social anxiety disorder |
| 51 | Marusak et al. | 2013 | The stimuli drive the response: An fMRI study of youth processing adult or child emotional face stimuli |
| 52 | Tesli et al. | 2013 | CACNA1C Risk Variant and Amygdala Activity in Bipolar Disorder, Schizophrenia and Healthy Controls |
| 53 | Sauder et al. | 2013 | Test-retest reliability of amygdala response to emotional faces |
| 54 | Labus et al. | 2013 | Sex differences in emotion-related cognitive processes in irritable bowel syndrome and healthy control subjects |
| 55 | Gorka et al. | 2013 | Basal forebrain moderates the magnitude of task-dependent amygdala functional connectivity |
| 56 | Passamonti et al. | 2013 | Dysfunctions within limbic-motor networks in amyotrophic lateral sclerosis |
| 57 | Barch et al. | 2013 | Function in the human connectome: Task-fMRI and individual differences in behavior |
| 58 | Prather et al. | 2013 | Impact of sleep quality on amygdala reactivity, negative affect, and perceived stress |
| 59 | Bastiaansen et al. | 2014 | Filling the gap: relationship between the serotonin-transporter-linked polymorphic region and amygdala activation. |
| 60 | Fonzo et al. | 2014 | Cognitive-behavioral therapy for generalized anxiety disorder is associated with attenuation of limbic activation to threat-related facial emotions |
| 61 | Swartz et al. | 2014 | Dynamic changes in amygdala activation and functional connectivity in children and adolescents with anxiety disorders |
| 62 | Swartz et al. | 2014 | Altered activation of the rostral anterior cingulate cortex in the context of emotional face distractors in children and adolescents with anxiety disorders |
| 63 | Ferri et al. | 2014 | The impact of puberty and social anxiety on amygdala activation to faces in adolescence |
| 64 | Bangen et al. | 2014 | Brains of optimistic older adults respond less to fearful faces |
| 65 | Via et al. | 2014 | Amygdala activation and symptom dimensions in obsessive-compulsive disorder |
| 66 | Lindsay et al. | 2014 | Amygdala Activation and Emotional Processing in Adolescents at Risk for Substance Use Disorders |
| 67 | Dannlowski et al. | 2015 | Multimodal imaging of a tescalcin (TESC)-regulating polymorphism (rs7294919)-specific effects on hippocampal gray matter structure. |
| 68 | Tesli et al. | 2015 | Altered brain activation during emotional face processing in relation to both diagnosis and polygenic risk of bipolar disorder. |
| 69 | Holz et al. | 2015 | Role of FKBP5 in emotion processing: results on amygdala activity, connectivity and volume |
| 70 | Yucel et al. | 2015 | Abnormal neural activation to faces in the parents of children with autism |
| 71 | Redlich et al. | 2015 | Evidence of an IFN-y by early life stress interaction in the regulation of amygdala reactivity to emotional stimuli |
| 72 | Marusak et al. | 2015 | Amygdala responses to salient social cues vary with oxytocin receptor genotype in youth |
| 73 | Jedd et al. | 2015 | Long-term consequences of childhood maltreatment: Altered amygdala functional connectivity |
| 74 | Rahm et al. | 2015 | Negative symptoms in schizophrenia show association with amygdala volumes and neural activation during affective processing |
| 75 | Fonzo et al. | 2015 | Common and disorder-specific neural responses to emotional faces in generalised anxiety, social anxiety and panic disorders |
| 76 | Jabbi et al. | 2015 | Variation in the Williams syndrome GTF2I gene and anxiety proneness interactively affect prefrontal cortical response to aversive stimuli |
| 77 | Burklund et al. | 2015 | Altered emotion regulation capacity in social phobia as a function of comorbidity |
| 78 | Binelli et al. | 2016 | Facial emotion processing in patients with social anxiety disorder and Williams-Beuren syndrome: an fMRI study. |
| 79 | Cao et al. | 2016 | Altered Functional Subnetwork During Emotional Face Processing: A Potential Intermediate Phenotype for Schizophrenia. |
| 80 | Holz et al. | 2016 | Evidence for a Sex-Dependent MAOAÃ— Childhood Stress Interaction in the Neural Circuitry of Aggression. |
| 81 | Kujawa et al. | 2016 | Altered Development of Amygdala-Anterior Cingulate Cortex Connectivity in Anxious Youth and Young Adults. |
| 82 | Dannlowski et al. | 2016 | Disadvantage of Social Sensitivity: Interaction of Oxytocin Receptor Genotype and Child Maltreatment on Brain Structure |
| 83 | Evans et al. | 2016 | Childhood Cumulative Risk Exposure and Adult Amygdala Volume and Function |
| 84 | Bomyea et al. | 2017 | Neural differences underlying face processing in veterans with TBI and co-occurring TBI and PTSD. |
| 85 | Opel et al. | 2017 | Prefrontal brain responsiveness to negative stimuli distinguishes familial risk for major depression from acute disorder. |
| 86 | Schreiner et al. | 2017 | Multi-modal neuroimaging of adolescents with non-suicidal self-injury: Amygdala functional connectivity |
| 87 | Baranger et al. | 2017 | An earlier time of scan is associated with greater threat-related amygdala reactivity |
| 88 | Nord et al. | 2017 | Unreliability of putative fMRI biomarkers during emotional face processing |
| 89 | MacNamara et al. | 2017 | Transdiagnostic neural correlates of affective face processing in anxiety and depression |
| 90 | Wackerhagen et al. | 2017 | Influence of Familial Risk for Depression on Cortico-Limbic Connectivity during Implicit Emotional Processing |
| 91 | Bürger et al. | 2017 | Differential Abnormal Pattern of Anterior Cingulate Gyrus Activation in Unipolar and Bipolar Depression: An fMRI and Pattern Classification Approach |
| 92 | Swartz et al. | 2017 | Threat-related amygdala activity is associated with peripheral CRP concentrations in men but not women |
| 93 | Villalta-Gil et al. | 2017 | Convergent individual differences in visual cortices, but not the amygdala across standard amygdalar fMRI probe tasks |
| 94 | Swartz et al. | 2017 | A Common Polymorphism in a Williams Syndrome Gene Predicts Amygdala Reactivity and Extraversion in Healthy Adults |
| 95 | Bottelier et al. | 2017 | Age-dependent effects of acute methylphenidate on amygdala reactivity in stimulant treatment-naive patients with Attention Deficit/Hyperactivity Disorder |
| 96 | Lois et al. | 2018 | Experimental and methodological factors affecting test-retest reliability of amygdala BOLD responses. |
| 97 | Redlich et al. | 2018 | The Limbic System in Youth Depression: Brain Structural and Functional Alterations in Adolescent In-patients with Severe Depression. |
| 98 | Bharadwaj et al. | 2018 | Genetic risk mechanisms of posttraumatic stress disorder in the human brain. |
| 99 | Schneider et al. | 2018 | Association of serotonin transporter gene AluJb methylation with major depression, amygdala responsiveness, 5-HTTLPR/rs25531 polymorphism, and stress. |
| 100 | Palmer et al. | 2018 | Avoidance of accelerated aging in schizophrenia?: Clinical and biological characterization of an exceptionally high functioning individual. |
| 101 | Sethi et al. | 2018 | Primary and Secondary Variants of Psychopathy in a Volunteer Sample Are Associated With Different Neurocognitive Mechanisms |
| 102 | Demers et al. | 2018 | Separable Effects of Childhood Maltreatment and Adult Adaptive Functioning on Amygdala Connectivity During Emotion Processing |
| 103 | Giummarra et al. | 2018 | Emotion processing in persons who respond vicariously towards others in pain: Disinhibited left-lateralized neural activity for threatening expressions |
| 104 | Cao et al. | 2018 | The 5-HTTLPR polymorphism affects network-based functional connectivity in the visual-limbic system in healthy adults |
| 105 | van der Knaap et al. | 2018 | Maternal depressive symptoms during pregnancy are associated with amygdala hyperresponsivity in children |
| 106 | Infantolino et al. | 2018 | Robust is not necessarily reliable: From within-subjects fMRI contrasts to between-subjects comparisons |
| 107 | Guell et al. | 2018 | Triple representation of language, working memory, social and emotion processing in the cerebellum: convergent evidence from task and seed-based resting-state fMRI analyses in a single large cohort |
| 108 | Piel et al. | 2018 | The effect of 5-HTTLPR and a serotonergic multi-marker score on amygdala, prefrontal and anterior cingulate cortex reactivity and habituation in a large, healthy fMRI cohort |
| 109 | Schmitt et al. | 2019 | Effects of low- and high-intensity exercise on emotional face processing: an fMRI face-matching study. |
| 110 | Ren et al. | 2019 | Brain Functional Basis of Subjective Well-being During Negative Facial Emotion Processing Task-Based fMRI. |
| 111 | Peters et al. | 2019 | The roles of early-life adversity and rumination in neural response to emotional faces amongst anxious and depressed adults. |
| 112 | Dan et al. | 2019 | Impact of dopamine and cognitive impairment on neural reactivity to facial emotion in Parkinson's disease. |
| 113 | Swartz et al. | 2019 | Amygdala activity to angry and fearful faces relates to bullying and victimization in adolescents |
| 114 | Li et al. | 2019 | Abnormal brain activation during emotion processing of euthymic bipolar patients taking different mood stabilizers |
| 115 | Wackerhagen et al. | 2019 | Amygdala functional connectivity in major depression-disentangling markers of pathology, risk and resilience |
| 116 | Preckel et al. | 2019 | Neural mechanisms of affective matching across faces and scenes |
| 117 | Zhu et al. | 2019 | Association of Prepubertal and Postpubertal Exposure to Childhood Maltreatment with Adult Amygdala Function |
| 118 | Geissberger et al. | 2020 | Reproducibility of amygdala activation in facial emotion processing at 7T. |
| 119 | Enneking et al. | 2020 | Brain functional effects of electroconvulsive therapy during emotional processing in major depressive disorder. |
| 120 | Kreuder et al. | 2020 | Common and dissociable effects of oxytocin and lorazepam on the neurocircuitry of fear. |
| 121 | Loureiro et al. | 2020 | Modulation of amygdala reactivity following rapidly acting interventions for major depression. |
| 122 | Aggensteiner et al. | 2020 | The effects of callous-unemotional traits and aggression subtypes on amygdala activity in response to negative faces. |
| 123 | Beking et al. | 2020 | Testosterone effects on functional amygdala lateralization: A study in adolescent transgender boys and cisgender boys and girls. |
| 124 | Wong et al. | 2020 | Serotonin differentially modulates the temporal dynamics of the limbic response to facial emotions in male adults with and without autism spectrum disorder (ASD): a randomised placebo-controlled single-dose crossover trial |
| 125 | Herrmann et al. | 2020 | fMRI Revealed Reduced Amygdala Activation after Nx4 in Mildly to Moderately Stressed Healthy Volunteers in a Randomized, Placebo-Controlled, Cross-Over Trial |
| 126 | Pozzi et al. | 2020 | The Influence of Maternal Parenting Style on the Neural Correlates of Emotion Processing in Children |
| 127 | Murphy et al. | 2020 | Left, right, or bilateral amygdala activation? How effects of smoothing and motion correction on ultra-high field, high-resolution functional magnetic resonance imaging (fMRI) data alter inferences |
| 128 | Harada et al. | 2020 | Cultural influences on neural systems of intergroup emotion perception: An fMRI study |
| 129 | Viering et al. | 2021 | Amygdala reactivity and ventromedial prefrontal cortex coupling in the processing of emotional face stimuli in attention-deficit/hyperactivity disorder. |
| 130 | Jamieson et al. | 2021 | Differential Modulation of Effective Connectivity in the Brain's Extended Face Processing System by Fearful and Sad Facial Expressions. |
| 131 | Sladky et al. | 2021 | Dynamic Causal Modeling of the Prefrontal/Amygdala Network During Processing of Emotional Faces. |
| 132 | Morr et al. | 2021 | Insula reactivity mediates subjective isolation stress in alexithymia. |
| 133 | Daedelow et al. | 2021 | Neural correlates of RDoC-specific cognitive processes in a high-functional autistic patient: a statistically validated case report |
| 134 | Jamieson et al. | 2021 | Altered effective connectivity of the extended face processing system in depression and its association with treatment response: Findings from the YoDA-C randomized controlled trial |
| 135 | Neukel et al. | 2021 | A mechanism-based approach to anti-aggression psychotherapy in borderline personality disorder: Group treatment affects amygdala activation and connectivity |
| 136 | Nagy et al. | 2021 | Childhood maltreatment results in altered deactivation of reward processing circuits in depressed patients: A functional magnetic resonance imaging study of a facial emotion recognition task |
| 137 | Kennedy et al. | 2021 | Elucidating the neural correlates of emotion recognition in children with sub-clinical anxiety |
| 138 | Liu et al. | 2021 | Oxytocinergic Modulation of Threat-Specific Amygdala Sensitization in Humans Is Critically Mediated by Serotonergic Mechanisms |
| 139 | Quidé et al. | 2021 | The relationship between cortisol reactivity and emotional brain function is differently moderated by childhood trauma, in bipolar disorder, schizophrenia and healthy individuals |
| 140 | Böhnlein et al. | 2021 | Neural processing of emotional facial stimuli in specific phobia: An fMRI study |
| 141 | Yoon et al. | 2021 | Reward- and threat-related neural function associated with risk and presence of depression in adolescents: a study using a composite risk score in Brazil |
| 142 | Lemke et al. | 2021 | The Course of Disease in Major Depressive Disorder Is Associated With Altered Activity of the Limbic System During Negative Emotion Processing |
| 143 | Heunis et al. | 2021 | rt-me-fMRI: a task and resting state dataset for real-time, multi-echo fMRI methods development and validation |
| 144 | Yang et al. | 2007 | Increased amygdala activation is related to heart rate during emotion processing in adolescent subjects. |
| 145 | Rasetti et al. | 2009 | Evidence That Altered Amygdala Activity in Schizophrenia is Related to Clinical State and Not Genetic Risk |
| 146 | Stein et al. | 2007 | Increased amygdala and insula activation during emotion processing in anxiety-prone subjects. |
| 147 | Bertolino et al. | 2005 | Variation of Human Amygdala Response During Threatening Stimuli as a Function of 5′HTTLPR Genotype and Personality Style |
| 148 | Altshuler et al. | 2005 | Increased Amygdala Activation During Mania: A Functional Magnetic Resonance Imaging Study |
| 149 | Zhou et al. | 2008 | Genetic variation in human NPY expression affects stress response and emotion |
| 150 | Meyer-Lindenberg et al. | 2005 | Neural correlates of genetically abnormal social cognition in Williams syndrome |
| 151 | Klumpp et al. | 2013 | Anterior cingulate cortex and insula response during indirect and direct processing of emotional faces in generalized social anxiety disorder. |
| 152 | Dannlowski et al. | 2011 | Neuropeptide-S (NPS) Receptor Genotype Modulates Basolateral Amygdala Responsiveness to Aversive Stimuli |
| 153 | Boubela et al. | 2017 | fMRI measurements of amygdala activation are confounded by stimulus correlated signal fluctuation in nearby veins draining distant brain regions |
| 154 | Borgers et al. | 2022 | Brain functional correlates of emotional face processing in body dysmorphic disorder |
| 155 | Duyser et al. | 2022 | Amygdala sensitivity for negative information as a neural marker for negative memory bias across psychiatric diagnoses |
| 156 | Enneking et al. | 2022 | Changes in brain function during negative emotion processing in the long-term course of depression |
| 157 | Fisher et al. | 2022 | Emotional faces processing in major depressive disorder and prediction of antidepressant treatment response: A NeuroPharm study |
| 158 | Procyshyn et al. | 2022 | Oxytocin enhances basolateral amygdala activation and functional connectivity while processing emotional faces: preliminary findings in autistic vs non-autistic women |
| 159 | Swartz et al. | 2022 | Associations between peripheral inflammatory markers and amygdala activity and connectivity in response to emotional faces in adolescents |
| 160 | Aratò et al. | 2023 | Emotional face expression recognition in problematic Internet use and excessive smartphone use: task-based fMRI study |
| 161 | Boucherie et al. | 2023 | Modulation of functional networks related to the serotonin neurotransmitter system by citalopram: Evidence from a multimodal neuroimaging study |
| 162 | Deng et al. | 2023 | Reactivity of the ventromedial prefrontal cortex, but not the amygdala, to negative emotion faces predicts greed personality trait |
| 163 | Labuschagne et al. | 2023 | Specialization of amygdala subregions in emotion processing |
| 164 | Meyer et al. | 2023 | Daring to Feel: Emotion-Focused Psychotherapy Increases Amygdala Activation and Connectivity in Euthymic Bipolar Disorder—A Randomized Controlled Trial |
| 165 | Stiernman et al. | 2023 | Emotion-induced brain activation across the menstrual cycle in individuals with premenstrual dysphoric disorder and associations to serum levels of progesterone-derived neurosteroids |
| 166 | Sun et al. | 2023 | Free-viewing gaze patterns reveal a mood-congruency bias in MDD during an affective fMRI/eye-tracking task |
| 167 | Zhu et al. | 2023 | Functional Connectivity Between Basal Forebrain and Superficial Amygdala Negatively Correlates with Social Fearfulness |
| 168 | Armand et al. | 2024 | Functional brain responses to emotional faces after three to five weeks of intake of escitalopram in healthy individuals: a double-blind, placebo-controlled randomised study |
| 169 | Armand et al. | 2024 | Amygdala response to emotional faces following acute administration of psilocybin in healthy individuals |
| 170 | Erdmann et al. | 2024 | Amygdala Reactivity, Antidepressant Discontinuation, and Relapse |
| 171 | Langenbach et al. | 2024 | Autistic and non-autistic individuals show the same amygdala activity during emotional face processing |
| 172 | Li et al. | 2024 | Happy people are always similar: The evidence from brain morphological and functional inter-subject correlations |
| 173 | Sheng et al. | 2024 | Altered effective connectivity among face-processing systems in major depressive disorder |
| 174 | van der Straten et al. | 2024 | Pharmacological and Psychological Treatment Have Common and Specific Effects on Brain Activity in Obsessive-Compulsive Disorder |
| 175 | Borgers et al. | 2025 | Changes in brain function during negative emotion processing following cognitive–behavioural therapy in depressive disorders |
| 176 | Chen et al. | 2025 | Setting the tone for the day: Cortisol awakening response proactively modulates fronto-limbic circuitry for emotion processing |
| 177 | Chen et al. | 2025 | The effects of polygenic risks for alcohol misuse on negative emotion processing in young adult binge drinkers |
| 178 | Chung et al. | 2025 | The role of amygdala reactivity in affective fluctuations across social contexts |
| 179 | Díaz et al. | 2025 | Neural Substrates of Emotion Processing and Cognitive Control Over Emotion in Youth Anxiety: An RDoC-Informed Study Across the Clinical to Nonclinical Continuum of Severity |
| 180 | Hammond et al. | 2025 | Altered effective connectivity of emotion perception and regulation networks during an emotional face perception task in adults with alcohol use disorder |
| 181 | Hwang et al. | 2025 | Amygdala-Centered Emotional Processing in Prolonged Grief Disorder: Relationship With Clinical Symptomatology |
| 182 | Jeon et al. | 2026 | Intolerance of uncertainty modulates amygdala habituation to emotional faces |

**Supplementary Table 2: Synthesized Task Names**

| **Standardised name given to task** | **Original name given to task** |
| --- | --- |
| [Faces and shapes] Affective reactivity task | [Faces And Shapes] Affective Reactivity Task |
| Affect-labeling face-matching blocked paradigm (Chai et al. 2015) | Affect Labelling Face Matching Task |
| Affective Face Matching Task | Affective Face Matching Task |
| Amygdala Reactivity Paradigm | Amygdala Reactivity Paradigm |
| Aversive Face-Viewing Task | Aversive Face-Viewing Task |
| Behavioral fMRI Imaging Paradigm | Behavioral Fmri Imaging Paradigm |
| Cortico-limbic reactivity task | Cortico-Limbic Reactivity Task |
| Emotion Discrimination Task | Emotion Discrimination Task |
| Emotion Emotion Processing Task; Emotion(al) Face Face Matching Task | Emotion Emotion-Processing Task/Emotional Face Face-Matching Task |
| Emotion Face Assessment Task | Emotion Face Assessment Task |
| Emotion Matching Task | Emotion-Labelling; Emotion-Matching And A Baseline Control Task |
|  | Emotion-Matching Task |
|  | Emotion Matching Task |
| Emotion Processing Task | Emotion Processing Task |
| Emotion Recognition Task | Emotion Recognition Paradigm |
| Emotion(al) Face Assessment Task | Emotional Faces Assessment Task (Efat) |
| Emotion(al) Face Assessment Task - Modified | A Modified Version Of The Emotional Face Assessment Task |
|  | A Slightly Modified Version Of The Hariri Emotion Face Assessment Task (Hariri Et Al. 2000); Face-Processing Task |
|  | Modified Emotion Face Assessment Task |
| Emotion(al) Face Match(ing)/Label(ling) Task | Emotion Match (Perceptual) Task AND Emotion Label (Linguistic) Task |
| Emotion(al) Face Matching Task | Emotional Face Matching Task |
|  | Emotional Facial Matching Test |
|  | Emotion Face-Matching Task |
|  | Emotional Face-Matching Task |
|  | Emotional Face-Matching Block-Design Task |
|  | Emotional Face Matching Challenge Paradigm |
| Emotion(al) Face Matching Task - Modified | A Modified Version Of The Emotional Face Matching Task (EMFT) |
|  | Modified Emotional Face Matching Task |
|  | Modified Emotional Face Matching Task/ Perceptual Face Processing Paradigm |
| Emotion(al) Face Processing Task | Emotional Face Processing Task |
|  | Emotional Face Processing Task; Perceptual Face Processing Paradigm |
| Emotion(al) Task | Emotion Task |
|  | Emotional Paradigm |
|  | Emotional Task - Sensorimotor Task |
| Emotional Face(s) Task | Emotional Faces Paradigm |
| Emotional Faces Shifting Attention Task | Emotional Faces Shifting Attention Task |
| Emotional Faces Task | Emotional Faces Task |
| Emotional Reactivity Task | Emotional Reactivity Task |
| Emotive Face Identification Task | Emotive Face Identification Task |
| Explicit/Implicit Processing Task | Explicit/Implicit Processing Task |
| Face Identification task | Face Identification Task |
| Face Match(ing) Task | Fmri Face-Matching Task |
|  | Face-Matching Paradigm |
|  | Face-Matching Task |
|  | Face Matching Paradigm |
|  | Face Matching Task |
|  | Faces Matching Task |
| Face Match(ing) Task - Modified | A Frequently Used Face-Matching Fmri Paradigm (Redlichet Al 2015, 2015b) Has Been Used To Investigate Emotion Processing |
|  | Extended version of the Hariri face matching task |
| Face Processing Task | Face-Processing Fmri Paradigm |
|  | Face Processing Fmri Paradigm |
| Faces Task | Faces Task |
| Facial Affect Matching Task | Facial Affect-Matching Task |
| Facial Emotion Matching Task | Facial-Emotion Matching Task |
| Facial Emotion Processing Task | Facial Emotion Processing Task; Hariri Task |
|  | In-Scanner Facial Emotionprocessing Task |
|  | Facial Information Discrimination Task |
| fMRI Task | Fmri Paradigm |
| Hariri and colleagues (2002) | Experimental Paradigm Developed By Hariri Et Al (2000) |
| Hariri and colleagues (2002) - Modified | A Modified Version Of The Frequently Used Paradigm Described By Hariri And Colleagues (2002); Facial Expression Discr… |
|  | Emotional Face Matching Task Adapted From Hariri Et Al. |
|  | Extended Version Of The Hariri Face Matching Task |
| Implicit Emotion Processing Task | Implicit Emotional Processing Task |
| Implicit Emotion Recognition Task | Implicit Emotion Recognition Task |
| Implicit Emotion(al) Face Matching Task | Implicit Emotional Face Matching Task |
| Implicit Facial Information Processing Task | Implicit Facial Information Processing Task |
| Matching Faces Judgment | Matching-Faces Judgment And Matching-Shapes Judgment Task |
| Matching Task | Matching Task |
| Negative Emotional Face Processing | Negative Emotional Face-Processing Paradigm |
| NimStim Emotional Faces Viewing Task | Nimstim Emo-Tional Faces Viewing Task/ Faces Paradigm |
| Perceptual Face Processing Task | Perceptual Face Processing Paradigm |
| Robust paradigm for eliciting [amygdala/subcortical] [responsiveness/activation] - Modified | A Version Of A Task Known To Evoke Robust Responses In Limbic Regions Including The Amygdala And PFC |
| Robust paradigm for eliciting [amygdala/subcortical] [responsiveness/activation] used previously | A Robust Paradigm For Eliciting Amygdala Responsiveness That Has Been Used In Several Previous Imaging Studies |
|  | A Robust Paradigm For Eliciting Subcortical Especially Amygdala Activation Using Emotional Faces That Has Been Used |
|  | An Fmri Paradigm That Has Repeatedly Been Shown To Robustly Elicit Neural Responsiveness During Pro-Cessing Of Emotion |
|  | The Experimental Paradigm Consisted Of A Blocked Design And Has Been Previously Used To Assess Drug Effects On Amygdala |
| Simultaneous Identify Matching Task | Simultaneous Identify Matching |
| Simultaneous Affect Matching/Labelling Task | Simultaneous Affect Matching Or Labelling |
| Simultaneous Facial Information Matching Task | Simultaneous Facial Information Matching Task; Facial Information Discrimination Task |
| Social Threat Task | Threat Reactivity Paradigm; Social Threat Task |
| Supraliminal Emotion(al) Face Matching Task | Supraliminal Emotional Face‐Matching Paradigm |
| Threat Face Matching Task | Threat Face Matching Task |
| Visual Matching Task | Visual Matching Task |

**Supplementary Tables 3a-3f: Synthesized Brain Region Labels**

Supplementary Table 3a: Subcortical Regions

| **Synthesized brain region labels** | **Original labels** |
| --- | --- |
| Amygdala | r amygdala |
|  | l amygdala |
|  | l centromedial amygdala |
|  | l superficial amygdala |
|  | r centromedial amygdala |
|  | r superficial amygdala |
|  | l basolateral amygdala |
|  | r basolateral amygdala |
|  | r amygdala (basolateral)  amygdalostriatal transition area |
| Basal Ganglia | l nucleus accumbens |
|  | r nucleus accumbens |
|  | l caudate |
|  | l pallidum |
|  | l putamen |
|  | r caudate |
|  | r pallidum |
|  | r putamen |
|  | l basal ganglia |
|  | l lateral global pallidus |
|  | l lentiform nucleus |
|  | r basal ganglia |
|  | r lentiform nucleus/putamen |
|  | r lentiform nucleus |
|  | l caudate nucleus |
|  | l globus pallidus |
|  | r caudate nucleus |
|  | r globus pallidus |
|  | r substantia nigra |
| Hippocampus | l hippocampus |
|  | r hippocampus |
|  | l hippocampal gyrus |
|  | r hippocampal gyrus |
| Hypothalamus | l hypothalamus |
|  | r hypothalamus |
| Parahippocampus | l parahippocampus |
|  | r parahippocampus |
|  | l parahippocampal gyrus |
|  | r parahippocampal gyrus |
| Thalamus | l thalamus |
|  | r thalamus |
|  | l lateral geniculate body |
|  | l dorsomedial thalamus |
|  | r dorsomedial thalamus |

Supplementary Table 3b: Frontal Regions

| **Synthesized brain region labels** | **Original labels** |
| --- | --- |
| Cingulate | l anterior cingulate cortex |
|  | l posterior cingulate cortex |
|  | r posterior cingulate cortex |
|  | l cingulate cortex subregions |
|  | r cingulate cortex subregions |
|  | r anterior cingulate cortex |
|  | l anterior cingulum |
|  | r anterior cingulum |
|  | r anterior cingulate |
| Cingulate | r cingulate gyrus |
|  | l cingulate gyrus |
|  | l posterior cingulate gyrus |
|  | r anterior cingulate sulcus |
|  | r middle cingulate gyrus |
|  | r middle cingulum |
|  | r cingulate cortex |
|  | l anterior cingulate gyrus |
|  | l cingulate cortex |
|  | l dorsal anterior cingulate cortex  r dorsal anterior cingulate cortex |
|  | l mid cingulate gyrus |
|  | l midcingulate cortex |
|  | r anterior cingulate gyrus |
|  | r midcingulate |
|  | l posterior cingulate |
|  | r posterior cingulate |
| Dorsolateral Prefrontal Cortex | l middle frontal gyrus |
|  | r superior frontal gyrus |
|  | r dorsolateral prefrontal cortex |
|  | l dorsolateral prefrontal cortex |
|  | l frontal middle gyrus |
|  | r frontal middle gyrus |
|  | r middle frontal gyrus |
|  | l superior frontal gyrus |
|  | r frontal eye fields |
|  | l middle frontal gyrus (dorsolateral prefrontal cortex) |
|  | r middle frontal gyrus (dorsolateral prefrontal cortex) |
|  | l frontal eye fields |
|  | l prefrontal cortex |
|  | r prefrontal cortex |
|  | l frontal cortex |
|  | r frontal cortex |
|  | l anterior prefrontal cortex |
|  | r anterior prefrontal cortex |
| Dorsomedial Prefrontal Cortex | l dorsal medial prefrontal cortex |
|  | r dorsal medial prefrontal cortex |
|  | l dorsomedial prefrontal cortex |
|  | r dorsomedial prefrontal cortex |
|  | l precentral gyrus |
|  | r precentral gyrus |
|  | l premotor cortex |
|  | r premotor cortex |
|  | l dorsal medial frontal gyrus |
|  | r frontal superior medial gyrus |
|  | r premotor |
|  | l precentral lobe |
|  | l presupplementary motor area |
|  | l superior medial prefrontal cortex |
|  | r precentral lobe |
|  | r presupplementary motor area |
|  | r superior medial prefrontal cortex |
|  | l prefrontal cortex |
|  | r prefrontal cortex |
| Dorsomedial Prefrontal Cortex | l medial frontal cortex |
|  | r medial frontal cortex |
|  | l medial prefrontal cortex |
|  | r medial prefrontal cortex |
|  | r frontal cortex |
|  | l frontal cortex |
|  | l anterior prefrontal cortex |
|  | r anterior prefrontal cortex |
| Orbitofrontal Cortex | l orbitofrontal cortex |
|  | l lateral orbitofrontal cortex |
|  | l medial orbitofrontal cortex |
|  | r lateral orbitofrontal cortex |
|  | r medial orbitofrontal cortex |
|  | r orbitofrontal cortex |
| Ventrolateral Prefrontal Cortex | l inferior frontal gyrus |
|  | l ventrolateral prefrontal cortex |
|  | r ventrolateral prefrontal cortex |
|  | l frontal inferior opercularis/triangularis/orbitalis gyrus |
|  | r frontal inferior opercularis/triangularis/orbitalis gyrus |
|  | r inferior frontal gyrus |
|  | l inferior frontal gyrus; l ventrolateral prefrontal cortex |
|  | l inferior frontal sulcus |
|  | r inferior frontal sulcus |
|  | r inferior frontal cortex |
|  | l lateral inferior frontal cortex |
|  | r frontal inferior triangularis |
|  | r inferior frontal gyrus opercularis |
|  | r inferior frontal gyrus triangularis |
|  | r lateral inferior frontal cortex |
|  | l inferior frontal gyrus (ventrolateral prefrontal cortex) |
|  | r inferior frontal gyrus (posterior triangularis) |
|  | r inferior frontal gyrus (ventrolateral prefrontal cortex) |
|  | l prefrontal cortex |
|  | r prefrontal cortex |
|  | r frontal cortex |
|  | l frontal cortex |
|  | l anterior prefrontal cortex |
|  | r anterior prefrontal cortex |
| Ventromedial Prefrontal Cortex | l ventromedial prefrontal cortex |
|  | r ventromedial prefrontal cortex |
|  | l subgenual cingulate cortex |
|  | r subgenual cingulate cortex |
|  | l pregenual cingulate cortex |
|  | r pregenual cingulate cortex |
|  | l subgenual anterior cingulate cortex |
|  | l supragenual anterior cingulate cortex |
|  | r subgenual anterior cingulate cortex |
|  | r supragenual anterior cingulate cortex |
| Ventromedial Prefrontal Cortex | l frontal pole |
|  | r frontal pole |
|  | l rectal gyrus |
|  | l subcallosal gyrus |
|  | l prefrontal cortex |
|  | r prefrontal cortex |
|  | l medial frontal cortex |
|  | r medial frontal cortex |
|  | l frontal cortex |
|  | l anterior prefrontal cortex |
|  | r anterior prefrontal cortex |

Supplementary Table 3c: Parietal Regions

| **Synthesized brain region labels** | **Original labels** |
| --- | --- |
| Inferior Parietal Lobe | l inferior parietal cortex |
|  | r inferior parietal cortex |
|  | r inferior parietal lobe |
|  | l inferior parietal lobe |
|  | l inferior parietal lobule |
|  | r angular gyrus |
|  | r inferior parietal gyrus |
|  | r inferior parietal lobule |
|  | l parietal inferior gyrus |
|  | l angular gyrus |
| Precuneus | l precuneus |
|  | r precuneus |
| Superior Parietal Lobe | l superior parietal lobe |
|  | r superior parietal lobe |
|  | l superior parietal cortex |
|  | r superior parietal cortex |

Supplementary Table 3d: Brainstem, Cerebellum

| **Synthesized brain region labels** | **Original labels** |
| --- | --- |
| Brainstem | l dorsal pons |
|  | r dorsal pons |
|  | r brainstem |
|  | l dorsal midbrain |
|  | l dorsomedial midbrain |
|  | r dorsal midbrain |
|  | r dorsomedial midbrain |
|  | l brain stem |
|  | r brain stem |
| Cerebellum | l cerebellum |
|  | r cerebellum |
|  | l cerebellar tonsil |
|  | l declive |
|  | r cerebellar tonsil |
|  | r declive |
|  | r vermis |
|  | l cerebellum (vi) |
|  | l cerebellum lobule ix |
|  | l cerebellum vi |
|  | r cerebellum lobule ix |
|  | l cerebellar declive |
|  | l cerebellar uvula |
|  | r cerebellar culmen |
|  | r cerebellar declive |

Supplementary Table 3e: Occipital Regions

| **Synthesized brain region labels** | **Original labels** |
| --- | --- |
| Cuneus | l cuneus |
|  | r cuneus |
|  | r cuneal cortex |
| Lingual Gyrus | l lingual gyrus |
|  | r lingual gyrus |
|  | r lingual |
| Primary Visual Cortex | l primary visual cortex |
|  | r primary visual cortex |
|  | r calcarine |
|  | l calcarine |
|  | l visual cortex |
|  | r visual cortex |
|  | l calcarine cortex |
|  | l calcarine gyrus |
|  | l occipital pole |
|  | r occipital pole |
|  | r calcarine gyrus |
|  | r inferior calcarine cortex |
|  | r superior calcarine cortex |
|  | l striate cortex |
|  | r striate cortex |
|  | l occipital lobe |
|  | r occipital lobe |
|  | r occipital gyrus |
|  | l occipital cortex |
|  | r occipital cortex |
|  | l occipital gyrus |
|  | r occipital |
|  | l occipital |
| Secondary Visual Cortex | r middle occipital gyrus |
|  | l superior occipital gyrus |
|  | r inferior occipital gyrus |
|  | l inferior occipital gyrus |
|  | l middle occipital gyrus |
|  | r superior occipital gyrus |
|  | r inferior occipital lobe |
|  | l lateral occipital cortex |
|  | l occipital face area |
|  | l occipital middle |
|  | r lateral occipital cortex |
|  | r occipital face area |
|  | l extrastriate visual cortex |
|  | l occipital middle gyrus |
|  | r lateral occipital area |
|  | l extra striate cortex |
|  | l extrastriate cortex |
|  | r extra striate cortex |
|  | r extrastriate cortex |
|  | r extrastriate visual cortex |
|  | l occipital lobe |
|  | r occipital lobe |
|  | l occipital cortex |
|  | r occipital cortex |
|  | r occipital |
|  | l occipital |

Supplementary Table 3f: Temporal Regions

| **Synthesized brain region labels** | **Original labels** |
| --- | --- |
| Fusiform Gyrus | r fusiform gyrus |
|  | l fusiform gyrus |
|  | r fusiform |
|  | l fusiform |
|  | l fusiform face area |
|  | r fusiform face area |
|  | l posterior fusiform gyrus |
|  | r posterior fusiform gyrus |
|  | l lateral fusiform gyrus |
|  | r lateral fusiform gyrus |
|  | l posterior fusiform gyrus |
|  | l posterior fusiform gyrus-put |
|  | r posterior fusiform gyrus-put |
|  | l fusiform gyri |
|  | r fusiform gyri |
| Insula | r insula |
|  | l insula |
|  | l anterior insula |
|  | l posterior insula |
|  | r anterior insula |
|  | r posterior insula |
|  | l mid insula |
|  | r mid insula |
| Inferior Temporal Gyrus | r inferior temporal gyrus |
|  | l inferior temporal gyrus |
|  | r temporal gyrus |
|  | l temporal lobe |
|  | r temporal lobe |
| Middle Temporal Gyrus | l middle temporal cortex |
|  | l middle temporal gyrus |
|  | r middle temporal gyrus |
|  | l entorhinal cortex |
|  | l medial temporal gyrus |
|  | l middle temporal regions |
|  | r medial temporal lobe |
|  | r middle temporal regions |
|  | r middle temporal cortex |
|  | r temporal gyrus |
|  | l temporal lobe |
|  | r temporal lobe |
| Superior Temporal Gyrus | l superior temporal gyrus |
|  | r superior temporal gyrus |
|  | l superior temporal sulcus |
|  | r superior temporal sulcus |
|  | r temporal gyrus |
| Temporal Pole | l temporal lobe |
|  | l temporal pole superior |
| Claustrum | l claustrum |
|  | r claustrum |

**Supplementary Table 4: Frequency of Task Names**

| **Standardised Name used** | **# Articles** | **% of Articles (total = 184)** |
| --- | --- | --- |
| Emotion(al) Face Matching Task | 37 | 20.10869565 |
| Face Match(ing) Task | 34 | 18.47826087 |
| Emotion(al) Face Processing Task | 8 | 4.347826087 |
| Robust paradigm for eliciting [amygdala/subcortical] [responsiveness/activation] used previously | 8 | 4.347826087 |
| Emotional Face(s) Task | 7 | 3.804347826 |
| Emotion(al) Matching Task | 6 | 3.260869565 |
| Emotion(al) Task | 6 | 3.260869565 |
| Hariri and colleagues (2002) | 5 | 2.717391304 |
| Hariri and colleagues (2002) - Modified | 5 | 2.717391304 |
| Amygdala Reactivity Paradigm | 4 | 2.173913043 |
| Emotional Faces Shifting Attention Task | 4 | 2.173913043 |
| Facial Emotion Processing Task | 4 | 2.173913043 |
| NA | 4 | 2.173913043 |
| Emotion Discrimination Task | 3 | 1.630434783 |
| Emotion Face Assessment Task | 3 | 1.630434783 |
| Emotion Processing Task | 3 | 1.630434783 |
| Emotion(al) Face Matching Task - Modified | 3 | 1.630434783 |
| Face Match(ing) Task - Modified | 3 | 1.630434783 |
| Face Processing Task | 3 | 1.630434783 |
| Faces Task | 3 | 1.630434783 |
| Facial Expression Discrimination Task | 2 | 1.086956522 |
| Implicit Emotion Processing Task | 2 | 1.086956522 |
| Implicit Emotion(al) Face Matching Task | 2 | 1.086956522 |
| Matching Task | 2 | 1.086956522 |
| Negative Emotional Face Processing Task | 2 | 1.086956522 |
| fMRI Task | 2 | 1.086956522 |
| Affect Labeling Face MatchingTask | 1 | 0.543478261 |
| Affective Face Matching Task | 1 | 0.543478261 |
| Aversive Face-Viewing Task | 1 | 0.543478261 |
| Behavioral fMRI Imaging Paradigm | 1 | 0.543478261 |
| Cortico-limbic Reactivity Task | 1 | 0.543478261 |
| Emotion Emotion Processing Task | 1 | 0.543478261 |
| Emotion Face Assessment Task - Modified | 1 | 0.543478261 |
| Emotion Recognition Task | 1 | 0.543478261 |
| Emotion(al) Face Assessment Task | 1 | 0.543478261 |
| Emotion(al) Face Face Matching Task | 1 | 0.543478261 |
| Emotion(al) Face Match(ing)/Label(ling) Task | 1 | 0.543478261 |
| Emotion(al) Match(ing)/Label(ling) Task | 1 | 0.543478261 |
| Emotion(al) Matching Task - Modified | 1 | 0.543478261 |
| Emotional Reactivity Task | 1 | 0.543478261 |
| Emotive Face Identification Task | 1 | 0.543478261 |
| Explicit/Implicit Processing Task | 1 | 0.543478261 |
| Face Identification task | 1 | 0.543478261 |
| Faces task | 1 | 0.543478261 |
| Facial Affect Matching Task | 1 | 0.543478261 |
| Facial Emotion Matching Task | 1 | 0.543478261 |
| Facial Emotion Recognition Paradigm | 1 | 0.543478261 |
| Facial Information Discrimination Task | 1 | 0.543478261 |
| Implicit Emotion Recognition Task | 1 | 0.543478261 |
| Implicit Facial Information Processing Task | 1 | 0.543478261 |
| Matching Faces Judgement Task | 1 | 0.543478261 |
| NimStim Emotional Faces Viewing Task | 1 | 0.543478261 |
| Perceptual Face Processing Task | 1 | 0.543478261 |
| Robust paradigm for eliciting [amygdala/subcortical] [responsiveness/activation] - Modified | 1 | 0.543478261 |
| Simultaenous Identity Matching Task | 1 | 0.543478261 |
| Simultaneous Affect Matching/Labeling Task | 1 | 0.543478261 |
| Simultaneous Facial Information Matching Task | 1 | 0.543478261 |
| Social Threat Task | 1 | 0.543478261 |
| Supraliminal Emotion(al) Face Matching Task | 1 | 0.543478261 |
| Threat Face Matching Task | 1 | 0.543478261 |
| Visual Matching Task | 1 | 0.543478261 |
| [Faces and Shapes] Affective Reactivity Task | 1 | 0.543478261 |

**Supplementary Table 5a: Frequency of Face Stimuli**

| **Stimuli Set/Sets** | **# Tasks** | **% of Number of tasks (total = 184)** |
| --- | --- | --- |
| Ekman Series | 63 | 34.23913043 |
| NA | 59 | 32.06521739 |
| NimStim-Set | 21 | 11.41304347 |
| Gur et. al., 2002 | 10 | 5.434782608 |
| Karolinska Directed Emotional Faces Set | 7 | 3.804347826 |
| Radboud Faces Database of Facial Affect | 6 | 3.260869565 |
| Chinese facial affective picture system (Gong et al., 2011) | 3 | 1.630434782 |
| NIMH Child Emotional Faces Picture Set | 2 | 1.086956522 |
| FACES database (Ebner et al., 2010) | 2 | 1.086956522 |
| Racially Diverse Affective Expression (RADIATE) facial stimulus set | 2 | 1.086956522 |
| NimStim-Set  The University of Pennsylvania Facial Emotional Stimuli  Psychological Image Collection at Stirling | 1 | 0.543478261 |
| Yan & Yuejia, 2005 | 1 | 0.543478261 |
| Ekman Series  NimStim-Set | 1 | 0.543478261 |
| IAPS | 1 | 0.543478261 |
| IAPS  Emotional Picture System (EmoPics)  Radboud Faces Database of Facial Affect | 1 | 0.543478261 |
| Ekman Series  Japanese and Caucasian facial expressions of emotion (JACFEE) | 1 | 0.543478261 |
| Fisher et al 2022 | 1 | 0.543478261 |
| Chinese facial affective picture system (CFAPS, Wang & Luo, 2005) | 1 | 0.543478261 |
| Chinese facial affective picture system (Yan and Yue-jia, 2005) | 1 | 0.543478261 |

**Supplementary Table 5b: Frequency of Facial Expressions**

| **Expressions used** | **# Tasks** | **% of Tasks (total = 184)** |
| --- | --- | --- |
| angry; fearful | 87 | 47.282609 |
| angry; fearful; happy | 29 | 15.760870 |
| angry; fearful; neutral; surprise | 11 | 5.978261 |
| angry; fearful; happy; neutral | 7 | 3.804348 |
| NA | 7 | 3.804348 |
| angry; fearful; neutral | 5 | 2.717391 |
| fearful; happy; neutral | 5 | 2.717391 |
| angry; fearful; happy; neutral; surprise; sad; disgust | 3 | 1.630435 |
| angry; fearful; sad | 3 | 1.630435 |
| angry; sad | 3 | 1.630435 |
| fearful | 3 | 1.630435 |
| fearful; happy; neutral; surprise | 3 | 1.630435 |
| neutral | 3 | 1.630435 |
| angry; fearful; happy; sad | 2 | 1.086957 |
| fearful; happy | 2 | 1.086957 |
| fearful; happy; neutral; sad | 2 | 1.086957 |
| angry; fearful; disgust | 1 | 0.543478 |
| angry; fearful; happy; neutral; sad | 1 | 0.543478 |
| angry; fearful; happy; neutral; surprise | 1 | 0.543478 |
| angry; fearful; happy; neutral; surprise; sad; disgust; contempt | 1 | 0.543478 |
| angry; fearful; happy; surprise; sad; disgust; contempt | 1 | 0.543478 |
| angry; fearful; positive | 1 | 0.543478 |
| angry; fearful; surprise | 1 | 0.543478 |
| angry; happy; neutral | 1 | 0.543478 |
| fearful; sad | 1 | 0.543478 |

**Supplementary Table 6a: Frequency of Primary Task Instructions**

| **Instructions** | **# Tasks** | **% of Tasks**  **(total = 184)** |
| --- | --- | --- |
| Match faces | 90 | 48.9130435 |
| Match emotion | 65 | 35.3260870 |
| Match expression | 8 | 4.3478261 |
| NA | 6 | 3.2608696 |
| Match identity | 5 | 2.7173913 |
| Match image | 4 | 2.1739130 |
| Match gender | 3 | 1.6304348 |
| Match affect | 2 | 1.0869565 |
| Match emotional valence | 1 | 0.5434783 |

**Supplementary Table 6b: Frequency of Secondary Task Instructions**

| **Instructions** | **# Tasks** | **% of Tasks**  **(total = 184)** |
| --- | --- | --- |
| NA | 167 | 90.7608696 |
| Label emotion | 7 | 3.8043478 |
| Match identity | 3 | 1.6304348 |
| Match sex | 2 | 1.0869565 |
| Match pictures | 1 | 0.5434783 |
| Label expression | 1 | 0.5434783 |
| Match gender | 1 | 0.5434783 |
| Identify emotions | 1 | 0.5434783 |
| Label emotion/Match gender | 1 | 0.5434783 |

**Supplementary Table 7: Frequency of Control Stimuli**

| **Stimuli Set/Sets** | **# Tasks** | **% of Tasks (total = 184)** |
| --- | --- | --- |
| Shapes | 176 | 95.6521739 |
| Non-social control stimuli | 4 | 2.5157233 |
| NA | 2 | 1.0869565 |
| Rectangles of pixelated patterns derived from neutral face picture | 1 | 0.5434783 |
| Rectangles of patterns of circles or squares | 1 | 0.5434783 |

**Supplementary Table 8: Frequency of Other Stimuli**

| **Stimuli Set/Sets** | **# Tasks** | **% of  Tasks (total = 184)** |
| --- | --- | --- |
| NA | 154 | 83.69565217 |
| Ekman series | 8 | 4.347826087 |
| NimStim-Set | 3 | 1.630434783 |
| Faces | 3 | 1.630434783 |
| IAPS | 2 | 1.086956522 |
| Gur et. al., 2002 | 2 | 1.086956522 |
| Threatening scenes | 2 | 1.086956522 |
| Ekman series  Affective labels: fearful/angry  Shapes and labels: circle/elipse | 1 | 0.543478261 |
| Macbrain database  The University of Pennsylvania Facial Emotional Stimuli  The Psychological Image Collection at Stirling | 1 | 0.543478261 |
| Facial Expression of Emotion: Stimuli and Tests | 1 | 0.543478261 |
| Ekman faces  Affective label | 1 | 0.543478261 |
| Karolinska Directed Emotional Faces Set | 1 | 0.543478261 |
| Doors** | 1 | 0.543478261 |
| National Institute of MentalHealth Child Emotional Faces Pictures Set (NIMH-ChEFS;Egger et al.,2011) | 1 | 0.543478261 |
| Fixation cross | 1 | 0.543478261 |
| Social Scences (IAPS and  Emotional Picture System (EmoPics) | 1 | 0.543478261 |
| Scenes | 1 | 0.543478261 |

**Supplementary Table 9a: Number of Face Blocks**

| **Number of Blocks** | **#  Tasks** | **% of  Tasks (total = 184)** |
| --- | --- | --- |
| 4 | 67 | 36.41304348 |
| 2 | 30 | 16.30434783 |
| 9 | 18 | 9.782608696 |
| 3 | 15 | 8.152173913 |
| 6 | 13 | 7.065217391 |
| NA | 10 | 5.434782609 |
| 8 | 9 | 4.891304348 |
| 5 | 7 | 3.804347826 |
| 12 | 6 | 3.260869565 |
| 10 | 3 | 1.630434783 |
| 7 | 2 | 1.086956522 |
| 18 | 2 | 1.086956522 |
| 16 | 1 | 0.543478261 |
| 1 | 1 | 0.543478261 |

**Supplementary Table 9b: Number of Control Blocks**

| **Number of Blocks** | **# Tasks** | **% of Tasks (total = 184)** |
| --- | --- | --- |
| 5 | 62 | 33.69565 |
| 3 | 36 | 19.56522 |
| 4 | 20 | 10.86957 |
| 6 | 15 | 8.152174 |
| NA | 11 | 5.978261 |
| 9 | 11 | 5.978261 |
| 2 | 8 | 4.347826 |
| 8 | 6 | 3.26087 |
| 7 | 3 | 1.630435 |
| 12 | 3 | 1.630435 |
| 11 | 2 | 1.086957 |
| Variable | 1 | 0.543478 |
| 16 | 1 | 0.543478 |
| 1 | 1 | 0.543478 |
| 10 | 1 | 0.543478 |
| 18 | 1 | 0.543478 |
| 14 | 1 | 0.543478 |
| 24 | 1 | 0.543478 |

**Supplementary Table 9c: Block Duration**

| **Duration (s = seconds)** | **# Tasks** | **% of Tasks (total = 184)** |
| --- | --- | --- |
| NA | 44 | 23.91304 |
| 30s | 43 | 23.36957 |
| 20s | 19 | 10.32609 |
| Faces: 48s; Shapes: 36s | 14 | 7.608696 |
| 32.5s | 9 | 4.891304 |
| 32s | 9 | 4.891304 |
| 24s | 7 | 3.804348 |
| 21s | 5 | 2.717391 |
| 31.8s | 3 | 1.630435 |
| Faces: 47s, Shapes: 35s | 3 | 1.630435 |
| Faces: 44.5s; Controls: 22s | 2 | 1.086957 |
| 42s | 2 | 1.086957 |
| 32.6s | 2 | 1.086957 |
| 36s | 2 | 1.086957 |
| 25s | 2 | 1.086957 |
| 22.5s | 2 | 1.086957 |
| 18s | 2 | 1.086957 |
| Faces: 82s; Controls: 42s | 2 | 1.086957 |
| Run 1: 16s; Run 2: 12s | 1 | 0.543478 |
| Faces/Scenes: 30s; Shapes: 15s | 1 | 0.543478 |
| Faces: 62s; Shapes: 38s | 1 | 0.543478 |
| faces: 48s; Controls: 36s | 1 | 0.543478 |
| faces: 47s; shapes: 35s | 1 | 0.543478 |
| Faces: 36-48s; Shapes: 36s | 1 | 0.543478 |
| Faces: 24s, Shapes: 20s | 1 | 0.543478 |
| 52s | 1 | 0.543478 |
| 44.5s | 1 | 0.543478 |
| 33s | 1 | 0.543478 |
| 26s | 1 | 0.543478 |
| 12s | 1 | 0.543478 |

**Supplementary Table 10a: Face Stimuli – Total Number of Trials**

| **Number of Trials** | **# Tasks** | **% of Tasks (total = 184)** |
| --- | --- | --- |
| 24 | 62 | 33.69565217 |
| 12 | 29 | 15.76086957 |
| NA | 18 | 9.782608696 |
| 36 | 16 | 8.695652174 |
| 48 | 10 | 5.434782609 |
| 18 | 9 | 4.891304348 |
| 54 | 7 | 3.804347826 |
| 30 | 6 | 3.260869565 |
| 72 | 6 | 3.260869565 |
| 60 | 3 | 1.630434783 |
| 16 | 2 | 1.086956522 |
| 56 | 2 | 1.086956522 |
| Variable | 2 | 1.086956522 |
| 32 | 2 | 1.086956522 |
| 80 | 2 | 1.086956522 |
| 45 | 2 | 1.086956522 |
| NA *self paced* | 2 | 1.086956522 |
| 52 | 1 | 0.543478261 |
| 96 | 1 | 0.543478261 |
| 15 | 1 | 0.543478261 |
| 6 | 1 | 0.543478261 |

**Supplementary Table 10b:Control Stimuli – Total Number of Trials**

| **Number of Trials** | **# Tasks** | **% of Tasks (total = 184)** |
| --- | --- | --- |
| 30 | 52 | 28.26087 |
| 18 | 31 | 16.84783 |
| NA | 24 | 13.04348 |
| 24 | 18 | 9.782609 |
| 36 | 15 | 8.152174 |
| 12 | 6 | 3.26087 |
| 54 | 5 | 2.717391 |
| 60 | 4 | 2.173913 |
| 48 | 4 | 2.173913 |
| 72 | 3 | 1.630435 |
| Variable | 3 | 1.630435 |
| 16 | 2 | 1.086957 |
| 80 | 2 | 1.086957 |
| 56 | 2 | 1.086957 |
| 15 | 2 | 1.086957 |
| NA *self paced* | 2 | 1.086957 |
| 66 | 2 | 1.086957 |
| 70 | 1 | 0.543478 |
| 52 | 1 | 0.543478 |
| 20 | 1 | 0.543478 |
| 64 | 1 | 0.543478 |
| 6 | 1 | 0.543478 |
| 42 | 1 | 0.543478 |
| 8 | 1 | 0.543478 |

**Supplementary Table 10c: Trial Duration**

| **Duration (s = seconds)** | **#  Tasks** | **% of  Tasks (total = 184)** |
| --- | --- | --- |
| 5s | 65 | 35.32608696 |
| 4s | 52 | 28.26086957 |
| NA | 20 | 10.86956522 |
| 3s | 9 | 4.891304348 |
| 2s | 9 | 4.891304348 |
| 4.5s | 5 | 2.717391304 |
| Variable | 5 | 2.717391304 |
| 5.3s | 3 | 1.630434783 |
| 5.4s | 3 | 1.630434783 |
| 7s | 2 | 1.086956522 |
| 2.5s (slightly jittered in 12 different steps to vary by 0–50 ms around the mean) | 2 | 1.086956522 |
| 3.75s | 2 | 1.086956522 |
| Faces: 5s  Control: 4s | 1 | 0.543478261 |
| 3.3s | 1 | 0.543478261 |
| Faces/Scenes: 5s  Shapes: 2.5s | 1 | 0.543478261 |
| 8s | 1 | 0.543478261 |
| NA *self paced* | 1 | 0.543478261 |
| 2.5s | 1 | 0.543478261 |
| Faces: 6s  Shapes: 4s | 1 | 0.543478261 |

**Supplementary Table 11: Statistical Thresholds Applied for the Faces > Shapes Contrast in Healthy Controls**

Numbers indicate how often each threshold was applied and how often each brain region was reportedly activated. **ROI/SVC contrasts:** R1: uncorrected; R2: FDR corrected; R3: FWE corrected; R4: other unspecified multiple corrections. **Whole brain contrasts:** W1: uncorrected; W2: FDR corrected; W3: FWE corrected; W4: other unspecified multiple corrections; W5: Other.

| **Region** | **R1**  **Searched** | **R1**  **Activated** | **R2**  **Searched** | **R2**  **Activated** | **R3**  **Searched** | **R3**  **Activated** | **R4**  **Searched** | **R4**  **Activated** | **W1**  **Applied** | **W1**  **Activated** | **W2**  **Applied** | **W2**  **Activated** | **W3**  **Applied** | **W3**  **Activated** | **W4**  **Applied** | **W4**  **Activated** | **W5**  **Applied** | **W5**  **Activated** |
| --- | --- | --- | --- | --- | --- | --- | --- | --- | --- | --- | --- | --- | --- | --- | --- | --- | --- | --- |
| **Amygdala** | 31 | 28 | 30 | 26 | 63 | 51 | 38 | 22 | 19 | 14 | 8 | 7 | 39 | 39 | 17 | 17 | 8 | 13 |
| **Basal Ganglia** | 0 | 0 | 5 | 2 | 0 | 0 | 0 | 0 | 19 | 8 | 8 | 4 | 39 | 6 | 17 | 3 | 8 | 0 |
| **Brainstem** | 0 | 0 | 2 | 0 | 0 | 0 | 0 | 0 | 19 | 1 | 8 | 0 | 39 | 4 | 17 | 0 | 8 | 4 |
| **Cerebellum** | 1 | 1 | 0 | 0 | 0 | 0 | 0 | 0 | 19 | 7 | 8 | 5 | 39 | 25 | 17 | 14 | 8 | 1 |
| **Cingulate** | 0 | 0 | 6 | 2 | 2 | 0 | 0 | 0 | 19 | 3 | 8 | 4 | 39 | 31 | 17 | 13 | 8 | 0 |
| **Claustrum** | 0 | 0 | 0 | 0 | 0 | 0 | 0 | 0 | 19 | 4 | 8 | 0 | 39 | 1 | 17 | 0 | 8 | 0 |
| **Frontal dlPFC** | 5 | 1 | 2 | 1 | 2 | 0 | 0 | 0 | 19 | 21 | 8 | 17 | 39 | 47 | 17 | 16 | 8 | 8 |
| **Frontal dmPFC** | 5 | 0 | 6 | 0 | 3 | 0 | 2 | 0 | 19 | 5 | 8 | 13 | 39 | 30 | 17 | 6 | 8 | 2 |
| **Frontal vlPFC** | 4 | 0 | 4 | 0 | 2 | 0 | 4 | 0 | 19 | 14 | 8 | 12 | 39 | 47 | 17 | 8 | 8 | 0 |
| **Frontal vmPFC** | 5 | 0 | 12 | 1 | 0 | 0 | 0 | 0 | 19 | 0 | 8 | 8 | 39 | 27 | 17 | 6 | 8 | 0 |
| **Fusiform** | 5 | 4 | 2 | 0 | 2 | 2 | 7 | 5 | 19 | 11 | 8 | 10 | 39 | 78 | 17 | 44 | 8 | 8 |
| **Hippocampus** | 2 | 2 | 4 | 2 | 2 | 0 | 0 | 0 | 19 | 8 | 8 | 3 | 39 | 24 | 17 | 8 | 8 | 0 |
| **Hypothalamus** | 0 | 0 | 2 | 0 | 0 | 0 | 0 | 0 | 19 | 0 | 8 | 0 | 39 | 0 | 17 | 0 | 8 | 0 |
| **Insula** | 7 | 3 | 6 | 4 | 5 | 5 | 4 | 2 | 19 | 6 | 8 | 0 | 39 | 20 | 17 | 0 | 8 | 0 |
| **Lingual Gyrus** | 0 | 0 | 0 | 0 | 0 | 0 | 0 | 0 | 19 | 5 | 8 | 2 | 39 | 16 | 17 | 12 | 8 | 1 |
| **Occipital Cuneus** | 0 | 0 | 0 | 0 | 0 | 0 | 0 | 0 | 19 | 2 | 8 | 0 | 39 | 6 | 17 | 2 | 8 | 0 |
| **Occipital V2** | 0 | 0 | 0 | 0 | 0 | 0 | 1 | 0 | 19 | 11 | 8 | 3 | 39 | 58 | 17 | 39 | 8 | 4 |
| **Occipital V1** | 0 | 0 | 0 | 0 | 0 | 0 | 3 | 0 | 19 | 13 | 8 | 7 | 39 | 36 | 17 | 18 | 8 | 5 |
| **OFC** | 0 | 0 | 0 | 0 | 2 | 0 | 0 | 0 | 19 | 6 | 8 | 2 | 39 | 3 | 17 | 0 | 8 | 0 |
| **Parahippocampus** | 0 | 0 | 1 | 0 | 0 | 0 | 0 | 0 | 19 | 5 | 8 | 1 | 39 | 4 | 17 | 9 | 8 | 0 |
| **Parietal Inferior** | 0 | 0 | 2 | 0 | 0 | 0 | 0 | 0 | 19 | 5 | 8 | 2 | 39 | 8 | 17 | 4 | 8 | 1 |
| **Parietal Superior** | 0 | 0 | 0 | 0 | 0 | 0 | 0 | 0 | 19 | 3 | 8 | 1 | 39 | 8 | 17 | 3 | 8 | 0 |
| **Precuneus** | 2 | 0 | 0 | 0 | 0 | 0 | 0 | 0 | 19 | 7 | 8 | 2 | 39 | 13 | 17 | 2 | 8 | 1 |
| **Temporal Inferior** | 0 | 0 | 0 | 0 | 0 | 0 | 1 | 0 | 19 | 6 | 8 | 2 | 39 | 4 | 17 | 0 | 8 | 0 |
| **Temporal Middle** | 0 | 0 | 1 | 0 | 0 | 0 | 2 | 0 | 19 | 12 | 8 | 4 | 39 | 11 | 17 | 1 | 8 | 1 |
| **Temporal Pole** | 0 | 0 | 0 | 0 | 0 | 0 | 0 | 0 | 19 | 0 | 8 | 0 | 39 | 3 | 17 | 0 | 8 | 0 |
| **TemporalSuperior** | 0 | 0 | 0 | 0 | 0 | 0 | 2 | 0 | 19 | 12 | 8 | 3 | 39 | 10 | 17 | 0 | 8 | 0 |
| **Thalamus** | 0 | 0 | 2 | 1 | 0 | 0 | 0 | 0 | 19 | 6 | 8 | 3 | 39 | 22 | 17 | 7 | 8 | 3 |
| **NOSIGDIF** | 0 | 0 | 0 | 0 | 0 | 0 | 0 | 0 | 19 | 0 | 8 | 0 | 39 | 0 | 17 | 0 | 8 | 0 |

**Supplementary Tables 12a – 12l: Statistical Thresholds Applied for the Patients > Controls: Faces > Shapes Contrast**

Supplementary Table 12a: Major Depressive Disorder

Numbers indicate how often each threshold was applied and how often each brain region was reportedly more activated in patients than controls. **ROI/SVC contrasts:** R1: uncorrected; R2: FDR corrected; R3: FWE corrected; R4: other unspecified multiple corrections. **Whole brain contrasts:** W1: uncorrected; W2: FDR corrected; W3: FWE corrected; W4: other unspecified multiple corrections; W5: Other.

| **Region** | **R1**  **Searched** | **R1**  **Activated** | **R2**  **Searched** | **R2**  **Activated** | **R3**  **Searched** | **R3**  **Activated** | **R4**  **Searched** | **R4**  **Activated** | **W1**  **Applied** | **W1**  **Activated** | **W2**  **Applied** | **W2**  **Activated** | **W3**  **Applied** | **W3**  **Activated** | **W4**  **Applied** | **W4**  **Activated** | **W5**  **Applied** | **W5**  **Activated** |
| --- | --- | --- | --- | --- | --- | --- | --- | --- | --- | --- | --- | --- | --- | --- | --- | --- | --- | --- |
| **Amygdala** | 6 | 2 | 2 | 0 | 25 | 4 | 6 | 4 | 5 | 0 | 0 | 0 | 17 | 0 | 4 | 0 | 2 | 0 |
| **Basal Ganglia** | 0 | 0 | 0 | 0 | 0 | 0 | 0 | 0 | 5 | 1 | 0 | 0 | 17 | 0 | 4 | 0 | 2 | 0 |
| **Brainstem** | 0 | 0 | 0 | 0 | 0 | 0 | 0 | 0 | 5 | 0 | 0 | 0 | 17 | 0 | 4 | 0 | 2 | 0 |
| **Cerebellum** | 0 | 0 | 0 | 0 | 0 | 0 | 0 | 0 | 5 | 1 | 0 | 0 | 17 | 0 | 4 | 0 | 2 | 1 |
| **Cingulate** | 1 | 1 | 0 | 0 | 3 | 1 | 0 | 0 | 5 | 8 | 0 | 0 | 17 | 14 | 4 | 0 | 2 | 4 |
| **Claustrum** | 0 | 0 | 0 | 0 | 0 | 0 | 0 | 0 | 5 | 0 | 0 | 0 | 17 | 0 | 4 | 0 | 2 | 0 |
| **Frontal dlPFC** | 2 | 0 | 0 | 0 | 3 | 0 | 0 | 0 | 5 | 8 | 0 | 0 | 17 | 2 | 4 | 0 | 2 | 1 |
| **Frontal dmPFC** | 2 | 0 | 0 | 0 | 4 | 0 | 0 | 0 | 5 | 2 | 0 | 0 | 17 | 4 | 4 | 0 | 2 | 0 |
| **Frontal vlPFC** | 2 | 0 | 0 | 0 | 3 | 0 | 0 | 0 | 5 | 2 | 0 | 0 | 17 | 0 | 4 | 0 | 2 | 0 |
| **Frontal vmPFC** | 2 | 0 | 0 | 0 | 3 | 0 | 0 | 0 | 5 | 0 | 0 | 0 | 17 | 0 | 4 | 0 | 2 | 2 |
| **Fusiform** | 0 | 0 | 0 | 0 | 0 | 0 | 0 | 0 | 5 | 1 | 0 | 0 | 17 | 0 | 4 | 0 | 2 | 0 |
| **Hippocampus** | 2 | 0 | 0 | 0 | 4 | 2 | 0 | 0 | 5 | 1 | 0 | 0 | 17 | 0 | 4 | 0 | 2 | 0 |
| **Hypothalamus** | 0 | 0 | 0 | 0 | 0 | 0 | 0 | 0 | 5 | 0 | 0 | 0 | 17 | 0 | 4 | 0 | 2 | 0 |
| **Insula** | 2 | 2 | 0 | 0 | 2 | 1 | 0 | 0 | 5 | 2 | 0 | 0 | 17 | 1 | 4 | 0 | 2 | 0 |
| **Lingual Gyrus** | 0 | 0 | 0 | 0 | 0 | 0 | 0 | 0 | 5 | 3 | 0 | 0 | 17 | 2 | 4 | 0 | 2 | 0 |
| **Occipital Cuneus** | 0 | 0 | 0 | 0 | 0 | 0 | 0 | 0 | 5 | 0 | 0 | 0 | 17 | 1 | 4 | 0 | 2 | 0 |
| **Occipital V2** | 0 | 0 | 0 | 0 | 0 | 0 | 0 | 0 | 5 | 1 | 0 | 0 | 17 | 1 | 4 | 0 | 2 | 0 |
| **Occipital V1** | 0 | 0 | 0 | 0 | 0 | 0 | 0 | 0 | 5 | 0 | 0 | 0 | 17 | 1 | 4 | 0 | 2 | 0 |
| **OFC** | 0 | 0 | 0 | 0 | 0 | 0 | 0 | 0 | 5 | 0 | 0 | 0 | 17 | 1 | 4 | 0 | 2 | 0 |
| **Parahippocampus** | 0 | 0 | 0 | 0 | 1 | 1 | 0 | 0 | 5 | 3 | 0 | 0 | 17 | 1 | 4 | 0 | 2 | 0 |
| **Parietal Inferior** | 0 | 0 | 0 | 0 | 0 | 0 | 0 | 0 | 5 | 0 | 0 | 0 | 17 | 1 | 4 | 0 | 2 | 1 |
| **Parietal Superior** | 0 | 0 | 0 | 0 | 0 | 0 | 0 | 0 | 5 | 0 | 0 | 0 | 17 | 3 | 4 | 0 | 2 | 0 |
| **Precuneus** | 0 | 0 | 0 | 0 | 0 | 0 | 0 | 0 | 5 | 2 | 0 | 0 | 17 | 2 | 4 | 0 | 2 | 0 |
| **Temporal Inferior** | 0 | 0 | 0 | 0 | 0 | 0 | 0 | 0 | 5 | 1 | 0 | 0 | 17 | 1 | 4 | 0 | 2 | 0 |
| **Temporal Middle** | 0 | 0 | 0 | 0 | 0 | 0 | 0 | 0 | 5 | 7 | 0 | 0 | 17 | 0 | 4 | 0 | 2 | 1 |
| **Temporal Pole** | 0 | 0 | 0 | 0 | 0 | 0 | 0 | 0 | 5 | 3 | 0 | 0 | 17 | 0 | 4 | 0 | 2 | 0 |
| **TemporalSuperior** | 0 | 0 | 0 | 0 | 0 | 0 | 0 | 0 | 5 | 3 | 0 | 0 | 17 | 1 | 4 | 0 | 2 | 0 |
| **Thalamus** | 0 | 0 | 0 | 0 | 0 | 0 | 0 | 0 | 5 | 1 | 0 | 0 | 17 | 0 | 4 | 0 | 2 | 0 |
| **NOSIGDIF** | 0 | 0 | 0 | 0 | 0 | 0 | 0 | 0 | 5 | 0 | 0 | 0 | 17 | 11 | 4 | 2 | 2 | 1 |

Supplementary Table 12b: Anxiety(-Related) Disorders

Numbers indicate how often each threshold was applied and how often each brain region was reportedly more activated in patients than controls. **ROI/SVC contrasts:** R1: uncorrected; R2: FDR corrected; R3: FWE corrected; R4: other unspecified multiple corrections. **Whole brain contrasts:** W1: uncorrected; W2: FDR corrected; W3: FWE corrected; W4: other unspecified multiple corrections; W5: Other.

| **Region** | **R1**  **Searched** | **R1**  **Activated** | **R2**  **Searched** | **R2**  **Activated** | **R3**  **Searched** | **R3**  **Activated** | **R4**  **Searched** | **R4**  **Activated** | **W1**  **Applied** | **W1**  **Activated** | **W2**  **Applied** | **W2**  **Activated** | **W3**  **Applied** | **W3**  **Activated** | **W4**  **Applied** | **W4**  **Activated** | **W5**  **Applied** | **W5**  **Activated** |
| --- | --- | --- | --- | --- | --- | --- | --- | --- | --- | --- | --- | --- | --- | --- | --- | --- | --- | --- |
| **Amygdala** | 4 | 2 | 2 | 0 | 16 | 3 | 4 | 2 | 6 | 1 | 0 | 0 | 10 | 0 | 0 | 0 | 2 | 0 |
| **Basal Ganglia** | 0 | 0 | 0 | 0 | 0 | 0 | 0 | 0 | 6 | 0 | 0 | 0 | 10 | 0 | 0 | 0 | 2 | 0 |
| **Brainstem** | 0 | 0 | 0 | 0 | 0 | 0 | 0 | 0 | 6 | 0 | 0 | 0 | 10 | 0 | 0 | 0 | 2 | 0 |
| **Cerebellum** | 0 | 0 | 0 | 0 | 0 | 0 | 0 | 0 | 6 | 1 | 0 | 0 | 10 | 1 | 0 | 0 | 2 | 1 |
| **Cingulate** | 0 | 0 | 0 | 0 | 10 | 0 | 0 | 0 | 6 | 8 | 0 | 0 | 10 | 3 | 0 | 0 | 2 | 4 |
| **Claustrum** | 0 | 0 | 0 | 0 | 0 | 0 | 0 | 0 | 6 | 0 | 0 | 0 | 10 | 0 | 0 | 0 | 2 | 0 |
| **Frontal dlPFC** | 0 | 0 | 0 | 0 | 0 | 0 | 0 | 0 | 6 | 7 | 0 | 0 | 10 | 2 | 0 | 0 | 2 | 1 |
| **Frontal dmPFC** | 0 | 0 | 0 | 0 | 0 | 0 | 0 | 0 | 6 | 2 | 0 | 0 | 10 | 4 | 0 | 0 | 2 | 0 |
| **Frontal vlPFC** | 0 | 0 | 0 | 0 | 0 | 0 | 0 | 0 | 6 | 2 | 0 | 0 | 10 | 0 | 0 | 0 | 2 | 0 |
| **Frontal vmPFC** | 0 | 0 | 0 | 0 | 0 | 0 | 0 | 0 | 6 | 0 | 0 | 0 | 10 | 0 | 0 | 0 | 2 | 2 |
| **Fusiform** | 0 | 0 | 0 | 0 | 0 | 0 | 0 | 0 | 6 | 1 | 0 | 0 | 10 | 0 | 0 | 0 | 2 | 0 |
| **Hippocampus** | 0 | 0 | 0 | 0 | 4 | 2 | 0 | 0 | 6 | 0 | 0 | 0 | 10 | 0 | 0 | 0 | 2 | 0 |
| **Hypothalamus** | 0 | 0 | 0 | 0 | 0 | 0 | 0 | 0 | 6 | 0 | 0 | 0 | 10 | 0 | 0 | 0 | 2 | 0 |
| **Insula** | 2 | 2 | 0 | 0 | 10 | 3 | 2 | 2 | 6 | 1 | 0 | 0 | 10 | 2 | 0 | 0 | 2 | 0 |
| **Lingual Gyrus** | 0 | 0 | 0 | 0 | 0 | 0 | 0 | 0 | 6 | 2 | 0 | 0 | 10 | 2 | 0 | 0 | 2 | 0 |
| **Occipital Cuneus** | 0 | 0 | 0 | 0 | 0 | 0 | 0 | 0 | 6 | 0 | 0 | 0 | 10 | 1 | 0 | 0 | 2 | 0 |
| **Occipital V2** | 0 | 0 | 0 | 0 | 0 | 0 | 0 | 0 | 6 | 1 | 0 | 0 | 10 | 3 | 0 | 0 | 2 | 0 |
| **Occipital V1** | 0 | 0 | 0 | 0 | 0 | 0 | 0 | 0 | 6 | 0 | 0 | 0 | 10 | 1 | 0 | 0 | 2 | 0 |
| **OFC** | 0 | 0 | 0 | 0 | 0 | 0 | 0 | 0 | 6 | 0 | 0 | 0 | 10 | 1 | 0 | 0 | 2 | 0 |
| **Parahippocampus** | 0 | 0 | 0 | 0 | 1 | 1 | 0 | 0 | 6 | 2 | 0 | 0 | 10 | 0 | 0 | 0 | 2 | 0 |
| **Parietal Inferior** | 0 | 0 | 0 | 0 | 0 | 0 | 0 | 0 | 6 | 0 | 0 | 0 | 10 | 2 | 0 | 0 | 2 | 1 |
| **Parietal Superior** | 0 | 0 | 0 | 0 | 0 | 0 | 0 | 0 | 6 | 0 | 0 | 0 | 10 | 3 | 0 | 0 | 2 | 0 |
| **Precuneus** | 0 | 0 | 0 | 0 | 0 | 0 | 0 | 0 | 6 | 1 | 0 | 0 | 10 | 4 | 0 | 0 | 2 | 0 |
| **Temporal Inferior** | 0 | 0 | 0 | 0 | 0 | 0 | 0 | 0 | 6 | 1 | 0 | 0 | 10 | 1 | 0 | 0 | 2 | 0 |
| **Temporal Middle** | 0 | 0 | 0 | 0 | 0 | 0 | 0 | 0 | 6 | 7 | 0 | 0 | 10 | 0 | 0 | 0 | 2 | 1 |
| **Temporal Pole** | 0 | 0 | 0 | 0 | 0 | 0 | 0 | 0 | 6 | 3 | 0 | 0 | 10 | 0 | 0 | 0 | 2 | 0 |
| **TemporalSuperior** | 0 | 0 | 0 | 0 | 2 | 0 | 0 | 0 | 6 | 3 | 0 | 0 | 10 | 3 | 0 | 0 | 2 | 0 |
| **Thalamus** | 0 | 0 | 0 | 0 | 0 | 0 | 0 | 0 | 6 | 1 | 0 | 0 | 10 | 0 | 0 | 0 | 2 | 0 |
| **NOSIGDIF** | 0 | 0 | 0 | 0 | 0 | 0 | 0 | 0 | 6 | 1 | 0 | 0 | 10 | 4 | 0 | 0 | 2 | 1 |

Supplementary Table 12c: Obsessive-Compulsive Disorder

Numbers indicate how often each threshold was applied and how often each brain region was reportedly more activated in patients than controls. **ROI/SVC contrasts:** R1: uncorrected; R2: FDR corrected; R3: FWE corrected; R4: other unspecified multiple corrections. **Whole brain contrasts:** W1: uncorrected; W2: FDR corrected; W3: FWE corrected; W4: other unspecified multiple corrections; W5: Other.

| **Region** | **R1**  **Searched** | **R1**  **Activated** | **R2**  **Searched** | **R2**  **Activated** | **R3**  **Searched** | **R3**  **Activated** | **R4**  **Searched** | **R4**  **Activated** | **W1**  **Applied** | **W1**  **Activated** | **W2**  **Applied** | **W2**  **Activated** | **W3**  **Applied** | **W3**  **Activated** | **W4**  **Applied** | **W4**  **Activated** | **W5**  **Applied** | **W5**  **Activated** |
| --- | --- | --- | --- | --- | --- | --- | --- | --- | --- | --- | --- | --- | --- | --- | --- | --- | --- | --- |
| **Amygdala** | 2 | 1 | 2 | 0 | 6 | 4 | 2 | 2 | 5 | 1 | 0 | 0 | 2 | 0 | 0 | 0 | 1 | 0 |
| **Basal Ganglia** | 0 | 0 | 0 | 0 | 0 | 0 | 0 | 0 | 5 | 0 | 0 | 0 | 2 | 0 | 0 | 0 | 1 | 0 |
| **Brainstem** | 0 | 0 | 0 | 0 | 0 | 0 | 0 | 0 | 5 | 0 | 0 | 0 | 2 | 0 | 0 | 0 | 1 | 0 |
| **Cerebellum** | 0 | 0 | 0 | 0 | 0 | 0 | 0 | 0 | 5 | 1 | 0 | 0 | 2 | 0 | 0 | 0 | 1 | 1 |
| **Cingulate** | 0 | 0 | 0 | 0 | 2 | 0 | 0 | 0 | 5 | 8 | 0 | 0 | 2 | 0 | 0 | 0 | 1 | 4 |
| **Claustrum** | 0 | 0 | 0 | 0 | 0 | 0 | 0 | 0 | 5 | 0 | 0 | 0 | 2 | 0 | 0 | 0 | 1 | 0 |
| **Frontal dlPFC** | 0 | 0 | 0 | 0 | 0 | 0 | 0 | 0 | 5 | 10 | 0 | 0 | 2 | 1 | 0 | 0 | 1 | 1 |
| **Frontal dmPFC** | 0 | 0 | 0 | 0 | 0 | 0 | 0 | 0 | 5 | 3 | 0 | 0 | 2 | 2 | 0 | 0 | 1 | 0 |
| **Frontal vlPFC** | 0 | 0 | 0 | 0 | 0 | 0 | 0 | 0 | 5 | 3 | 0 | 0 | 2 | 0 | 0 | 0 | 1 | 0 |
| **Frontal vmPFC** | 0 | 0 | 0 | 0 | 0 | 0 | 0 | 0 | 5 | 0 | 0 | 0 | 2 | 0 | 0 | 0 | 1 | 2 |
| **Fusiform** | 0 | 0 | 0 | 0 | 0 | 0 | 0 | 0 | 5 | 2 | 0 | 0 | 2 | 0 | 0 | 0 | 1 | 0 |
| **Hippocampus** | 0 | 0 | 0 | 0 | 2 | 2 | 0 | 0 | 5 | 1 | 0 | 0 | 2 | 0 | 0 | 0 | 1 | 0 |
| **Hypothalamus** | 0 | 0 | 0 | 0 | 0 | 0 | 0 | 0 | 5 | 0 | 0 | 0 | 2 | 0 | 0 | 0 | 1 | 0 |
| **Insula** | 2 | 2 | 0 | 0 | 4 | 1 | 0 | 0 | 5 | 0 | 0 | 0 | 2 | 1 | 0 | 0 | 1 | 0 |
| **Lingual Gyrus** | 0 | 0 | 0 | 0 | 0 | 0 | 0 | 0 | 5 | 2 | 0 | 0 | 2 | 1 | 0 | 0 | 1 | 0 |
| **Occipital Cuneus** | 0 | 0 | 0 | 0 | 0 | 0 | 0 | 0 | 5 | 0 | 0 | 0 | 2 | 0 | 0 | 0 | 1 | 0 |
| **Occipital V2** | 0 | 0 | 0 | 0 | 0 | 0 | 0 | 0 | 5 | 2 | 0 | 0 | 2 | 0 | 0 | 0 | 1 | 0 |
| **Occipital V1** | 0 | 0 | 0 | 0 | 0 | 0 | 0 | 0 | 5 | 1 | 0 | 0 | 2 | 1 | 0 | 0 | 1 | 0 |
| **OFC** | 0 | 0 | 0 | 0 | 2 | 0 | 0 | 0 | 5 | 0 | 0 | 0 | 2 | 1 | 0 | 0 | 1 | 0 |
| **Parahippocampus** | 0 | 0 | 0 | 0 | 1 | 1 | 0 | 0 | 5 | 3 | 0 | 0 | 2 | 0 | 0 | 0 | 1 | 0 |
| **Parietal Inferior** | 0 | 0 | 0 | 0 | 0 | 0 | 0 | 0 | 5 | 1 | 0 | 0 | 2 | 0 | 0 | 0 | 1 | 1 |
| **Parietal Superior** | 0 | 0 | 0 | 0 | 0 | 0 | 0 | 0 | 5 | 0 | 0 | 0 | 2 | 1 | 0 | 0 | 1 | 0 |
| **Precuneus** | 0 | 0 | 0 | 0 | 0 | 0 | 0 | 0 | 5 | 2 | 0 | 0 | 2 | 0 | 0 | 0 | 1 | 0 |
| **Temporal Inferior** | 0 | 0 | 0 | 0 | 0 | 0 | 0 | 0 | 5 | 1 | 0 | 0 | 2 | 0 | 0 | 0 | 1 | 0 |
| **Temporal Middle** | 0 | 0 | 0 | 0 | 0 | 0 | 0 | 0 | 5 | 7 | 0 | 0 | 2 | 0 | 0 | 0 | 1 | 1 |
| **Temporal Pole** | 0 | 0 | 0 | 0 | 0 | 0 | 0 | 0 | 5 | 3 | 0 | 0 | 2 | 0 | 0 | 0 | 1 | 0 |
| **TemporalSuperior** | 0 | 0 | 0 | 0 | 0 | 0 | 0 | 0 | 5 | 3 | 0 | 0 | 2 | 1 | 0 | 0 | 1 | 0 |
| **Thalamus** | 0 | 0 | 0 | 0 | 0 | 0 | 0 | 0 | 5 | 2 | 0 | 0 | 2 | 0 | 0 | 0 | 1 | 0 |
| **NOSIGDIF** | 0 | 0 | 0 | 0 | 0 | 0 | 0 | 0 | 5 | 0 | 0 | 0 | 2 | 1 | 0 | 0 | 1 | 0 |

Supplementary Table 12d: Bipolar Disorder

Numbers indicate how often each threshold was applied and how often each brain region was reportedly more activated in patients than controls. **ROI/SVC contrasts:** R1: uncorrected; R2: FDR corrected; R3: FWE corrected; R4: other unspecified multiple corrections. **Whole brain contrasts:** W1: uncorrected; W2: FDR corrected; W3: FWE corrected; W4: other unspecified multiple corrections; W5: Other.

| **Region** | **R1**  **Searched** | **R1**  **Activated** | **R2**  **Searched** | **R2**  **Activated** | **R3**  **Searched** | **R3**  **Activated** | **R4**  **Searched** | **R4**  **Activated** | **W1**  **Applied** | **W1**  **Activated** | **W2**  **Applied** | **W2**  **Activated** | **W3**  **Applied** | **W3**  **Activated** | **W4**  **Applied** | **W4**  **Activated** | **W5**  **Applied** | **W5**  **Activated** |
| --- | --- | --- | --- | --- | --- | --- | --- | --- | --- | --- | --- | --- | --- | --- | --- | --- | --- | --- |
| **Amygdala** | 1 | 0 | 0 | 0 | 7 | 0 | 2 | 1 | 0 | 0 | 0 | 0 | 5 | 1 | 0 | 0 | 0 | 0 |
| **Basal Ganglia** | 2 | 0 | 0 | 0 | 0 | 0 | 0 | 0 | 0 | 0 | 0 | 0 | 5 | 1 | 0 | 0 | 0 | 0 |
| **Brainstem** | 0 | 0 | 0 | 0 | 0 | 0 | 0 | 0 | 0 | 0 | 0 | 0 | 5 | 0 | 0 | 0 | 0 | 0 |
| **Cerebellum** | 0 | 0 | 0 | 0 | 0 | 0 | 0 | 0 | 0 | 0 | 0 | 0 | 5 | 0 | 0 | 0 | 0 | 0 |
| **Cingulate** | 1 | 0 | 0 | 0 | 0 | 0 | 0 | 0 | 0 | 0 | 0 | 0 | 5 | 2 | 0 | 0 | 0 | 0 |
| **Claustrum** | 0 | 0 | 0 | 0 | 0 | 0 | 0 | 0 | 0 | 0 | 0 | 0 | 5 | 0 | 0 | 0 | 0 | 0 |
| **Frontal dlPFC** | 1 | 0 | 0 | 0 | 0 | 0 | 0 | 0 | 0 | 0 | 0 | 0 | 5 | 3 | 0 | 0 | 0 | 0 |
| **Frontal dmPFC** | 0 | 0 | 0 | 0 | 0 | 0 | 0 | 0 | 0 | 0 | 0 | 0 | 5 | 0 | 0 | 0 | 0 | 0 |
| **Frontal vlPFC** | 0 | 0 | 0 | 0 | 0 | 0 | 0 | 0 | 0 | 0 | 0 | 0 | 5 | 0 | 0 | 0 | 0 | 0 |
| **Frontal vmPFC** | 0 | 0 | 0 | 0 | 0 | 0 | 0 | 0 | 0 | 0 | 0 | 0 | 5 | 0 | 0 | 0 | 0 | 0 |
| **Fusiform** | 0 | 0 | 0 | 0 | 0 | 0 | 0 | 0 | 0 | 0 | 0 | 0 | 5 | 0 | 0 | 0 | 0 | 0 |
| **Hippocampus** | 1 | 0 | 0 | 0 | 0 | 0 | 0 | 0 | 0 | 0 | 0 | 0 | 5 | 0 | 0 | 0 | 0 | 0 |
| **Hypothalamus** | 0 | 0 | 0 | 0 | 0 | 0 | 0 | 0 | 0 | 0 | 0 | 0 | 5 | 0 | 0 | 0 | 0 | 0 |
| **Insula** | 1 | 1 | 0 | 0 | 0 | 0 | 0 | 0 | 0 | 0 | 0 | 0 | 5 | 1 | 0 | 0 | 0 | 0 |
| **Lingual Gyrus** | 0 | 0 | 0 | 0 | 0 | 0 | 0 | 0 | 0 | 0 | 0 | 0 | 5 | 0 | 0 | 0 | 0 | 0 |
| **Occipital Cuneus** | 0 | 0 | 0 | 0 | 0 | 0 | 0 | 0 | 0 | 0 | 0 | 0 | 5 | 0 | 0 | 0 | 0 | 0 |
| **Occipital V2** | 0 | 0 | 0 | 0 | 0 | 0 | 0 | 0 | 0 | 0 | 0 | 0 | 5 | 1 | 0 | 0 | 0 | 0 |
| **Occipital V1** | 0 | 0 | 0 | 0 | 0 | 0 | 0 | 0 | 0 | 0 | 0 | 0 | 5 | 0 | 0 | 0 | 0 | 0 |
| **OFC** | 0 | 0 | 0 | 0 | 0 | 0 | 0 | 0 | 0 | 0 | 0 | 0 | 5 | 0 | 0 | 0 | 0 | 0 |
| **Parahippocampus** | 1 | 0 | 0 | 0 | 0 | 0 | 0 | 0 | 0 | 0 | 0 | 0 | 5 | 0 | 0 | 0 | 0 | 0 |
| **Parietal Inferior** | 0 | 0 | 0 | 0 | 0 | 0 | 0 | 0 | 0 | 0 | 0 | 0 | 5 | 0 | 0 | 0 | 0 | 0 |
| **Parietal Superior** | 0 | 0 | 0 | 0 | 0 | 0 | 0 | 0 | 0 | 0 | 0 | 0 | 5 | 0 | 0 | 0 | 0 | 0 |
| **Precuneus** | 0 | 0 | 0 | 0 | 0 | 0 | 0 | 0 | 0 | 0 | 0 | 0 | 5 | 0 | 0 | 0 | 0 | 0 |
| **Temporal Inferior** | 0 | 0 | 0 | 0 | 0 | 0 | 0 | 0 | 0 | 0 | 0 | 0 | 5 | 0 | 0 | 0 | 0 | 0 |
| **Temporal Middle** | 0 | 0 | 0 | 0 | 0 | 0 | 0 | 0 | 0 | 0 | 0 | 0 | 5 | 0 | 0 | 0 | 0 | 0 |
| **Temporal Pole** | 0 | 0 | 0 | 0 | 0 | 0 | 0 | 0 | 0 | 0 | 0 | 0 | 5 | 0 | 0 | 0 | 0 | 0 |
| **TemporalSuperior** | 0 | 0 | 0 | 0 | 0 | 0 | 0 | 0 | 0 | 0 | 0 | 0 | 5 | 0 | 0 | 0 | 0 | 0 |
| **Thalamus** | 1 | 0 | 0 | 0 | 0 | 0 | 0 | 0 | 0 | 0 | 0 | 0 | 5 | 0 | 0 | 0 | 0 | 0 |
| **NOSIGDIF** | 0 | 0 | 0 | 0 | 0 | 0 | 0 | 0 | 0 | 0 | 0 | 0 | 5 | 3 | 0 | 0 | 0 | 0 |

Supplementary Table 12e: Schizophrenia Spectrum Disorders

Numbers indicate how often each threshold was applied and how often each brain region was reportedly more activated in patients than controls. **ROI/SVC contrasts:** R1: uncorrected; R2: FDR corrected; R3: FWE corrected; R4: other unspecified multiple corrections. **Whole brain contrasts:** W1: uncorrected; W2: FDR corrected; W3: FWE corrected; W4: other unspecified multiple corrections; W5: Other.

| **Region** | **R1**  **Searched** | **R1**  **Activated** | **R2**  **Searched** | **R2**  **Activated** | **R3**  **Searched** | **R3**  **Activated** | **R4**  **Searched** | **R4**  **Activated** | **W1**  **Applied** | **W1**  **Activated** | **W2**  **Applied** | **W2**  **Activated** | **W3**  **Applied** | **W3**  **Activated** | **W4**  **Applied** | **W4**  **Activated** | **W5**  **Applied** | **W5**  **Activated** |
| --- | --- | --- | --- | --- | --- | --- | --- | --- | --- | --- | --- | --- | --- | --- | --- | --- | --- | --- |
| **Amygdala** | 0 | 0 | 6 | 1 | 2 | 0 | 2 | 0 | 0 | 0 | 0 | 0 | 2 | 0 | 2 | 0 | 0 | 0 |
| **Basal Ganglia** | 0 | 0 | 0 | 0 | 0 | 0 | 0 | 0 | 0 | 0 | 0 | 0 | 2 | 0 | 2 | 0 | 0 | 0 |
| **Brainstem** | 0 | 0 | 0 | 0 | 0 | 0 | 0 | 0 | 0 | 0 | 0 | 0 | 2 | 0 | 2 | 0 | 0 | 0 |
| **Cerebellum** | 0 | 0 | 0 | 0 | 0 | 0 | 0 | 0 | 0 | 0 | 0 | 0 | 2 | 0 | 2 | 0 | 0 | 0 |
| **Cingulate** | 0 | 0 | 3 | 0 | 0 | 0 | 0 | 0 | 0 | 0 | 0 | 0 | 2 | 0 | 2 | 0 | 0 | 0 |
| **Claustrum** | 0 | 0 | 0 | 0 | 0 | 0 | 0 | 0 | 0 | 0 | 0 | 0 | 2 | 0 | 2 | 0 | 0 | 0 |
| **Frontal dlPFC** | 0 | 0 | 2 | 0 | 0 | 0 | 0 | 0 | 0 | 0 | 0 | 0 | 2 | 0 | 2 | 0 | 0 | 0 |
| **Frontal dmPFC** | 0 | 0 | 4 | 1 | 0 | 0 | 0 | 0 | 0 | 0 | 0 | 0 | 2 | 0 | 2 | 0 | 0 | 0 |
| **Frontal vlPFC** | 0 | 0 | 2 | 0 | 0 | 0 | 0 | 0 | 0 | 0 | 0 | 0 | 2 | 0 | 2 | 0 | 0 | 0 |
| **Frontal vmPFC** | 0 | 0 | 8 | 1 | 0 | 0 | 0 | 0 | 0 | 0 | 0 | 0 | 2 | 0 | 2 | 0 | 0 | 0 |
| **Fusiform** | 0 | 0 | 0 | 0 | 0 | 0 | 0 | 0 | 0 | 0 | 0 | 0 | 2 | 0 | 2 | 0 | 0 | 0 |
| **Hippocampus** | 0 | 0 | 2 | 1 | 0 | 0 | 0 | 0 | 0 | 0 | 0 | 0 | 2 | 0 | 2 | 0 | 0 | 0 |
| **Hypothalamus** | 0 | 0 | 0 | 0 | 0 | 0 | 0 | 0 | 0 | 0 | 0 | 0 | 2 | 0 | 2 | 0 | 0 | 0 |
| **Insula** | 0 | 0 | 0 | 0 | 0 | 0 | 0 | 0 | 0 | 0 | 0 | 0 | 2 | 0 | 2 | 0 | 0 | 0 |
| **Lingual Gyrus** | 0 | 0 | 0 | 0 | 0 | 0 | 0 | 0 | 0 | 0 | 0 | 0 | 2 | 0 | 2 | 0 | 0 | 0 |
| **Occipital Cuneus** | 0 | 0 | 0 | 0 | 0 | 0 | 0 | 0 | 0 | 0 | 0 | 0 | 2 | 0 | 2 | 0 | 0 | 0 |
| **Occipital V2** | 0 | 0 | 0 | 0 | 0 | 0 | 0 | 0 | 0 | 0 | 0 | 0 | 2 | 0 | 2 | 0 | 0 | 0 |
| **Occipital V1** | 0 | 0 | 0 | 0 | 0 | 0 | 0 | 0 | 0 | 0 | 0 | 0 | 2 | 0 | 2 | 0 | 0 | 0 |
| **OFC** | 0 | 0 | 0 | 0 | 0 | 0 | 0 | 0 | 0 | 0 | 0 | 0 | 2 | 0 | 2 | 0 | 0 | 0 |
| **Parahippocampus** | 0 | 0 | 0 | 0 | 0 | 0 | 0 | 0 | 0 | 0 | 0 | 0 | 2 | 0 | 2 | 0 | 0 | 0 |
| **Parietal Inferior** | 0 | 0 | 2 | 1 | 0 | 0 | 0 | 0 | 0 | 0 | 0 | 0 | 2 | 0 | 2 | 0 | 0 | 0 |
| **Parietal Superior** | 0 | 0 | 0 | 0 | 0 | 0 | 0 | 0 | 0 | 0 | 0 | 0 | 2 | 0 | 2 | 0 | 0 | 0 |
| **Precuneus** | 0 | 0 | 0 | 0 | 0 | 0 | 0 | 0 | 0 | 0 | 0 | 0 | 2 | 0 | 2 | 0 | 0 | 0 |
| **Temporal Inferior** | 0 | 0 | 0 | 0 | 0 | 0 | 0 | 0 | 0 | 0 | 0 | 0 | 2 | 0 | 2 | 0 | 0 | 0 |
| **Temporal Middle** | 0 | 0 | 1 | 0 | 0 | 0 | 0 | 0 | 0 | 0 | 0 | 0 | 2 | 0 | 2 | 0 | 0 | 0 |
| **Temporal Pole** | 0 | 0 | 0 | 0 | 0 | 0 | 0 | 0 | 0 | 0 | 0 | 0 | 2 | 0 | 2 | 0 | 0 | 0 |
| **TemporalSuperior** | 0 | 0 | 0 | 0 | 0 | 0 | 0 | 0 | 0 | 0 | 0 | 0 | 2 | 0 | 2 | 0 | 0 | 0 |
| **Thalamus** | 0 | 0 | 0 | 0 | 0 | 0 | 0 | 0 | 0 | 0 | 0 | 0 | 2 | 0 | 2 | 0 | 0 | 0 |
| **NOSIGDIF** | 0 | 0 | 0 | 0 | 0 | 0 | 0 | 0 | 0 | 0 | 1 | 1 | 2 | 0 | 2 | 2 | 0 | 0 |

Supplementary Table 12f: Borderline Personality Disorder

Numbers indicate how often each threshold was applied and how often each brain region was reportedly more activated in patients than controls. **ROI/SVC contrasts:** R1: uncorrected; R2: FDR corrected; R3: FWE corrected; R4: other unspecified multiple corrections. **Whole brain contrasts:** W1: uncorrected; W2: FDR corrected; W3: FWE corrected; W4: other unspecified multiple corrections; W5: Other.

| **Region** | **R1**  **Searched** | **R1**  **Activated** | **R2**  **Searched** | **R2**  **Activated** | **R3**  **Searched** | **R3**  **Activated** | **R4**  **Searched** | **R4**  **Activated** | **W1**  **Applied** | **W1**  **Activated** | **W2**  **Applied** | **W2**  **Activated** | **W3**  **Applied** | **W3**  **Activated** | **W4**  **Applied** | **W4**  **Activated** | **W5**  **Applied** | **W5**  **Activated** |
| --- | --- | --- | --- | --- | --- | --- | --- | --- | --- | --- | --- | --- | --- | --- | --- | --- | --- | --- |
| **Amygdala** | 2 | 0 | 0 | 0 | 2 | 0 | 0 | 0 | 0 | 0 | 0 | 0 | 0 | 0 | 0 | 0 | 0 | 0 |
| **Basal Ganglia** | 0 | 0 | 0 | 0 | 0 | 0 | 0 | 0 | 0 | 0 | 0 | 0 | 0 | 0 | 0 | 0 | 0 | 0 |
| **Brainstem** | 0 | 0 | 0 | 0 | 0 | 0 | 0 | 0 | 0 | 0 | 0 | 0 | 0 | 0 | 0 | 0 | 0 | 0 |
| **Cerebellum** | 0 | 0 | 0 | 0 | 0 | 0 | 0 | 0 | 0 | 0 | 0 | 0 | 0 | 0 | 0 | 0 | 0 | 0 |
| **Cingulate** | 1 | 1 | 0 | 0 | 0 | 0 | 0 | 0 | 0 | 0 | 0 | 0 | 0 | 0 | 0 | 0 | 0 | 0 |
| **Claustrum** | 0 | 0 | 0 | 0 | 0 | 0 | 0 | 0 | 0 | 0 | 0 | 0 | 0 | 0 | 0 | 0 | 0 | 0 |
| **Frontal dlPFC** | 2 | 0 | 0 | 0 | 0 | 0 | 0 | 0 | 0 | 0 | 0 | 0 | 0 | 0 | 0 | 0 | 0 | 0 |
| **Frontal dmPFC** | 2 | 0 | 0 | 0 | 0 | 0 | 0 | 0 | 0 | 0 | 0 | 0 | 0 | 0 | 0 | 0 | 0 | 0 |
| **Frontal vlPFC** | 2 | 0 | 0 | 0 | 0 | 0 | 0 | 0 | 0 | 0 | 0 | 0 | 0 | 0 | 0 | 0 | 0 | 0 |
| **Frontal vmPFC** | 2 | 0 | 0 | 0 | 0 | 0 | 0 | 0 | 0 | 0 | 0 | 0 | 0 | 0 | 0 | 0 | 0 | 0 |
| **Fusiform** | 0 | 0 | 0 | 0 | 0 | 0 | 0 | 0 | 0 | 0 | 0 | 0 | 0 | 0 | 0 | 0 | 0 | 0 |
| **Hippocampus** | 2 | 0 | 0 | 0 | 0 | 0 | 0 | 0 | 0 | 0 | 0 | 0 | 0 | 0 | 0 | 0 | 0 | 0 |
| **Hypothalamus** | 0 | 0 | 0 | 0 | 0 | 0 | 0 | 0 | 0 | 0 | 0 | 0 | 0 | 0 | 0 | 0 | 0 | 0 |
| **Insula** | 0 | 0 | 0 | 0 | 0 | 0 | 0 | 0 | 0 | 0 | 0 | 0 | 0 | 0 | 0 | 0 | 0 | 0 |
| **Lingual Gyrus** | 0 | 0 | 0 | 0 | 0 | 0 | 0 | 0 | 0 | 0 | 0 | 0 | 0 | 0 | 0 | 0 | 0 | 0 |
| **Occipital Cuneus** | 0 | 0 | 0 | 0 | 0 | 0 | 0 | 0 | 0 | 0 | 0 | 0 | 0 | 0 | 0 | 0 | 0 | 0 |
| **Occipital V2** | 0 | 0 | 0 | 0 | 0 | 0 | 0 | 0 | 0 | 0 | 0 | 0 | 0 | 0 | 0 | 0 | 0 | 0 |
| **Occipital V1** | 0 | 0 | 0 | 0 | 0 | 0 | 0 | 0 | 0 | 0 | 0 | 0 | 0 | 0 | 0 | 0 | 0 | 0 |
| **OFC** | 0 | 0 | 0 | 0 | 0 | 0 | 0 | 0 | 0 | 0 | 0 | 0 | 0 | 0 | 0 | 0 | 0 | 0 |
| **Parahippocampus** | 0 | 0 | 0 | 0 | 0 | 0 | 0 | 0 | 0 | 0 | 0 | 0 | 0 | 0 | 0 | 0 | 0 | 0 |
| **Parietal Inferior** | 0 | 0 | 0 | 0 | 0 | 0 | 0 | 0 | 0 | 0 | 0 | 0 | 0 | 0 | 0 | 0 | 0 | 0 |
| **Parietal Superior** | 0 | 0 | 0 | 0 | 0 | 0 | 0 | 0 | 0 | 0 | 0 | 0 | 0 | 0 | 0 | 0 | 0 | 0 |
| **Precuneus** | 0 | 0 | 0 | 0 | 0 | 0 | 0 | 0 | 0 | 0 | 0 | 0 | 0 | 0 | 0 | 0 | 0 | 0 |
| **Temporal Inferior** | 0 | 0 | 0 | 0 | 0 | 0 | 0 | 0 | 0 | 0 | 0 | 0 | 0 | 0 | 0 | 0 | 0 | 0 |
| **Temporal Middle** | 0 | 0 | 0 | 0 | 0 | 0 | 0 | 0 | 0 | 0 | 0 | 0 | 0 | 0 | 0 | 0 | 0 | 0 |
| **Temporal Pole** | 0 | 0 | 0 | 0 | 0 | 0 | 0 | 0 | 0 | 0 | 0 | 0 | 0 | 0 | 0 | 0 | 0 | 0 |
| **TemporalSuperior** | 0 | 0 | 0 | 0 | 0 | 0 | 0 | 0 | 0 | 0 | 0 | 0 | 0 | 0 | 0 | 0 | 0 | 0 |
| **Thalamus** | 0 | 0 | 0 | 0 | 0 | 0 | 0 | 0 | 0 | 0 | 0 | 0 | 0 | 0 | 0 | 0 | 0 | 0 |
| **NOSIGDIF** | 0 | 0 | 0 | 0 | 0 | 0 | 0 | 0 | 0 | 0 | 0 | 0 | 0 | 0 | 0 | 0 | 0 | 0 |

Supplementary Table 12g: Autism Spectrum Disorder

Numbers indicate how often each threshold was applied and how often each brain region was reportedly more activated in patients than controls. **ROI/SVC contrasts:** R1: uncorrected; R2: FDR corrected; R3: FWE corrected; R4: other unspecified multiple corrections. **Whole brain contrasts:** W1: uncorrected; W2: FDR corrected; W3: FWE corrected; W4: other unspecified multiple corrections; W5: Other.

| **Region** | **R1**  **Searched** | **R1**  **Activated** | **R2**  **Searched** | **R2**  **Activated** | **R3**  **Searched** | **R3**  **Activated** | **R4**  **Searched** | **R4**  **Activated** | **W1**  **Applied** | **W1**  **Activated** | **W2**  **Applied** | **W2**  **Activated** | **W3**  **Applied** | **W3**  **Activated** | **W4**  **Applied** | **W4**  **Activated** | **W5**  **Applied** | **W5**  **Activated** |
| --- | --- | --- | --- | --- | --- | --- | --- | --- | --- | --- | --- | --- | --- | --- | --- | --- | --- | --- |
| **Amygdala** | 3 | 0 | 0 | 0 | 0 | 0 | 2 | 0 | 1 | 0 | 0 | 0 | 0 | 0 | 1 | 0 | 1 | 0 |
| **Basal Ganglia** | 0 | 0 | 0 | 0 | 0 | 0 | 0 | 0 | 1 | 0 | 0 | 0 | 0 | 0 | 1 | 0 | 1 | 0 |
| **Brainstem** | 0 | 0 | 0 | 0 | 0 | 0 | 0 | 0 | 1 | 0 | 0 | 0 | 0 | 0 | 1 | 0 | 1 | 0 |
| **Cerebellum** | 0 | 0 | 0 | 0 | 0 | 0 | 0 | 0 | 1 | 0 | 0 | 0 | 0 | 0 | 1 | 1 | 1 | 0 |
| **Cingulate** | 0 | 0 | 0 | 0 | 0 | 0 | 0 | 0 | 1 | 0 | 0 | 0 | 0 | 0 | 1 | 0 | 1 | 0 |
| **Claustrum** | 0 | 0 | 0 | 0 | 0 | 0 | 0 | 0 | 1 | 0 | 0 | 0 | 0 | 0 | 1 | 0 | 1 | 0 |
| **Frontal dlPFC** | 2 | 0 | 0 | 0 | 0 | 0 | 0 | 0 | 1 | 1 | 0 | 0 | 0 | 0 | 1 | 0 | 1 | 0 |
| **Frontal dmPFC** | 2 | 0 | 0 | 0 | 0 | 0 | 0 | 0 | 1 | 0 | 0 | 0 | 0 | 0 | 1 | 0 | 1 | 0 |
| **Frontal vlPFC** | 2 | 0 | 0 | 0 | 0 | 0 | 2 | 0 | 1 | 0 | 0 | 0 | 0 | 0 | 1 | 0 | 1 | 0 |
| **Frontal vmPFC** | 2 | 0 | 0 | 0 | 0 | 0 | 0 | 0 | 1 | 0 | 0 | 0 | 0 | 0 | 1 | 0 | 1 | 0 |
| **Fusiform** | 2 | 0 | 0 | 0 | 0 | 0 | 2 | 0 | 1 | 0 | 0 | 0 | 0 | 0 | 1 | 0 | 1 | 0 |
| **Hippocampus** | 0 | 0 | 0 | 0 | 0 | 0 | 0 | 0 | 1 | 0 | 0 | 0 | 0 | 0 | 1 | 0 | 1 | 0 |
| **Hypothalamus** | 0 | 0 | 0 | 0 | 0 | 0 | 0 | 0 | 1 | 0 | 0 | 0 | 0 | 0 | 1 | 0 | 1 | 0 |
| **Insula** | 0 | 0 | 0 | 0 | 0 | 0 | 0 | 0 | 1 | 0 | 0 | 0 | 0 | 0 | 1 | 0 | 1 | 0 |
| **Lingual Gyrus** | 0 | 0 | 0 | 0 | 0 | 0 | 0 | 0 | 1 | 0 | 0 | 0 | 0 | 0 | 1 | 1 | 1 | 0 |
| **Occipital Cuneus** | 0 | 0 | 0 | 0 | 0 | 0 | 0 | 0 | 1 | 0 | 0 | 0 | 0 | 0 | 1 | 0 | 1 | 0 |
| **Occipital V2** | 0 | 0 | 0 | 0 | 0 | 0 | 1 | 0 | 1 | 0 | 0 | 0 | 0 | 0 | 1 | 0 | 1 | 0 |
| **Occipital V1** | 0 | 0 | 0 | 0 | 0 | 0 | 1 | 0 | 1 | 0 | 0 | 0 | 0 | 0 | 1 | 1 | 1 | 0 |
| **OFC** | 0 | 0 | 0 | 0 | 0 | 0 | 0 | 0 | 1 | 0 | 0 | 0 | 0 | 0 | 1 | 0 | 1 | 0 |
| **Parahippocampus** | 0 | 0 | 0 | 0 | 0 | 0 | 0 | 0 | 1 | 0 | 0 | 0 | 0 | 0 | 1 | 0 | 1 | 0 |
| **Parietal Inferior** | 0 | 0 | 0 | 0 | 0 | 0 | 0 | 0 | 1 | 0 | 0 | 0 | 0 | 0 | 1 | 0 | 1 | 0 |
| **Parietal Superior** | 0 | 0 | 0 | 0 | 0 | 0 | 0 | 0 | 1 | 0 | 0 | 0 | 0 | 0 | 1 | 0 | 1 | 0 |
| **Precuneus** | 2 | 2 | 0 | 0 | 0 | 0 | 0 | 0 | 1 | 0 | 0 | 0 | 0 | 0 | 1 | 0 | 1 | 0 |
| **Temporal Inferior** | 0 | 0 | 0 | 0 | 0 | 0 | 1 | 0 | 1 | 0 | 0 | 0 | 0 | 0 | 1 | 0 | 1 | 0 |
| **Temporal Middle** | 0 | 0 | 0 | 0 | 0 | 0 | 2 | 0 | 1 | 0 | 0 | 0 | 0 | 0 | 1 | 0 | 1 | 0 |
| **Temporal Pole** | 0 | 0 | 0 | 0 | 0 | 0 | 0 | 0 | 1 | 0 | 0 | 0 | 0 | 0 | 1 | 0 | 1 | 0 |
| **TemporalSuperior** | 0 | 0 | 0 | 0 | 0 | 0 | 2 | 0 | 1 | 0 | 0 | 0 | 0 | 0 | 1 | 0 | 1 | 0 |
| **Thalamus** | 0 | 0 | 0 | 0 | 0 | 0 | 0 | 0 | 1 | 0 | 0 | 0 | 0 | 0 | 1 | 0 | 1 | 0 |
| **NOSIGDIF** | 0 | 0 | 0 | 0 | 0 | 0 | 0 | 0 | 1 | 0 | 0 | 0 | 0 | 0 | 1 | 0 | 1 | 1 |

Supplementary Table 12h: Attention Deficit Hyperactivity Disorder

Numbers indicate how often each threshold was applied and how often each brain region was reportedly more activated in patients than controls. **ROI/SVC contrasts:** R1: uncorrected; R2: FDR corrected; R3: FWE corrected; R4: other unspecified multiple corrections. **Whole brain contrasts:** W1: uncorrected; W2: FDR corrected; W3: FWE corrected; W4: other unspecified multiple corrections; W5: Other.

| **Region** | **R1**  **Searched** | **R1**  **Activated** | **R2**  **Searched** | **R2**  **Activated** | **R3**  **Searched** | **R3**  **Activated** | **R4**  **Searched** | **R4**  **Activated** | **W1**  **Applied** | **W1**  **Activated** | **W2**  **Applied** | **W2**  **Activated** | **W3**  **Applied** | **W3**  **Activated** | **W4**  **Applied** | **W4**  **Activated** | **W5**  **Applied** | **W5**  **Activated** |
| --- | --- | --- | --- | --- | --- | --- | --- | --- | --- | --- | --- | --- | --- | --- | --- | --- | --- | --- |
| **Amygdala** | 2 | 0 | 0 | 0 | 2 | 0 | 0 | 0 | 0 | 0 | 0 | 0 | 2 | 0 | 0 | 0 | 1 | 0 |
| **Basal Ganglia** | 0 | 0 | 0 | 0 | 0 | 0 | 0 | 0 | 0 | 0 | 0 | 0 | 2 | 0 | 0 | 0 | 1 | 0 |
| **Brainstem** | 0 | 0 | 0 | 0 | 0 | 0 | 0 | 0 | 0 | 0 | 0 | 0 | 2 | 0 | 0 | 0 | 1 | 0 |
| **Cerebellum** | 0 | 0 | 0 | 0 | 0 | 0 | 0 | 0 | 0 | 0 | 0 | 0 | 2 | 0 | 0 | 0 | 1 | 1 |
| **Cingulate** | 0 | 0 | 0 | 0 | 0 | 0 | 0 | 0 | 0 | 0 | 0 | 0 | 2 | 0 | 0 | 0 | 1 | 4 |
| **Claustrum** | 0 | 0 | 0 | 0 | 0 | 0 | 0 | 0 | 0 | 0 | 0 | 0 | 2 | 0 | 0 | 0 | 1 | 0 |
| **Frontal dlPFC** | 0 | 0 | 0 | 0 | 0 | 0 | 0 | 0 | 0 | 0 | 0 | 0 | 2 | 0 | 0 | 0 | 1 | 1 |
| **Frontal dmPFC** | 0 | 0 | 0 | 0 | 0 | 0 | 0 | 0 | 0 | 0 | 0 | 0 | 2 | 1 | 0 | 0 | 1 | 0 |
| **Frontal vlPFC** | 0 | 0 | 0 | 0 | 0 | 0 | 0 | 0 | 0 | 0 | 0 | 0 | 2 | 0 | 0 | 0 | 1 | 0 |
| **Frontal vmPFC** | 0 | 0 | 0 | 0 | 0 | 0 | 0 | 0 | 0 | 0 | 0 | 0 | 2 | 0 | 0 | 0 | 1 | 2 |
| **Fusiform** | 0 | 0 | 0 | 0 | 0 | 0 | 0 | 0 | 0 | 0 | 0 | 0 | 2 | 0 | 0 | 0 | 1 | 0 |
| **Hippocampus** | 0 | 0 | 0 | 0 | 0 | 0 | 0 | 0 | 0 | 0 | 0 | 0 | 2 | 0 | 0 | 0 | 1 | 0 |
| **Hypothalamus** | 0 | 0 | 0 | 0 | 0 | 0 | 0 | 0 | 0 | 0 | 0 | 0 | 2 | 0 | 0 | 0 | 1 | 0 |
| **Insula** | 0 | 0 | 0 | 0 | 0 | 0 | 0 | 0 | 0 | 0 | 0 | 0 | 2 | 0 | 0 | 0 | 1 | 0 |
| **Lingual Gyrus** | 0 | 0 | 0 | 0 | 0 | 0 | 0 | 0 | 0 | 0 | 0 | 0 | 2 | 0 | 0 | 0 | 1 | 0 |
| **Occipital Cuneus** | 0 | 0 | 0 | 0 | 0 | 0 | 0 | 0 | 0 | 0 | 0 | 0 | 2 | 0 | 0 | 0 | 1 | 0 |
| **Occipital V2** | 0 | 0 | 0 | 0 | 0 | 0 | 0 | 0 | 0 | 0 | 0 | 0 | 2 | 2 | 0 | 0 | 1 | 0 |
| **Occipital V1** | 0 | 0 | 0 | 0 | 0 | 0 | 0 | 0 | 0 | 0 | 0 | 0 | 2 | 0 | 0 | 0 | 1 | 0 |
| **OFC** | 0 | 0 | 0 | 0 | 0 | 0 | 0 | 0 | 0 | 0 | 0 | 0 | 2 | 0 | 0 | 0 | 1 | 0 |
| **Parahippocampus** | 0 | 0 | 0 | 0 | 0 | 0 | 0 | 0 | 0 | 0 | 0 | 0 | 2 | 0 | 0 | 0 | 1 | 0 |
| **Parietal Inferior** | 0 | 0 | 0 | 0 | 0 | 0 | 0 | 0 | 0 | 0 | 0 | 0 | 2 | 0 | 0 | 0 | 1 | 1 |
| **Parietal Superior** | 0 | 0 | 0 | 0 | 0 | 0 | 0 | 0 | 0 | 0 | 0 | 0 | 2 | 0 | 0 | 0 | 1 | 0 |
| **Precuneus** | 0 | 0 | 0 | 0 | 0 | 0 | 0 | 0 | 0 | 0 | 0 | 0 | 2 | 1 | 0 | 0 | 1 | 0 |
| **Temporal Inferior** | 0 | 0 | 0 | 0 | 0 | 0 | 0 | 0 | 0 | 0 | 0 | 0 | 2 | 0 | 0 | 0 | 1 | 0 |
| **Temporal Middle** | 0 | 0 | 0 | 0 | 0 | 0 | 0 | 0 | 0 | 0 | 0 | 0 | 2 | 0 | 0 | 0 | 1 | 1 |
| **Temporal Pole** | 0 | 0 | 0 | 0 | 0 | 0 | 0 | 0 | 0 | 0 | 0 | 0 | 2 | 0 | 0 | 0 | 1 | 0 |
| **TemporalSuperior** | 0 | 0 | 0 | 0 | 0 | 0 | 0 | 0 | 0 | 0 | 0 | 0 | 2 | 0 | 0 | 0 | 1 | 0 |
| **Thalamus** | 0 | 0 | 0 | 0 | 0 | 0 | 0 | 0 | 0 | 0 | 0 | 0 | 2 | 0 | 0 | 0 | 1 | 0 |
| **NOSIGDIF** | 0 | 0 | 0 | 0 | 0 | 0 | 0 | 0 | 0 | 0 | 0 | 0 | 2 | 1 | 0 | 0 | 1 | 0 |

Supplementary Table 12i: Substance Use Disorder

Numbers indicate how often each threshold was applied and how often each brain region was reportedly more activated in patients than controls. **ROI/SVC contrasts:** R1: uncorrected; R2: FDR corrected; R3: FWE corrected; R4: other unspecified multiple corrections. **Whole brain contrasts:** W1: uncorrected; W2: FDR corrected; W3: FWE corrected; W4: other unspecified multiple corrections; W5: Other.

| **Region** | **R1**  **Searched** | **R1**  **Activated** | **R2**  **Searched** | **R2**  **Activated** | **R3**  **Searched** | **R3**  **Activated** | **R4**  **Searched** | **R4**  **Activated** | **W1**  **Applied** | **W1**  **Activated** | **W2**  **Applied** | **W2**  **Activated** | **W3**  **Applied** | **W3**  **Activated** | **W4**  **Applied** | **W4**  **Activated** | **W5**  **Applied** | **W5**  **Activated** |
| --- | --- | --- | --- | --- | --- | --- | --- | --- | --- | --- | --- | --- | --- | --- | --- | --- | --- | --- |
| **Amygdala** | 0 | 0 | 0 | 0 | 0 | 0 | 0 | 0 | 1 | 0 | 0 | 0 | 2 | 0 | 0 | 0 | 1 | 0 |
| **Basal Ganglia** | 0 | 0 | 0 | 0 | 0 | 0 | 0 | 0 | 0 | 0 | 0 | 0 | 2 | 0 | 0 | 0 | 1 | 0 |
| **Brainstem** | 0 | 0 | 0 | 0 | 0 | 0 | 0 | 0 | 0 | 0 | 0 | 0 | 2 | 0 | 0 | 0 | 1 | 0 |
| **Cerebellum** | 0 | 0 | 0 | 0 | 0 | 0 | 0 | 0 | 0 | 0 | 0 | 0 | 2 | 0 | 0 | 0 | 1 | 1 |
| **Cingulate** | 0 | 0 | 0 | 0 | 0 | 0 | 0 | 0 | 2 | 2 | 0 | 0 | 2 | 0 | 0 | 0 | 1 | 4 |
| **Claustrum** | 0 | 0 | 0 | 0 | 0 | 0 | 0 | 0 | 0 | 0 | 0 | 0 | 2 | 0 | 0 | 0 | 1 | 0 |
| **Frontal dlPFC** | 0 | 0 | 0 | 0 | 0 | 0 | 0 | 0 | 0 | 0 | 0 | 0 | 2 | 0 | 0 | 0 | 1 | 1 |
| **Frontal dmPFC** | 0 | 0 | 0 | 0 | 0 | 0 | 0 | 0 | 0 | 0 | 0 | 0 | 2 | 0 | 0 | 0 | 1 | 0 |
| **Frontal vlPFC** | 0 | 0 | 0 | 0 | 0 | 0 | 0 | 0 | 0 | 0 | 0 | 0 | 2 | 0 | 0 | 0 | 1 | 0 |
| **Frontal vmPFC** | 0 | 0 | 0 | 0 | 0 | 0 | 0 | 0 | 0 | 0 | 0 | 0 | 2 | 0 | 0 | 0 | 1 | 2 |
| **Fusiform** | 0 | 0 | 0 | 0 | 0 | 0 | 0 | 0 | 0 | 0 | 0 | 0 | 2 | 0 | 0 | 0 | 1 | 0 |
| **Hippocampus** | 0 | 0 | 0 | 0 | 0 | 0 | 0 | 0 | 0 | 0 | 0 | 0 | 2 | 0 | 0 | 0 | 1 | 0 |
| **Hypothalamus** | 0 | 0 | 0 | 0 | 0 | 0 | 0 | 0 | 0 | 0 | 0 | 0 | 2 | 0 | 0 | 0 | 1 | 0 |
| **Insula** | 0 | 0 | 0 | 0 | 0 | 0 | 0 | 0 | 0 | 0 | 0 | 0 | 2 | 0 | 0 | 0 | 1 | 0 |
| **Lingual Gyrus** | 0 | 0 | 0 | 0 | 0 | 0 | 0 | 0 | 0 | 0 | 0 | 0 | 2 | 0 | 0 | 0 | 1 | 0 |
| **Occipital Cuneus** | 0 | 0 | 0 | 0 | 0 | 0 | 0 | 0 | 0 | 0 | 0 | 0 | 2 | 0 | 0 | 0 | 1 | 0 |
| **Occipital V2** | 0 | 0 | 0 | 0 | 0 | 0 | 0 | 0 | 0 | 0 | 0 | 0 | 2 | 0 | 0 | 0 | 1 | 0 |
| **Occipital V1** | 0 | 0 | 0 | 0 | 0 | 0 | 0 | 0 | 0 | 0 | 0 | 0 | 2 | 0 | 0 | 0 | 1 | 0 |
| **OFC** | 0 | 0 | 0 | 0 | 0 | 0 | 0 | 0 | 0 | 0 | 0 | 0 | 2 | 0 | 0 | 0 | 1 | 0 |
| **Parahippocampus** | 0 | 0 | 0 | 0 | 0 | 0 | 0 | 0 | 0 | 0 | 0 | 0 | 2 | 0 | 0 | 0 | 1 | 0 |
| **Parietal Inferior** | 0 | 0 | 0 | 0 | 0 | 0 | 0 | 0 | 0 | 0 | 0 | 0 | 2 | 0 | 0 | 0 | 1 | 1 |
| **Parietal Superior** | 0 | 0 | 0 | 0 | 0 | 0 | 0 | 0 | 0 | 0 | 0 | 0 | 2 | 0 | 0 | 0 | 1 | 0 |
| **Precuneus** | 0 | 0 | 0 | 0 | 0 | 0 | 0 | 0 | 0 | 0 | 0 | 0 | 2 | 0 | 0 | 0 | 1 | 0 |
| **Temporal Inferior** | 0 | 0 | 0 | 0 | 0 | 0 | 0 | 0 | 0 | 0 | 0 | 0 | 2 | 0 | 0 | 0 | 1 | 0 |
| **Temporal Middle** | 0 | 0 | 0 | 0 | 0 | 0 | 0 | 0 | 0 | 0 | 0 | 0 | 2 | 0 | 0 | 0 | 1 | 1 |
| **Temporal Pole** | 0 | 0 | 0 | 0 | 0 | 0 | 0 | 0 | 0 | 0 | 0 | 0 | 2 | 0 | 0 | 0 | 1 | 0 |
| **TemporalSuperior** | 0 | 0 | 0 | 0 | 0 | 0 | 0 | 0 | 0 | 0 | 0 | 0 | 2 | 0 | 0 | 0 | 1 | 0 |
| **Thalamus** | 0 | 0 | 0 | 0 | 0 | 0 | 0 | 0 | 0 | 0 | 0 | 0 | 2 | 0 | 0 | 0 | 1 | 0 |
| **NOSIGDIF** | 0 | 0 | 0 | 0 | 0 | 0 | 0 | 0 | 0 | 0 | 0 | 0 | 2 | 2 | 0 | 0 | 1 | 0 |

Supplementary Table 12j: Gender Dysphoria

Numbers indicate how often each threshold was applied and how often each brain region was reportedly more activated in patients than controls. **ROI/SVC contrasts:** R1: uncorrected; R2: FDR corrected; R3: FWE corrected; R4: other unspecified multiple corrections. **Whole brain contrasts:** W1: uncorrected; W2: FDR corrected; W3: FWE corrected; W4: other unspecified multiple corrections; W5: Other.

| **Region** | **R1**  **Searched** | **R1**  **Activated** | **R2**  **Searched** | **R2**  **Activated** | **R3**  **Searched** | **R3**  **Activated** | **R4**  **Searched** | **R4**  **Activated** | **W1**  **Applied** | **W1**  **Activated** | **W2**  **Applied** | **W2**  **Activated** | **W3**  **Applied** | **W3**  **Activated** | **W4**  **Applied** | **W4**  **Activated** | **W5**  **Applied** | **W5**  **Activated** |
| --- | --- | --- | --- | --- | --- | --- | --- | --- | --- | --- | --- | --- | --- | --- | --- | --- | --- | --- |
| **Amygdala** | 0 | 0 | 0 | 0 | 0 | 0 | 0 | 0 | 0 | 0 | 0 | 0 | 0 | 0 | 0 | 0 | 0 | 0 |
| **Basal Ganglia** | 0 | 0 | 0 | 0 | 0 | 0 | 0 | 0 | 0 | 0 | 0 | 0 | 0 | 0 | 0 | 0 | 0 | 0 |
| **Brainstem** | 0 | 0 | 0 | 0 | 0 | 0 | 0 | 0 | 0 | 0 | 0 | 0 | 0 | 0 | 0 | 0 | 0 | 0 |
| **Cerebellum** | 0 | 0 | 0 | 0 | 0 | 0 | 0 | 0 | 0 | 0 | 0 | 0 | 0 | 0 | 0 | 0 | 0 | 0 |
| **Cingulate** | 0 | 0 | 0 | 0 | 0 | 0 | 0 | 0 | 0 | 0 | 0 | 0 | 0 | 0 | 0 | 0 | 0 | 0 |
| **Claustrum** | 0 | 0 | 0 | 0 | 0 | 0 | 0 | 0 | 0 | 0 | 0 | 0 | 0 | 0 | 0 | 0 | 0 | 0 |
| **Frontal dlPFC** | 0 | 0 | 0 | 0 | 0 | 0 | 0 | 0 | 0 | 0 | 0 | 0 | 0 | 0 | 0 | 0 | 0 | 0 |
| **Frontal dmPFC** | 0 | 0 | 0 | 0 | 0 | 0 | 0 | 0 | 0 | 0 | 0 | 0 | 0 | 0 | 0 | 0 | 0 | 0 |
| **Frontal vlPFC** | 0 | 0 | 0 | 0 | 0 | 0 | 0 | 0 | 0 | 0 | 0 | 0 | 0 | 0 | 0 | 0 | 0 | 0 |
| **Frontal vmPFC** | 0 | 0 | 0 | 0 | 0 | 0 | 0 | 0 | 0 | 0 | 0 | 0 | 0 | 0 | 0 | 0 | 0 | 0 |
| **Fusiform** | 0 | 0 | 0 | 0 | 0 | 0 | 0 | 0 | 0 | 0 | 0 | 0 | 0 | 0 | 0 | 0 | 0 | 0 |
| **Hippocampus** | 0 | 0 | 0 | 0 | 0 | 0 | 0 | 0 | 0 | 0 | 0 | 0 | 0 | 0 | 0 | 0 | 0 | 0 |
| **Hypothalamus** | 0 | 0 | 0 | 0 | 0 | 0 | 0 | 0 | 0 | 0 | 0 | 0 | 0 | 0 | 0 | 0 | 0 | 0 |
| **Insula** | 0 | 0 | 0 | 0 | 0 | 0 | 0 | 0 | 0 | 0 | 0 | 0 | 0 | 0 | 0 | 0 | 0 | 0 |
| **Lingual Gyrus** | 0 | 0 | 0 | 0 | 0 | 0 | 0 | 0 | 0 | 0 | 0 | 0 | 0 | 0 | 0 | 0 | 0 | 0 |
| **Occipital Cuneus** | 0 | 0 | 0 | 0 | 0 | 0 | 0 | 0 | 0 | 0 | 0 | 0 | 0 | 0 | 0 | 0 | 0 | 0 |
| **Occipital V2** | 0 | 0 | 0 | 0 | 0 | 0 | 0 | 0 | 0 | 0 | 0 | 0 | 0 | 0 | 0 | 0 | 0 | 0 |
| **Occipital V1** | 0 | 0 | 0 | 0 | 0 | 0 | 0 | 0 | 0 | 0 | 0 | 0 | 0 | 0 | 0 | 0 | 0 | 0 |
| **OFC** | 0 | 0 | 0 | 0 | 0 | 0 | 0 | 0 | 0 | 0 | 0 | 0 | 0 | 0 | 0 | 0 | 0 | 0 |
| **Parahippocampus** | 0 | 0 | 0 | 0 | 0 | 0 | 0 | 0 | 0 | 0 | 0 | 0 | 0 | 0 | 0 | 0 | 0 | 0 |
| **Parietal Inferior** | 0 | 0 | 0 | 0 | 0 | 0 | 0 | 0 | 0 | 0 | 0 | 0 | 0 | 0 | 0 | 0 | 0 | 0 |
| **Parietal Superior** | 0 | 0 | 0 | 0 | 0 | 0 | 0 | 0 | 0 | 0 | 0 | 0 | 0 | 0 | 0 | 0 | 0 | 0 |
| **Precuneus** | 0 | 0 | 0 | 0 | 0 | 0 | 0 | 0 | 0 | 0 | 0 | 0 | 0 | 0 | 0 | 0 | 0 | 0 |
| **Temporal Inferior** | 0 | 0 | 0 | 0 | 0 | 0 | 0 | 0 | 0 | 0 | 0 | 0 | 0 | 0 | 0 | 0 | 0 | 0 |
| **Temporal Middle** | 0 | 0 | 0 | 0 | 0 | 0 | 0 | 0 | 0 | 0 | 0 | 0 | 0 | 0 | 0 | 0 | 0 | 0 |
| **Temporal Pole** | 0 | 0 | 0 | 0 | 0 | 0 | 0 | 0 | 0 | 0 | 0 | 0 | 0 | 0 | 0 | 0 | 0 | 0 |
| **TemporalSuperior** | 0 | 0 | 0 | 0 | 0 | 0 | 0 | 0 | 0 | 0 | 0 | 0 | 0 | 0 | 0 | 0 | 0 | 0 |
| **Thalamus** | 0 | 0 | 0 | 0 | 0 | 0 | 0 | 0 | 0 | 0 | 0 | 0 | 0 | 0 | 0 | 0 | 0 | 0 |
| **NOSIGDIF** | 0 | 0 | 0 | 0 | 0 | 0 | 0 | 0 | 0 | 0 | 0 | 0 | 1 | 1 | 0 | 0 | 0 | 0 |

Supplementary Table 12k: Premenstrual Dysphoric Disorder

Numbers indicate how often each threshold was applied and how often each brain region was reportedly more activated in patients than controls. **ROI/SVC contrasts:** R1: uncorrected; R2: FDR corrected; R3: FWE corrected; R4: other unspecified multiple corrections. **Whole brain contrasts:** W1: uncorrected; W2: FDR corrected; W3: FWE corrected; W4: other unspecified multiple corrections; W5: Other.

| **Region** | **R1**  **Searched** | **R1**  **Activated** | **R2**  **Searched** | **R2**  **Activated** | **R3**  **Searched** | **R3**  **Activated** | **R4**  **Searched** | **R4**  **Activated** | **W1**  **Applied** | **W1**  **Activated** | **W2**  **Applied** | **W2**  **Activated** | **W3**  **Applied** | **W3**  **Activated** | **W4**  **Applied** | **W4**  **Activated** | **W5**  **Applied** | **W5**  **Activated** |
| --- | --- | --- | --- | --- | --- | --- | --- | --- | --- | --- | --- | --- | --- | --- | --- | --- | --- | --- |
| **Amygdala** | 0 | 0 | 0 | 0 | 0 | 0 | 0 | 0 | 0 | 0 | 0 | 0 | 2 | 0 | 0 | 0 | 0 | 0 |
| **Basal Ganglia** | 0 | 0 | 0 | 0 | 0 | 0 | 0 | 0 | 0 | 0 | 0 | 0 | 2 | 1 | 0 | 0 | 0 | 0 |
| **Brainstem** | 0 | 0 | 0 | 0 | 0 | 0 | 0 | 0 | 0 | 0 | 0 | 0 | 2 | 0 | 0 | 0 | 0 | 0 |
| **Cerebellum** | 0 | 0 | 0 | 0 | 0 | 0 | 0 | 0 | 0 | 0 | 0 | 0 | 2 | 0 | 0 | 0 | 0 | 0 |
| **Cingulate** | 0 | 0 | 0 | 0 | 0 | 0 | 0 | 0 | 0 | 0 | 0 | 0 | 2 | 2 | 0 | 0 | 0 | 0 |
| **Claustrum** | 0 | 0 | 0 | 0 | 0 | 0 | 0 | 0 | 0 | 0 | 0 | 0 | 2 | 0 | 0 | 0 | 0 | 0 |
| **Frontal dlPFC** | 0 | 0 | 0 | 0 | 0 | 0 | 0 | 0 | 0 | 0 | 0 | 0 | 2 | 2 | 0 | 0 | 0 | 0 |
| **Frontal dmPFC** | 0 | 0 | 0 | 0 | 0 | 0 | 0 | 0 | 0 | 0 | 0 | 0 | 2 | 0 | 0 | 0 | 0 | 0 |
| **Frontal vlPFC** | 0 | 0 | 0 | 0 | 0 | 0 | 0 | 0 | 0 | 0 | 0 | 0 | 2 | 0 | 0 | 0 | 0 | 0 |
| **Frontal vmPFC** | 0 | 0 | 0 | 0 | 0 | 0 | 0 | 0 | 0 | 0 | 0 | 0 | 2 | 0 | 0 | 0 | 0 | 0 |
| **Fusiform** | 0 | 0 | 0 | 0 | 0 | 0 | 0 | 0 | 0 | 0 | 0 | 0 | 2 | 0 | 0 | 0 | 0 | 0 |
| **Hippocampus** | 0 | 0 | 0 | 0 | 0 | 0 | 0 | 0 | 0 | 0 | 0 | 0 | 2 | 0 | 0 | 0 | 0 | 0 |
| **Hypothalamus** | 0 | 0 | 0 | 0 | 0 | 0 | 0 | 0 | 0 | 0 | 0 | 0 | 2 | 0 | 0 | 0 | 0 | 0 |
| **Insula** | 0 | 0 | 0 | 0 | 0 | 0 | 0 | 0 | 0 | 0 | 0 | 0 | 2 | 2 | 0 | 0 | 0 | 0 |
| **Lingual Gyrus** | 0 | 0 | 0 | 0 | 0 | 0 | 0 | 0 | 0 | 0 | 0 | 0 | 2 | 0 | 0 | 0 | 0 | 0 |
| **Occipital Cuneus** | 0 | 0 | 0 | 0 | 0 | 0 | 0 | 0 | 0 | 0 | 0 | 0 | 2 | 0 | 0 | 0 | 0 | 0 |
| **Occipital V2** | 0 | 0 | 0 | 0 | 0 | 0 | 0 | 0 | 0 | 0 | 0 | 0 | 2 | 0 | 0 | 0 | 0 | 0 |
| **Occipital V1** | 0 | 0 | 0 | 0 | 0 | 0 | 0 | 0 | 0 | 0 | 0 | 0 | 2 | 0 | 0 | 0 | 0 | 0 |
| **OFC** | 0 | 0 | 0 | 0 | 0 | 0 | 0 | 0 | 0 | 0 | 0 | 0 | 2 | 0 | 0 | 0 | 0 | 0 |
| **Parahippocampus** | 0 | 0 | 0 | 0 | 0 | 0 | 0 | 0 | 0 | 0 | 0 | 0 | 2 | 0 | 0 | 0 | 0 | 0 |
| **Parietal Inferior** | 0 | 0 | 0 | 0 | 0 | 0 | 0 | 0 | 0 | 0 | 0 | 0 | 2 | 0 | 0 | 0 | 0 | 0 |
| **Parietal Superior** | 0 | 0 | 0 | 0 | 0 | 0 | 0 | 0 | 0 | 0 | 0 | 0 | 2 | 0 | 0 | 0 | 0 | 0 |
| **Precuneus** | 0 | 0 | 0 | 0 | 0 | 0 | 0 | 0 | 0 | 0 | 0 | 0 | 2 | 1 | 0 | 0 | 0 | 0 |
| **Temporal Inferior** | 0 | 0 | 0 | 0 | 0 | 0 | 0 | 0 | 0 | 0 | 0 | 0 | 2 | 0 | 0 | 0 | 0 | 0 |
| **Temporal Middle** | 0 | 0 | 0 | 0 | 0 | 0 | 0 | 0 | 0 | 0 | 0 | 0 | 2 | 0 | 0 | 0 | 0 | 0 |
| **Temporal Pole** | 0 | 0 | 0 | 0 | 0 | 0 | 0 | 0 | 0 | 0 | 0 | 0 | 2 | 0 | 0 | 0 | 0 | 0 |
| **TemporalSuperior** | 0 | 0 | 0 | 0 | 0 | 0 | 0 | 0 | 0 | 0 | 0 | 0 | 2 | 0 | 0 | 0 | 0 | 0 |
| **Thalamus** | 0 | 0 | 0 | 0 | 0 | 0 | 0 | 0 | 0 | 0 | 0 | 0 | 2 | 0 | 0 | 0 | 0 | 0 |
| **NOSIGDIF** | 0 | 0 | 0 | 0 | 0 | 0 | 0 | 0 | 0 | 0 | 0 | 0 | 2 | 1 | 0 | 0 | 0 | 0 |

Supplementary Table 12l: Body Dysmorphic Disorder

Numbers indicate how often each threshold was applied and how often each brain region was reportedly more activated in patients than controls. **ROI/SVC contrasts:** R1: uncorrected; R2: FDR corrected; R3: FWE corrected; R4: other unspecified multiple corrections. **Whole brain contrasts:** W1: uncorrected; W2: FDR corrected; W3: FWE corrected; W4: other unspecified multiple corrections; W5: Other.

| **Region** | **R1**  **Searched** | **R1**  **Activated** | **R2**  **Searched** | **R2**  **Activated** | **R3**  **Searched** | **R3**  **Activated** | **R4**  **Searched** | **R4**  **Activated** | **W1**  **Applied** | **W1**  **Activated** | **W2**  **Applied** | **W2**  **Activated** | **W3**  **Applied** | **W3**  **Activated** | **W4**  **Applied** | **W4**  **Activated** | **W5**  **Applied** | **W5**  **Activated** |
| --- | --- | --- | --- | --- | --- | --- | --- | --- | --- | --- | --- | --- | --- | --- | --- | --- | --- | --- |
| **Amygdala** | 0 | 0 | 0 | 0 | 2 | 1 | 0 | 0 | 0 | 0 | 0 | 0 | 1 | 0 | 0 | 0 | 0 | 0 |
| **Basal Ganglia** | 0 | 0 | 0 | 0 | 0 | 0 | 0 | 0 | 0 | 0 | 0 | 0 | 1 | 0 | 0 | 0 | 0 | 0 |
| **Brainstem** | 0 | 0 | 0 | 0 | 0 | 0 | 0 | 0 | 0 | 0 | 0 | 0 | 1 | 0 | 0 | 0 | 0 | 0 |
| **Cerebellum** | 0 | 0 | 0 | 0 | 0 | 0 | 0 | 0 | 0 | 0 | 0 | 0 | 1 | 0 | 0 | 0 | 0 | 0 |
| **Cingulate** | 0 | 0 | 0 | 0 | 0 | 0 | 0 | 0 | 0 | 0 | 0 | 0 | 1 | 0 | 0 | 0 | 0 | 0 |
| **Claustrum** | 0 | 0 | 0 | 0 | 0 | 0 | 0 | 0 | 0 | 0 | 0 | 0 | 1 | 0 | 0 | 0 | 0 | 0 |
| **Frontal dlPFC** | 0 | 0 | 0 | 0 | 0 | 0 | 0 | 0 | 0 | 0 | 0 | 0 | 1 | 0 | 0 | 0 | 0 | 0 |
| **Frontal dmPFC** | 0 | 0 | 0 | 0 | 0 | 0 | 0 | 0 | 0 | 0 | 0 | 0 | 1 | 0 | 0 | 0 | 0 | 0 |
| **Frontal vlPFC** | 0 | 0 | 0 | 0 | 0 | 0 | 0 | 0 | 0 | 0 | 0 | 0 | 1 | 0 | 0 | 0 | 0 | 0 |
| **Frontal vmPFC** | 0 | 0 | 0 | 0 | 0 | 0 | 0 | 0 | 0 | 0 | 0 | 0 | 1 | 0 | 0 | 0 | 0 | 0 |
| **Fusiform** | 0 | 0 | 0 | 0 | 2 | 0 | 0 | 0 | 0 | 0 | 0 | 0 | 1 | 0 | 0 | 0 | 0 | 0 |
| **Hippocampus** | 0 | 0 | 0 | 0 | 0 | 0 | 0 | 0 | 0 | 0 | 0 | 0 | 1 | 0 | 0 | 0 | 0 | 0 |
| **Hypothalamus** | 0 | 0 | 0 | 0 | 0 | 0 | 0 | 0 | 0 | 0 | 0 | 0 | 1 | 0 | 0 | 0 | 0 | 0 |
| **Insula** | 0 | 0 | 0 | 0 | 0 | 0 | 0 | 0 | 0 | 0 | 0 | 0 | 1 | 0 | 0 | 0 | 0 | 0 |
| **Lingual Gyrus** | 0 | 0 | 0 | 0 | 0 | 0 | 0 | 0 | 0 | 0 | 0 | 0 | 1 | 0 | 0 | 0 | 0 | 0 |
| **Occipital Cuneus** | 0 | 0 | 0 | 0 | 0 | 0 | 0 | 0 | 0 | 0 | 0 | 0 | 1 | 0 | 0 | 0 | 0 | 0 |
| **Occipital V2** | 0 | 0 | 0 | 0 | 0 | 0 | 0 | 0 | 0 | 0 | 0 | 0 | 1 | 0 | 0 | 0 | 0 | 0 |
| **Occipital V1** | 0 | 0 | 0 | 0 | 0 | 0 | 0 | 0 | 0 | 0 | 0 | 0 | 1 | 0 | 0 | 0 | 0 | 0 |
| **OFC** | 0 | 0 | 0 | 0 | 0 | 0 | 0 | 0 | 0 | 0 | 0 | 0 | 1 | 0 | 0 | 0 | 0 | 0 |
| **Parahippocampus** | 0 | 0 | 0 | 0 | 0 | 0 | 0 | 0 | 0 | 0 | 0 | 0 | 1 | 0 | 0 | 0 | 0 | 0 |
| **Parietal Inferior** | 0 | 0 | 0 | 0 | 0 | 0 | 0 | 0 | 0 | 0 | 0 | 0 | 1 | 0 | 0 | 0 | 0 | 0 |
| **Parietal Superior** | 0 | 0 | 0 | 0 | 0 | 0 | 0 | 0 | 0 | 0 | 0 | 0 | 1 | 0 | 0 | 0 | 0 | 0 |
| **Precuneus** | 0 | 0 | 0 | 0 | 0 | 0 | 0 | 0 | 0 | 0 | 0 | 0 | 1 | 0 | 0 | 0 | 0 | 0 |
| **Temporal Inferior** | 0 | 0 | 0 | 0 | 0 | 0 | 0 | 0 | 0 | 0 | 0 | 0 | 1 | 0 | 0 | 0 | 0 | 0 |
| **Temporal Middle** | 0 | 0 | 0 | 0 | 0 | 0 | 0 | 0 | 0 | 0 | 0 | 0 | 1 | 2 | 0 | 0 | 0 | 0 |
| **Temporal Pole** | 0 | 0 | 0 | 0 | 0 | 0 | 0 | 0 | 0 | 0 | 0 | 0 | 1 | 0 | 0 | 0 | 0 | 0 |
| **TemporalSuperior** | 0 | 0 | 0 | 0 | 0 | 0 | 0 | 0 | 0 | 0 | 0 | 0 | 1 | 2 | 0 | 0 | 0 | 0 |
| **Thalamus** | 0 | 0 | 0 | 0 | 0 | 0 | 0 | 0 | 0 | 0 | 0 | 0 | 1 | 0 | 0 | 0 | 0 | 0 |
| **NOSIGDIF** | 0 | 0 | 0 | 0 | 0 | 0 | 0 | 0 | 0 | 0 | 0 | 0 | 1 | 0 | 0 | 0 | 0 | 0 |
