# Supplementary material for: Do You Match What I Match? A Systematic Review of Over 20 Years of the Emotional Face Matching Task Reveals Vast Inconsistencies in Design and Implementation
Source: Hum Brain Mapp. 2026 Sep 21;47(14):e70636. doi: 10.1002/hbm.70636 (PMC13594551; doi:10.1002/hbm.70636)
Supplement: Supplementary file 2 [file HBM-47-e70636-s002.docx]

**Identification**

Reports identified by citation chaining during full text screening

(n = 11)^**^

Reports excluded (n = 440)

*Incorrect task (n = 273)*

*Contrasts of interest not reported / Study design not compatible*

*(n = 106)*

*Patient group not of interest, and no healthy control only results reported (n = 16)*

*Intervention not of interest, and no placebo only control results reported (n = 15)*

*Wrong publication type (n = 16)*

*Other (n = 14)*

Reports not retrieved (n = 1)^*^

Records excluded

(n = 7108)

Records removed before screening:

*Duplicate records removed (n = 1340)*

*Records removed for other reasons*

*Reprints (n = 6)*

**Studies included in review**

**(n = 179)**

Original Hariri manuscripts (n = 3)

**Included**

**Screening**

**Identification**

Records screened

(n = 7717)

Reports assessed for eligibility

(n = 608)

Reports sought for retrieval

(n = 609)

Records identified from:

Databases (n = 9063)

Scopus _search terms_ (n = 7215)

Web of Science (n = 419)

PsycINFO (n = 364)

PubMed (n = 445)

Scopus _citations_ (n = 620)

***Supplementary Figure 1:* PRISMA Flow Diagram.** The flow diagram illustrates the flow of information through the different phases of the systematic review. It maps out the number of records identified, included and excluded, and the reasons for exclusions. Titles and abstracts were screened and full texts were assessed for eligibility. ^*^Manuscript inaccessible, and corresponding author did not respond to request to provide a copy of the published manuscript. ^**^3 of these added manuscripts were excluded during initial identification as the task description seemed irrelevant from the abstract.

***Supplementary Figure 2:* Terminology example:** A single article with two samples/studies (A, B), where study A has 2 groups (A.1 and A.2). Participants in group A.1 are scanned using two different scanners (S1 and S2). Scanner 1 used two different settings for their scanning parameters (AP1 and AP2) over the course of the study. All data in this manuscript is preprocessed in the same way. The manuscript reports significant activations for the face>shapes contrast for group A.1 and A.2 alone, as well as for Sample A > Sample B.

***Supplementary Figure 3:* Legend for Figure 3b: Task stimuli and instructions:** Each row of dots represents the stimuli used in a task (total n = 184). For each task parameter (column) a change in colour indicates a different stimuli from the original task design. Note there is no inherent ordering to the colours, rather they reflect different categorical variables. The corresponding colour for each stimulus or instruction is detailed below.

 ***Supplementary Figure 4:* Heterogeneity of scanners, acquisition parameters and preprocessing software used in included studies. (a) Scanner type.** In the 184 scans reported 32 unique scanners used. The most frequently used MRI models are highlighted with their respective percentage as a proportion of the number of scans (n = 184 (+NA). **(b) Acquisition parameters.** Each bar illustrates the relative proportion of scanning parameters (total n = 184) with the respective value of the different settings: Time-to-Echo (TE), Repetition Time (TR), Flip angle (°), Field of view (FOV; mm), Matrix size (mm2). The width of each stream between the bars represents the total number of samples with those parameters. **c) Preprocessing software used.** In the 183 included manuscripts, there were 31 different pre-processing softwares used. The most frequently used software versions are highlighted with their respective percentage as a proportion of the number of pre-processing softwares.
