# Supplementary material for: Do You Match What I Match? A Systematic Review of Over 20 Years of the Emotional Face Matching Task Reveals Vast Inconsistencies in Design and Implementation
Source: Hum Brain Mapp. 2026 Sep 21;47(14):e70636. doi: 10.1002/hbm.70636 (PMC13594551; doi:10.1002/hbm.70636)
Supplement: Supplementary file 3 [file HBM-47-e70636-s003.docx]

**Supplementary Information**

## 2.1 Study Identification

### 2.1.1 Search Terms

a.	Scopus
ALL ( ( "emotional face matching" OR "emotional face processing" OR "facial emotion processing" OR "face matching" OR "emotion matching" ) AND ( "magnetic resonance" OR "Functional magnetic resonance imaging" OR "fMRI" ) ) AND PUBYEAR > 2001 ( LIMIT-TO ( DOCTYPE , "ar" ) )

b.	Web of Science
((ALL=("emotional face matching" OR "emotional face processing" OR "facial emotion processing" OR "face matching" OR "emotion matching")) AND ALL=(( "magnetic resonance" OR "Functional magnetic resonance imaging" OR "fMRI" )))

Timespan: 2002-01-01 to 2026-02-17 (Publication Date)

c.	PsycINFO
(emotional face matching OR emotional face processing OR facial emotion processing OR face matching OR emotion matching) AND (magnetic resonance OR Functional magnetic resonance imaging OR fMRI)

Limit to yr ="2002 – 2026"”

d.	PubMed
("emotional face matching"[All Fields] OR "emotional face processing"[All Fields] OR "facial emotion processing"[All Fields] OR "face matching"[All Fields] OR "emotion matching"[All Fields]) AND ("magnetic resonance"[All Fields] OR "Functional magnetic resonance imaging"[All Fields] OR "fMRI"[All Fields]) AND 2002/01/01:2026/02/17 [Date - Publication]

## 2.2 Data extraction

### 2.2.1 Brain Regions Reported Active

The contrasts of interest included (1) Controls: Faces > Shapes, (2) Patients: Faces > Shapes, (3) Patients > Controls: Faces > Shapes, (4) Patients < Controls: Faces > Shapes. Each contrast was then binned according to the stringency of the statistical threshold: 1 = ROI/SVC: P uncorrected, 2 = ROI/SVC: FDR corrected, 3 = ROI/SVC: family-wise error [FWE] corrected + Simulation, 4 = ROI/SVC: Other (unspecified multiple corrections), 5 = Whole brain: uncorrected, 6 = Whole brain: false discovery rate [FDR] correction, 7 = Whole brain: FWE corrected + Simulation, 8 = Whole brain: Other (unspecified multiple corrections), and 9 = Other.

There was no evidence of lateralisation in the brain regions reported (Fishers Exact Test *p* = 0.948), and as such brain regions were further collapsed across left and right hemispheres to a total of 28 regions: amygdala, hippocampus, parahippocampus, thalamus, hypothalamus, basal ganglia, cingulate, claustrum, entorhinal, dorsolateral prefrontal cortex, dorsomedial prefrontal cortex, ventrolateral prefrontal cortex, ventromedial prefrontal cortex, fusiform gyrus, insula, lingual gyrus, cuneus, secondary visual cortex, primary visual cortex, inferior parietal lobe, precuneus, superior parietal lobe, inferior temporal gyrus, middle temporal gyrus, superior temporal gyrus, temporal pole, the brain-stem and cerebellum. For a comprehensive list of the sub-regions/region names that contributed to each of the 28 regions see also Supplementary Tables 3a-3f.

## 3. Results

## Note on terminology used in manuscript:

**Articles:** Number of manuscripts that were identified through screening.

**Study/Sample:** Experimental population. Applicable when an article includes results from two independent study populations.

**Group:** Experimental groups within each independent study/sample. For example:

Experiment 1 - Control group 1

Experiment 1 - Control group 2

**Contrasts/Reports:** Reported contrasts of interest. Applicable when an article reports relevant activation for multiple contrasts of interest. For example, reporting Fearful faces > Shapes and Angry faces > Shapes, would mean two contrasts/reports for the single article.

See Supplementary Figure 2 below for an example of the terminology used.

## 3.1 Scanning Parameters

### 3.1.1 Scanner Type and strength

Of the 9 different Siemens machines, the MAGNETOM TIM Trio was the most popular, being used for 20.63% of scans. General Electric (GE) was the second most popular vendor (31.74%), followed by Philips (14.28%) and Bruker machines (3.17%). The most frequently used GE scanners were the Signa (8.99%), the Signa Excite (5.82%) and the MR750 (4.76%). The Philips Gyroscan Intera was used in 7.41% of scanning sites. Six sites (3.17%) did not report any details of the machine vendor (See Supplementary Figure 4a - white inner and outer circle), and 25 (13.23%) provided the brand of the machine, but did not provide more information about the specific model (Supplementary Figure 4a - light grey outer circle). 1.5T scanners were used 14 times (7.61%), 7T were used 4 times (2.17%), and a 2T was used once (0.54%). The scanner strength was not reported 3 times (1.63%).

### 3.1.2. Acquisition Parameters

The most common combination of scanning parameters, used 10 times (5.05%), was used on a 3T scanner with the following settings: 30ms TE, 2100ms TR, 90° flip angle, a 64×64 matrix, 34 slices, a voxel size of 3.6×3.6×3.6mm, with additional details about the FOV, the number of volumes, slice thickness, and slice gap not specified. See Supplementary Figure 4b for an alluvial plot illustrating the frequency of the use of relative scanning parameters. This review did not account for the large heterogeneity in MRI pulse sequences used, which could represent an additional source of variation in the brain regions activated.

***TE/TR:*** Twenty-five different echo time (TE) and 30 repetition time (TR) durations were reported. A TE of 30ms and a TR of 2000ms were used most frequently (n = 77, 38.89% and n = 95, 47.98%), whereby a TE/TR combination of 30ms/2000ms was commonly applied (21.72%), followed by 25ms/2000ms (6.57%) and 30ms/2100ms (5.56%). TE and TR were not reported 4 times each (2.31%).

***Flip Angle:*** Twenty-three different flip angles, ranging from 13-180°, were reported. A flip angle of 90° was most frequently used across studies (n = 70, 40.46%), though this parameter was not reported 35 times (20.23%).

***Field of View (FOV):*** Twenty-seven unique FOV sizes were mentioned in total, with 240x240mm the most commonly reported dimensions (n = 33, 16.67%). Most frequently, however, the FOV size was not reported (n = 54, 27.27%).

***Matrix Size:*** Twenty different matrix sizes were reported, with 64×64mm being used most consistently (n = 106, 50.54%). Dimensions ranged from [64-220mm] - [64-256mm], and it was not specified 65 times (32.83%). A third matrix dimension was reported 3 times, ranging from [5-30mm].

***Number of Volumes:*** The majority of manuscripts did not specifically report the number of brain volumes acquired (n = 120, 61.61%), despite this often indirectly corresponding to task length and TR. Fifty different numbers of volumes were reported in total, with 256 volumes most frequently acquired (n = 11, 5.56%).

***Slice Parameters:*** The number of slices acquired was most frequently not specified (n = 36, 18.18%). In total, 32 different numbers of slices were reported and 34 slices were acquired most frequently (n = 26, 13.13%). Both the slice thickness and the gap between slices were frequently not reported (n = 56, 28.28% and n = 54, 27.27%). There were 19 different slice thickness and 15 different gap variations, the most common ones being a 3mm slice thickness (n = 52, 26.26%) and no gap (n = 76, 38.38%).

***Voxel Size:*** The voxel size was not reported in the majority of cases (n = 97, 48.99%). Forty-one different voxel sizes were reported, with 3×3×3mm being used most commonly (n = 21, 10.61%).

### 3.1.3 Processing Software

Preprocessing was performed using SPM in 127 (69.39%) of the 183 manuscripts; the most frequently used version being SPM8 (55 articles, 30.05%), followed by SPM12 (33 articles, 18.03%), SPM5 (15 articles, 8.19%) and SPM2 (12 articles, 6.56%). SPM96 and SPM99 were each used 1 (0.54%) and 6 times, respectively (3.27%). 4 articles (2.18%) did not report the version of SPM they used to perform their pre-processing. FSL was the second most popular software, used in 33 manuscripts (18.03% of articles), with version 6.0 (5 articles, 2.73%) the most frequently used. Nineteen articles did not report the FSL software version used (10.38%). AFNI was used in 19 articles (10.38%), none of which further specified the version details, and of which five combined its use with SPM12, SPM12 and ANTS, FSL, or FSL and ANTS. AIR was used in 2 studies (1.09%). ANTS was used in the aforementioned combinations , or with FSL in a total of 4 manuscripts (2.18%). ART was used in 3 (1.63%) manuscripts, always in addition to an SPM pipeline (2 SPM8; 1 SPM12), each applied in three manuscripts. 3 articles reported using in-house scripts (1.63%), all of which used this code in combination with FSL. BrainVoyager 2000 and BrainVoyager QX1/9 were each used once (0.54%), as was the fMRwhy toolbox which was used once with SPM12 (0.54%). There were in total 30 unique pre-processing software combinations and 15 manuscripts used more than one software package (8.19%). See Supplementary Figure 4c for a summary of relative frequencies.

## 3.2 Brain Regions Activated

### 3.2.1 Regions Activated - Patient Samples

***Major Depressive Disorder:*** The amygdala, despite being frequently specified as an ROI, exhibited significantly increased activation in MDD patients compared to controls only 4 out of 25 times when FWE correction was applied to the small volume of interest (16.00%). Interestingly, no contrasts reported increased amygdala activation when whole brain activation maps were FWE corrected. With regards to other subcortical regions, only the hippocampus and parahippocampal gyrus were selected as ROIs and each was reported to have increased activation 2 times (FWE SVC; 50%) and 1 time (FWE SVC; 100%) respectively. On a whole brain level, only the parahippocampal gyrus showed increased activation in the patient group, as compared to the respective control group (n = 1, 5.88%).

Despite the frontal regions being frequently selected ROIs in studies with a group of participants with MDD (dmPFC: n = 6, dlPFC, vlPFC and vmPFC: n = 5 and cingulate cortex: n= 4), patients reportedly did not show increased activation compared to healthy controls within this ROI. The increased activation within the cingulate cortex, however, survived stringent FWE correction to whole brain activation in 14 out of 17 studies (82.35%). Other frontal regions sporadically showed increased activation on a whole brain corrected level (dmPFC: n = 4 times, 23.53%; dlPFC: n = 2, 11.76%; OFC: n = 1, 5.88%).

Among the temporal regions, only the insula was investigated as a ROI (n = 4). Significantly increased activation in MDD patients was, however, observed only 1 out of 2 times when FWE correction was applied (50.00%). On a whole brain level, the insula, temporal inferior gyrus, and temporal superior gyrus each showed increased activation once (5.88%).

While parietal, occipital, cerebellar, and brainstem regions were not frequently selected as ROIs, increased activation was observed occasionally in contrasts that reported FWE corrected whole brain activation (precuneus: n = 2, 11.76%; superior parietal gyrus: n = 3, 17.65%; lingual gyrus: n = 2, 11.76%; cuneus, primary and secondary visual areas: n = 1, 5.88%).

Eleven studies that thresholded whole brain contrasts using stringent FWE correction did not observe significantly greater activation in MDD patients, relative to control groups, in any brain region (n = 11, 64.70%).

***Anxiety-Related Disorders:*** Similarly to the results observed in MDD, the amygdala showed increased activation in patients with ARD, as compared to controls, in only 7 of 26 instances when specified as a ROI (with FWE correction; 18.75%) and it did not survive FWE correction in whole brain contrasts. Again, only the hippocampus and parahippocampal gyrus were specified as additional ROIs among the subcortical regions, whereby each showed increased activation 2 times (FWE SVC; 50.00%) and one time (FWE SVC; 100.00%), respectively. None of the other subcortical regions exhibited increased activation in ARD patients compared to controls.

Frontal regions were generally not selected as ROIs, except for the cingulate cortex (10 times in total, and all of them with ROI FWE correction), which did not exhibit increased activation in ARD patients. Notably, frontal regions tended to be more frequently reported as active in whole brain FWE contrasts: compared to healthy controls, the cingulate cortex showed increased activation 3 times (30.00%), the dmPFC 4 times (40.00%), the dlPFC 2 times (20.00%) and OFC one time (10.00%).

The insula was the most frequently investigated ROI among the temporal regions (searched for 15 times in total). Comparable to the results observed in MDD patients, it showed significantly increased activation only 3 out of 10 times when FWE correction was applied (30.00%) and was not frequently reported to be active in whole brain FWE contrasts (n = 2, 20.00%). Additionally, the superior and inferior temporal gyri showed increased activation (n = 3, 30.00% and n = 1, 10.00%) when whole brain FWE corrected results were reported.

Again, parietal, occipital, cerebellar and brainstem regions were not selected as ROIs. Though, except for the brainstem, all of these regions occasionally showed significantly increased whole brain activation in ARD compared to healthy controls (precuneus: n = 4, 40.00%; superior parietal gyrus: n = 3, 30.00%; parietal inferior gyrus: n = 2, 20.00%; secondary visual areas: n = 3, 30.00%; lingual gyrus: n = 2, 20.00%; cerebellum: n = 2, 20.00%; cuneus and primary visual areas: n = 1, 10.00%).

Of the 10 contrasts that were whole brain and FWE corrected, no significant difference in brain activation between ARD and healthy controls was observed 4 times (40.00%).

***Obsessive-Compulsive Disorder:*** Increased amygdala activation in OCD patients was found 4 out of 6 times when selected as an ROI with FWE correction (66.67%). Mirroring the results observed in MDD and ARD, differences in activation did not survive whole brain FWE correction in studies which applied this more stringent threshold. Moreover, increased activation in the hippocampus and parahippocampal gyrus was observed 2 times and 1 time respectively when selected as ROIs (FWE SVC; 100.00%). Other subcortical regions were not investigated as ROIs and no significant differences were reported in corrected whole brain contrasts.

Among the frontal brain regions, only the cingulate cortex and OFC were specified as ROIs (2 times each), however, no increased activation in patient groups was observed. On a FWE corrected whole brain level, the dmPFC, dlPFC and OFC showed increased activation in patients compared to the controls (dmPFC: n = 2, 100.00%; dlPFC and OFC: n = 1, 50.00%).

The insula was the only temporal region that was specified as an ROI (6 times in total), however, increased activation was observed in merely 1 out of 4 instances when ROI FWE correction was applied (25.00%) and 1 out of 2 times in whole brain FWE corrected contrasts (50.00%). Other temporal regions were not selected as ROIs and only the superior temporal gyrus showed an increase in activation on a corrected whole brain level (n = 1, 50.00%).

Following the patterns observed in MDD and ARD, parietal, occipital, cerebellar and brainstem regions were not selected as ROIs. The superior parietal gyrus, primary visual areas, and the lingual gyrus showed increased activation in FWE corrected whole brain activation maps once (50.00%).

Of the 2 contrasts comparing patient activation to relative controls using a stringent FWE corrected whole brain threshold, one did not show any significant activation differences in any brain region (50.00%).

***Bipolar Disorder:*** Aside from the amygdala, only the cingulate cortex was specified as an additional ROI (1 time), however no FWE correction was applied. Parietal, temporal, occipital, cerebellar, and brainstem regions were not selected as ROIs. On a whole brain level, increased activation in BP patients was sporadically observed in the cingulate cortex (n = 2, 40.00%), the dlPFC (n = 3, 60.00%), the basal ganglia, and secondary visual areas (n = 1, 20.00%). No significant difference in brain activation was reported 3 out of 5 times that reported FWE corrected whole brain activation (60.00%).

***Schizophrenia Spectrum Disorders:*** The amygdala (10 times) and frontal regions were primarily specified as ROIs (vmPFC: n = 8, dmPFC: n = 4, cingulate cortex: n = 3, dlPFC: n = 2). Further ROIs included the hippocampus (n = 2), the parietal inferior gyrus (n = 2), and the temporal middle gyrus (n = 1). Stringent error correction was rarely applied, and among those that did, no activation differences between patients with schizophrenia spectrum disorders and healthy controls were observed. No investigations comparing patients with schizophrenia spectrum disorders to controls on a whole brain level were carried out.

***Borderline Personality Disorder:*** The amygdala was most frequently selected as a ROI (2 times FWE corrected) but was never reported to be significantly more activated in BPD than in the relative control group. Other specified ROIs include the hippocampus (n = 2) and frontal regions (n = 2), excluding the OFC. No stringent error correction was applied for the aforementioned ROIs and no whole brain comparisons were carried out.

***Autism Spectrum Disorder:*** Frontal areas, visual processing areas, and the amygdala were most frequently selected as ROIs (vlPFC and the fusiform gyrus: n = 4, amygdala: n = 5 times, dlPFC, dmPFC and vmPFC: n = 2), followed by regions in the parietal and temporal lobe (precuneus, superior and middle temporal gyrus: n = 2), however, no FWE correction was applied for either ROI or whole brain analyses.

***Attention Deficit Hyperactivity Disorder:*** The amygdala was the only ROI specified to investigate differences between patients diagnosed with ADHD and respective controls. No increase in amygdala activation was observed when FWE correction was applied (n = 2). An increase in activation in ADHD patients, compared to healthy controls, was observed in the secondary visual cortex (n = 2, 100.00%), dmPFC and the precuneus (n = 1, 50.00%) in studies that applied whole brain FWE correction, however one other study that used the same whole brain threshold did not report significant differences in any region (50.00%).

***Substance Use Disorder:*** Activation differences in SUD patients were investigated only on a whole-brain level. Increased activation was observed in the cingulate cortex (n = 2, 100%), however two studies reported no significant differences in activation between SUD patients and healthy controls across the brain when FWE correction was applied.

***Gender Dysphoria:*** Activation differences were investigated on a corrected whole brain level only once, revealing no increased activation in patients with gender dysphoria compared to healthy controls.

***Premenstrual Dysphoric Disorder:*** In studies that applied whole-brain FWE correction, increased activation in PMDD patients relative to healthy controls was observed in the cingulate cortex, dlPFC, insula (n = 2, 100%), the basal ganglia, and precuneus (n = 1, 50%). One study found no significant whole-brain differences between the groups when FWE correction was applied.

***Body Dysmorphic Disorder:*** The amygdala and fusiform gyrus were the only regions selected as ROIs (2 times FWE corrected). Of these, the amygdala showed significantly increased activation in BDD patients compared to healthy controls (n = 1, 50%). On the whole-brain level, increased activation was observed in the middle and superior temporal gyri when the stringent error correction was applied (n = 2, 200%).
